# Maternal adverse childhood experiences and prenatal stress: Intergenerational transmission and offspring mental health in the ECHO Cohort

**DOI:** 10.1017/S0033291725103127

**Published:** 2026-03-11

**Authors:** Shaikh I. Ahmad, Alexandra D.W. Sullivan, Marie L. Churchill, Rosa M. Crum, Amanda N. Noroña-Zhou, Nora K. Moog, Patricia A. Brennan, Emily S. Barrett, Rebecca J. Schmidt, Claudia Buss, Leslie D. Leve, Michael A. Coccia, Judy L. Aschner, Lyndsay A. Avalos, Theresa M. Bastain, Lisa Croen, Dana Dabelea, Anne L. Dunlop, Michelle Bosquet Enlow, Assiamira Ferrara, Alison E. Hipwell, Akram N. Alshawabkeh, Kristen Lyall, Daphne Koinis-Mitchell, Thomas G. O’Connor, Emily Oken, Hudson P. Santos, Rosalind J. Wright, Jessica Arizaga, Su H. Chu, Heather Derry-Vick, Karen M. Tabb, Christine W. Hockett, Rachel S. Kelly, Brooke G. McKenna, John D. Meeker, Kaja Z. LeWinn, Nicole R. Bush, for the ECHO Cohort Consortium

**Affiliations:** 1 Department of Psychiatry and Behavioral Sciences, University of California, San Francisco, USA; 2 Department of Epidemiology, Johns Hopkins Bloomberg School of Public Health, Baltimore, USA; 3 Center for Health and Community, University of California, San Francisco, USA; 4 Institute of Medical Psychology, Heidelberg University, Heidelberg, Germany; 5 Charité–Universitätsmedizin Berlin, Freie Universität Berlin and Humboldt-Universität zu Berlin, Institute of Medical Psychology, Berlin, Germany; 6 Department of Psychology, Emory University, Atlanta, USA; 7 Rutgers School of Public Health, Piscataway, USA; 8 Environmental and Occupational Health Sciences Institute, Rutgers University, Piscataway, USA; 9 Department of Public Health Sciences, MIND Institute, University of California Davis School of Medicine, Davis, USA; 10 Department of Pediatrics, University of California Irvine, Irvine, USA; 11 German Center for Child and Adolescent Health, Berlin, Germany; 12 German Center for Mental Health, Berlin, Germany; 13 Prevention Science Institute, University of Oregon, Eugene, USA; 14 Weill Institute for Neurosciences, University of California, San Francisco, San Francisco, USA; 15 Center for Discovery and Innovation, Hackensack Meridian School of Medicine, Nutley, USA; 16 Department of Pediatrics, Albert Einstein College of Medicine, Bronx, USA; 17 Division of Research, Kaiser Permanente Northern California, Pleasanton, USA; 18 Department of Population and Public Health Sciences, Keck School of Medicine, University of Southern California, Los Angeles, USA; 19 Lifecourse Epidemiology of Adiposity and Diabetes (LEAD) Center, University of Colorado Anschutz Medical Campus, Aurora, USA; 20 Department of Gynecology & Obstetrics, Emory University School of Medicine, Atlanta, USA; 21 Department of Psychiatry and Behavioral Sciences, Boston Children’s Hospital, Boston, USA; 22 Department of Psychiatry, Harvard Medical School, Boston, USA; 23 Department of Psychiatry, University of Pittsburgh, Pittsburgh, USA; 24 Department of Civil and Environmental Engineering, College of Engineering, Northeastern University, Boston, USA; 25 AJ Drexel Autism Institute, Drexel University, Philadelphia, USA; 26 Department of Pediatrics, Rhode Island Hospital, Warren Alpert Medical School at Brown University, Providence, USA; 27 Department of Psychiatry, University of Rochester Medical Center, Rochester, USA; 28 Department of Neuroscience, University of Rochester Medical Center, Rochester, USA; 29 Department of Obstetrics and Gynecology, University of Rochester Medical Center, Rochester, USA; 30 Harvard Medical School, Harvard Pilgrim Health Care Institute, Boston, USA; 31 University of Miami School of Nursing and Health Studies, Miami, USA; 32 Department of Public Health, Icahn School of Medicine at Mount Sinai, New York, USA; 33 Channing Division of Network Medicine, Brigham and Women’s Hospital and Harvard Medical School, Boston, USA; 34 Center for Discovery and Innovation, Hackensack Meridian Health, New York, USA; 35 School of Social Work, University of Illinois at Urbana-Champaign, Urbana, USA; 36 Avera Research Institute, Sioux Falls, USA; 37 Department of Pediatrics, University of South Dakota, School of Medicine, Sioux Falls, USA; 38 Center for Psychiatric and Neurodevelopmental Genetics Unit, Center for Genomic Medicine, Massachusetts General Hospital, Boston, USA; 39 Department of Environmental Health Sciences, University of Michigan School of Public Health, Ann Arbor, USA; 40 Department of Pediatrics, University of California, San Francisco, USA

**Keywords:** adverse childhood experiences, child mental health, child psychopathology, intergenerational transmission, pregnancy stress

## Abstract

**Background:**

The rising global prevalence of pediatric mental health problems requires the identification of preventable factors underlying their development. This study assessed whether maternal adverse childhood experiences (ACEs) and pregnancy stress were intergenerationally associated with offspring mental health.

**Methods:**

This study used data from 34 sites in the nationwide Environmental Influences on Child Health Outcomes Cohort. Eligible parent–child dyads (child age: 1.5–18 years) provided data on at least one measure of maternal stress and at least one measure of child mental health. Study aims were evaluated using regression analyses, including interaction tests to determine potential effect modifiers.

**Results:**

Participants were organized into three subsamples with data on (1) maternal ACEs (*N* = 2,906), (2) perceived prenatal stress (*N* = 4,441), and (3) both stress exposures (*N* = 834). After adjusting for confounders, maternal ACEs and prenatal stress were significantly associated with child mental health problems (*B* = 2.53 [95% confidence interval [CI]: 2.09, 2.96], *p* < 0.0001 and *B* = 2.36 [95% CI: 2.03, 2.68], *p* < 0.0001, respectively). Among participants with data on both stress exposures, maternal ACEs (*B* = 1.72, 95% CI: [0.96, 2.48], *p* < 0.0001) and prenatal stress (*B* = 2.05, 95% CI: [1.29, 2.80], *p* < 0.0001) were independently associated with child mental health problems. Neither maternal ACEs nor child sex modified the association between prenatal stress and child mental health problems.

**Conclusions:**

Maternal exposure to ACEs and pregnancy stress were associated with the development of child mental health problems. These findings highlight the need for policies and interventions that mitigate exposure to adversity and protect pregnant individuals and their children from the intergenerational transmission of mental health problems.

## Introduction

Global increases in pediatric mental health problems have moved prevention and early intervention efforts to the forefront of policy agendas (Benton, Boyd, & Njoroge, 2021; Keyes & Platt, 2023). Investigations into modifiable or preventable underpinnings of child mental health problems are critical for such efforts. Intergenerational transmission of the effects of stress during pregnancy has been supported in prior work, including evidence of preconception adversity (Su, D’Arcy, & Meng, 2022) and stress during pregnancy (Van den Bergh et al., 2020) in association with offspring neurodevelopment (Bush, 2024; Monk, Lugo-Candelas, & Trumpff, 2019). Etiologic links between these factors are consistent with the Developmental Origins of Health and Disease (DOHaD) and prenatal programming theories. Whereas elevated physiological activity in response to stress can be adaptive by supporting coping (Epel & Prather, 2018), traumatic or chronic stress responses may alter biological functioning in an effort to contend with perceived environmental demands (Anda et al., 2006; Buss et al., 2017; Glover, O’Connor, & O’Donnell, 2023; Hentges, Graham, Plamondon, Tough, & Madigan, 2019; Racine, Plamondon, Madigan, McDonald, & Tough, 2018; Van den Bergh et al., 2020).

Using a developmental psychopathology framework, maternal exposures to stress may constitute a risk factor in the offspring’s development of psychopathology (Doyle & Cicchetti, 2018). This intergenerational transmission likely occurs through both direct pathways (e.g. biological changes during gestation) and indirect pathways (e.g. changes in maternal behavior) (Moog et al., 2022). Exposures to various forms of stress and adversity appear to be particularly important to offspring neurodevelopment during ‘sensitive periods’, such as childhood and the perinatal period (Knudsen, 2004) – a time when multiple biological systems undergo rapid changes and are more malleable (Davis & Narayan, 2020; Entringer, Buss, & Wadhwa, 2012; Loman & Gunnar, 2010). These changes can include alteration of reproductive biology and fetal development, which not only potentially facilitates infant phenotypes that promote survivability (e.g. hypervigilance and highly responsive temperaments) but also can increase the risk for the development of psychopathology (Hartman & Belsky, 2018). For example, recent epidemiological findings indicate that maternal history of childhood maltreatment is related to greater internalizing mental health problems in offspring, such as depression and anxiety, in addition to autism spectrum disorder and diagnostic patterns reflecting multimorbidity (Bush, 2024; Moog et al., 2023). Additionally, maternal stress during pregnancy is associated with externalizing mental health conditions in offspring, such as attention-deficit/hyperactivity disorder (Ahmad et al., 2022; Szekely et al., 2021; Tung et al., 2023). Together, these findings emphasize the potential for *two key sensitive periods* – maternal childhood and pregnancy – during which maternal stress exposures may increase the risk for the transmission of effects across generations.

A few recent studies have included stress exposures during both sensitive periods in intergenerational transmission models of child mental health problems, but this research has yielded mixed evidence and has limitations (Bush et al., 2023; Noroña-Zhou et al., 2023). For example, these studies did not include a comprehensive assessment of maternal childhood adversity and did not assess offspring externalizing mental health problems or outcomes into adolescence. Moreover, extant findings suggested that biological parents exposed to early childhood adversity may be more sensitized to stress experienced during pregnancy, leading to stronger effects of prenatal stress on offspring (Bush et al., 2023); however, this requires replication and further study. Adverse childhood experiences (ACEs) represent a broad, multidomain assessment of childhood adversity and have been widely used in health research, given the association between ACEs and overall health and the translatability of ACEs into policy initiatives and healthcare contexts (Felitti et al., 1998; Shonkoff et al., 2012). ACE measures typically include exposures such as physical violence by a parent or other adult in the household, separation or divorce, or substance abuse by a household member. Overall, findings demonstrate a significant association between the number of ACEs experienced and higher levels of mental health problems (Hughes et al., 2017; Sahle et al., 2022; Zeanah & Sonuga-Barke, 2016). A smaller body of work also shows an association between maternal ACEs and child mental health problems (Racine et al., 2023). Large-scale epidemiological studies that consider multiple sensitive periods of intergenerational risk are lacking but are necessary to pinpoint effective prevention and intervention efforts. Indeed, few studies have simultaneously examined the effect of maternal ACEs and pregnancy stress exposure on offspring mental health, let alone in large, sociodemographically diverse samples; fewer still have examined these intergenerational associations into adolescence.

### Current study

This study leveraged the National Institutes of Health-funded Environmental influences on Child Health Outcomes (ECHO) Cohort, a large, racially, socioeconomically, and geographically diverse sample with data from 69 pregnancy and pediatric cohort sites across the United States (Blaisdell et al., 2022; Knapp et al., 2023). In the present study, we used data from 34 unique sites to assess associations between maternal ACE exposure, pregnancy stress, and offspring mental health problems to determine whether higher levels of maternal stress exposure occurring during a mother’s own childhood and/or pregnancy were associated with offspring mental health problems (Aim 1; see Supplementary Figure S1). Based on previous evidence demonstrating that maternal exposure to adversity during one’s own childhood was associated with stronger positive associations between pregnancy stress and offspring mental health problems (Bush et al., 2023), we also examined whether parental history of ACE exposure *moderated* the association between pregnancy stress and offspring mental health problems (Aim 2). Additionally, although meta-analytic reviews have concluded a lack of sex moderation for some of the associations tested (e.g. Tung et al. (2023) in predicting children’s externalizing symptoms), given the broader mixed evidence on sexually dimorphic associations between maternal stress exposures and offspring mental health problems (Hodes & Epperson, 2019; McCarthy, 2019; Sandman, Glynn, & Davis, 2013; Sutherland & Brunwasser, 2018; Tung et al., 2023), we also used two-way interactions to assess whether associations between prenatal stress or ACE exposure and offspring mental health were moderated by child sex (Aim 3). Consistent with a developmental psychopathology perspective (Hawes & Allen, 2023; Rutter, 1996), child age can be a key determinant of whether and how risk factors manifest as mental health problems. Given the wide age range of children in the present study, and given the complex manner in which child psychopathology can develop and manifest over time, we also evaluated two-way interactions assessing whether the associations between prenatal stress or ACE exposure and child mental health problems were moderated by child age (Aim 4). Also aligned with developmental psychopathological perspectives, these maternal stress exposures are likely associated with multifinal presentations of offspring mental health problems, and they may have a stronger impact on specific domains of psychopathology (Garber & Bradshaw, 2020). We thus conducted secondary analyses to evaluate whether associations between maternal stress exposures and offspring internalizing and externalizing mental health problems examined separately provided unique information beyond the examination of total problems, which has been suggested by some prior research (e.g. Letourneau et al., 2019; Moog et al., 2023; Noroña-Zhou et al., 2023; Roubinov et al., 2022), although rarely tested in the same study. Finally, to enhance the clinical utility of our findings, we tested whether maternal stress exposures distinguished risk for offspring mental health problems at lower versus clinically elevated levels.

## Methods

### Study design, participants, and procedure

We conducted analyses using data from the prospective, longitudinal ECHO Cohort. Detailed information regarding ECHO and its study enrollment, protocol, and procedures is available elsewhere (see https://echochildren.org/) (Blaisdell et al., 2022; Knapp et al., 2023). This study included 6,513 biological parent–child dyads enrolled in 34 ECHO sites from 19 US states, Washington, DC, and Puerto Rico (for details, see Supplement 1, page 3). For the present study, birthing parents (biological mothers) were recruited during pregnancy, and they and their offspring were then prospectively followed into childhood or adolescence. While visit schedule and protocol varied across cohort sites within ECHO, study protocols typically included a schedule of several prenatal visits, data collection to characterize birth and immediate postnatal outcomes (e.g. medical record review), and multiple postnatal visits with parent–child dyads. The study protocol was approved by the single ECHO institutional review board, WCG IRB. Written informed consent or parent’s/guardian’s permission was obtained, along with child assent as appropriate, for the ECHO Cohort Data and Biospecimen Collection Protocol participation and for participation in specific study sites.

The inclusion criteria consisted of (a) at least one measure of maternal stress exposure (perceived stress during pregnancy or ACEs) and (b) at least one measure of caregiver-reported child behavior from the Child Behavior Checklist (CBCL) or Strengths and Difficulties Questionnaire (SDQ). For families with more than one child enrolled in ECHO, one sibling was randomly selected to be included (see Supplementary Figure S1). Cohort sites were restricted to those with available data for at least five parent–child dyads. Participants in the present study were categorized into three groups for analytical purposes: those who provided information on maternal ACEs only (number of cohorts = 13), those who provided information on perceived stress during pregnancy only (number of cohorts = 14), and those who provided information on both exposures (number of cohorts = 7).

### Measures

#### Maternal ACEs

Birthing parents reported on ACEs during their first 18 years of life using one of two versions of the ACE questionnaire utilized within ECHO (see Supplements 2 and 3) (Felitti et al., 1998). Using near-identical language, both versions of the questionnaire capture the same 10 experiences of childhood adversity: physical abuse, emotional abuse, sexual abuse, physical neglect, emotional neglect, abuse of mother, separation or divorce of parents, substance abuse by a household member, mental illness of a household member, and incarceration of a household member. One version asks for responses to each of the 10 individual exposures separately, while the other requests a total count for seven exposures (with a maximum of ‘5 or more’ to protect privacy) and the remaining three individual exposures separately. To optimize sample size and combine data available across forms, endorsed items were summarized into a pseudo-continuous variable count score, ranging from 0 to 5 (with a score of 5 indicating 5 or more ACEs). In order to compare effect sizes between the two exposure variables and across samples, the ACE measure was mean-centered and standardized for analysis (for additional information, see Supplement 1, page 3).

#### Maternal prenatal stress

Birthing parents reported on stress during pregnancy via the Perceived Stress Scale (PSS) (Cohen, Kamarck, & Mermelstein, 1983). The PSS measures an individual’s perceived feelings of stress over the past month (e.g. ‘How often have you felt difficulties were piling up so high that you could not overcome them?’). Perceived stress is rated on a five-point Likert scale, ranging from (1) *Never* to (5) *Very often.* ECHO utilized three different versions of the PSS, including the 14-, 10-, and 4-item versions. Raw scores were converted to T-scores based on item response theory, with a mean of 50 and a standard deviation (SD) of 10, and were mean-centered and standardized for analysis. Evidence suggests that stress later in gestation may be more strongly associated with offspring emotional and behavioral problems (Weinstock, 2008); therefore, when multiple prenatal assessments were available, we included the questionnaire completed at the latest gestation time point.

#### Child mental health problems

Child mental health problems were reported by a parent or caregiver via two widely used measures: (a) the CBCL Preschool-Age form (1.5 to <6 years) or CBCL School-Age form (6 to <18 years) (Achenbach & Rescorla, 2001, 2000) or (b) the SDQ-2 (2 to <4 years), SDQ-4 (6 to <11 years), or SDQ-11 (11 to <18 years) (Goodman, 1997). Raw CBCL and SDQ scores were converted into age- and sex-specific CBCL T-scores that have been harmonized for analyses within ECHO (Mansolf, Blackwell, Cummings, Choi, & Cella, 2022). While using a single measure of child mental outcomes may offer certain methodological advantages (e.g. reducing systematic error), leveraging ECHO’s harmonization across two measures provides substantial benefits through increased data coverage. When children had multiple assessments, the latest questionnaire completed was selected for analyses to advance the evidence base on intergenerational stress transmission in older children, given the paucity of research examining associations between these maternal stress exposures and outcomes in older children and adolescents. Ninety-six percent of information on child behavior was provided by biological mothers, with 2% provided by biological fathers and 2% by another caregiver. Primary analyses utilized the Total Problems score. Secondary analyses examined Externalizing Problems and Internalizing Problems scores separately. We also examined effects for children below versus at or above T-scores of 60, which is considered to be in the borderline or clinically at-risk range.

#### Covariates

Using the literature on intergenerational stress transmission, we selected maternal and child covariates that may confound or function as mechanistic variables, accounting for the association between maternal stress exposures and child mental health outcomes. Variables were obtained from prenatal, birth, and postnatal assessments. The following self-reported parental covariates were included: prenatal smoking (yes/no), prenatal alcohol use (yes/no), and age at delivery. Self-reported education, marital status, household income, and depressive symptoms (from the harmonized NIH Patient-Reported Outcomes Measurement Information System [PROMIS] Depressive Symptoms Scale [Blackwell et al., 2021]) were assessed at the time point closest to the child outcome assessment. Child covariates included gestational age at birth, child sex assigned at birth (male and female), and child’s age at the time of the outcome assessment.

### Statistical analyses

Among the 34 cohorts contributing data to these analyses, three subsamples were created (Table 1 and Supplementary Figure S1). Two subsamples were created for participants with maternal ACE data (*N* = 2,906 from 20 cohorts) or maternal PSS data (*N* = 4,441 from 21 cohorts). A third subsample was created to assess the potential joint effects of prenatal stress and ACE exposures on child mental health and the moderating effect of prenatal stress on the association of ACEs with child mental health. This third subsample comprised those cohorts with both ACE and PSS data (*N* = 834 from 7 cohorts).

**Table 1.** Demographic information across study subsamples (N, % with data)

Characteristic	PSS subsample(*N* = 4,441)	ACEs subsample(*N* = 2,906)	ACEs + PSS subsample(*N* = 834)
Maternal smoking during pregnancy	3,881 (87.39%)	1,754 (60.36%)	581 (69.66%)
No	3,723 (95.93%)	1,646 (93.84%)	565 (97.25%)
Yes	158 (4.07%)	108 (6.16%)	16 (2.75%)
Maternal alcohol use during pregnancy	3,629 (81.72%)	1,868 (64.28%)	582 (69.78%)
No	2,706 (74.57%)	1,604 (85.87%)	483 (82.99%)
Yes	923 (25.43%)	264 (14.13%)	99 (17.01%)
Maternal age at delivery	4,440 (99%)	2,893 (99%)	833 (99%)
Mean, years (SD)	30.26 (5.49)	29.59 (6.19)	30.66 (5.34)
Maternal education	4,398 (99%)	2,891 (99%)	833 (99%)
Less than high school	248 (5.64%)	200 (6.92%)	60 (7.2%)
High school degree or equivalent	582 (13.23%)	473 (16.36%)	120 (14.41%)
Some college, no degree	1,194 (27.15%)	812 (28.09%)	153 (18.37%)
Bachelor’s degree	1,163 (26.44%)	596 (20.62%)	207 (24.85%)
Advanced degree and above	1,211 (27.54%)	810 (28.02%)	293 (35.17%)
Maternal marital status	4,267 (96.08%)	2,845 (97.9%)	818 (98.08%)
Married/cohabitating	3,353 (78.58%)	2,107 (74.06%)	674 (82.4%)
Single/not cohabitating	914 (21.42%)	738 (25.94%)	144 (17.6%)
Household income	3,890 (87.59%)	2,691 (92.6%)	771 (92.45%)
<$30,000	1,010 (25.96%)	628 (23.34%)	178 (23.09%)
$30,000–$49,999	488 (12.54%)	380 (14.12%)	106 (13.75%)
$50,000–$74,999	552 (14.19%)	360 (13.38%)	96 (12.45%)
$75,000–$99,999	453 (11.65%)	272 (10.11%)	82 (10.64%)
$100,000–$199,999	924 (23.75%)	757 (28.13%)	207 (26.85%)
$200,000 or more	463 (11.9%)	294 (10.93%)	102 (13.23%)
Maternal self-identified ethnicity	4,436 (99%)	2,881 (99%)	833 (99%)
Hispanic	1,579 (35.6%)	568 (19.72%)	321 (38.54%)
Non-Hispanic	2,857 (64.4%)	2,313 (80.28%)	512 (61.46%)
Maternal self-identified race	4,232 (95.29%)	2,808 (96.63%)	827 (99%)
White	2,481 (58.62%)	1,984 (70.66%)	645 (77.99%)
Black	781 (18.45%)	525 (18.7%)	86 (10.4%)
Asian	365 (8.62%)	87 (3.1%)	33 (3.99%)
Native Hawaiian or other Pacific Islander	20 (<1%)	10 (<1%)	<5
American Indian/Alaska Native	48 (1.13%)	23 (<1%)	<15
Multiple Races	208 (4.91%)	95 (3.38%)	31 (3.75%)
Other Race	329 (7.77%)	84 (2.99%)	19 (2.3%)
Maternal Depressive Symptoms T-score	4,069 (91.62%)	2,796 (96.21%)	825 (98.92%)
Mean (SD)	48.44 (9.10)	49.38 (8.81)	49.27 (8.76)
Count of maternal ACEs	N/A	2,906 (100%)	834 (100%)
0		1,031 (35.48%)	305 (36.57%)
1		619 (21.3%)	174 (20.86%)
2		381 (13.11%)	109 (13.07%)
3		237 (8.16%)	69 (8.27%)
4		182 (6.26%)	54 (6.47%)
5 or more		456 (15.69%)	123 (14.75%)
Maternal prenatal PSS T-score	4,441 (100%)	N/A	834 (100%)
Mean (SD)	47.36 (10.49)		48.52 (9.22)
PSS instrument, *N* (%) with data	4,441 (100%)	N/A	834 (100%)
4 items	702 (15.81%)		0 (0%)
10 items	3,297 (74.24%)		709 (85.01%)
14 items	442 (9.95%)		125 (14.99%)
Gestational age (in weeks) at PSS	4,441 (100%)	N/A	834 (100%)
Mean (SD)	23.86 (8.92)		29.79 (4.21)
Child sex at birth	4,441 (100%)	2,906 (100%)	834 (100%)
Male	2,290 (51.56%)	1,502 (51.69%)	440 (52.76%)
Female	2,151 (48.44%)	1,404 (48.31%)	394 (47.24%)
Child gestational age at birth (weeks)	4,441 (100%)	2,829 (97.35%)	834 (100%)
Mean (SD)	38.77 (1.84)	37.97 (3.56)	38.97 (1.70)
Child age at outcome (years)	4,441 (100%)	2,906 (100%)	834 (100%)
Mean (SD)	4.91 (2.65)	8.55 (4.89)	5.43 (2.79)
Child outcome Total T-score	4,437 (99%)	2,906 (100%)	834 (100%)
Mean (SD)	45.48 (11.13)	47.39 (12.49)	46.81 (11.25)
T-score < 60	3960 (89.2%)	2427 (83.5%)	
T-score ≥ 60	477 (10.7%)	479 (16.5%)	
Child outcome Internalizing T-score	4,438 (99%)	2,665 (91.71%)	834 (100%)
Mean (SD)	45.77 (11.02)	48.86 (12.07)	47.58 (10.99)
T-score < 60	3893 (87.7%)	2125 (73.1%)	
T-score ≥ 60	545 (12.3%)	540 (18.6%)	
Child outcome Externalizing T-score	4,440 (99%)	2,739 (94.25%)	834 (100%)
Mean (SD)	45.17 (10.63)	46.91 (12.01)	45.43 (10.21)
T-score < 60	4009 (90.3%)	2364 (81.3%)	
T-score ≥ 60	431 (9.7%)	375 (12.9%)	

*Note:* In accordance with ECHO’s publication and data use policy, cell sizes <5 are suppressed for privacy. ACEs = adverse childhood experiences; ECHO = Environmental influences on Child Health Outcomes; PSS = Perceived Stress Scale; SD = standard deviation.

Separate linear mixed-effects regression models were employed utilizing our continuous outcome variables to investigate the associations between maternal exposures and child outcomes within each of the three subsamples: maternal ACEs only (Table 2), maternal PSS only (Table 3), and maternal ACEs and PSS (Table 4). Similar to previous work (Bush et al., 2023; Noroña-Zhou et al., 2023), we used a three-step model-building approach within each of these samples to include covariates into our models. Model 1 included only exposure variables (ACEs and/or PSS) with random intercepts for cohort site membership. Model 2 provided the main model for testing the primary aim of the study (i.e. assessing maternal ACEs and prenatal stress exposures independently and jointly) and included adjustments for income, education, age at delivery, marital status, child sex, and child age at outcome, which are either potential confounds or precision covariates necessary for isolating associations between maternal stress and child mental health (see also Lund et al., 2018; Tien, Lewis, & Liu, 2020). Model 3 included all covariates in Model 2 and covariates of frequent interest to the scientific community that could be considered ‘on the mechanistic path’ between maternal exposure and child outcome, including adjustments for prenatal smoking and alcohol use, child gestational age at birth, and maternal depressive symptoms (Supplement 1, Supplementary Tables S2–S10). We further assessed the odds of these maternal stress exposures being associated with offspring risk for mental health problems in the borderline or clinically significant range using mixed-effects logistic regressions predicting T-score cutoffs, including all Model 2 covariates, to determine the relation between each exposure and the odds of a child having problems at or above the borderline range. Missing data ranged from 0 to 12.4% in our primary model (Model 2) and from 1 to 39% in our extended model (Model 3). Missing covariates and confounders were imputed 25 times using the ‘mice’ package with a maximum of 10 iterations, with all study variables being included in the imputation model. Regression estimates and conditional/marginal *R* ^2^ values were pooled and calculated by applying Rubin’s Rule.

**Table 2.** Regression models of maternal adverse childhood experiences and child total problems score, N = 2,906

Model	Predictor	*b*	95% CI	*P*
Model 1- Minimally adjusted^b^				
	Standardized maternal ACEs^a^	2.78	[2.34, 3.21]	<0.0001
				
Model 2a- Fully adjusted^c^				
	Standardized maternal ACEs^a^	2.53	[2.09, 2.96]	<0.0001
	Household income			
	$30,000–$49,999	−0.77	[−2.28, 0.73]	0.31
	$50,000–$74,999	−2.61	[−4.23, −0.98]	0.002
	$75,000–$99,999	−3.04	[−4.92, −1.17]	0.001
	$100,000–$199,999	−3.63	[−5.32, −1.94]	<0.0001
	$200,000 or more	−3.98	[−6.08, −1.89]	<0.001
	Maternal education			
	High school degree or equivalent	−2.78	[−4.73, −0.84]	0.005
	Some college, no degree	−2.09	[−3.98, −0.20]	0.03
	Bachelor’s degree	−1.84	[−3.88, 0.20]	0.08
	Advanced degree and above	−2.52	[−4.6, −0.43]	0.02
	Maternal age at delivery	−0.07	[−0.16, 0.01]	0.09
	Maternal marital status			
	Single	1.15	[−0.03, 2.32]	0.06
	Child sex			
	Female	−1.80	[−2.65, −0.96]	<0.0001
	Child’s age at outcome	−0.33	[−0.51, −0.15]	<0.001

*Note:* ECHO cohort study site included as random intercept in all models. Unless noted (i.e. maternal ACEs), *b* values are unstandardized beta coefficients. ACEs = adverse childhood experiences; CI = confidence interval; ECHO = Environmental influences on Child Health Outcomes.

a Maternal ACEs are standardized (*β*).

b Minimally adjusted model accounted for 16% of variance in child Total Problems score.

c Fully adjusted model accounted for 21% of variance in child Total Problems score.

**Table 3.** Regression models of maternal prenatal stress and child total problems, N = 4,437

Model	Predictor	*b*	95% CI	*P*
Model 1- Minimally adjusted^b^				
	Standardized prenatal PSS^a^	2.51	[2.18, 2.83]	<0.0001
				
Model 2a- Fully adjusted^c^				
	Standardized prenatal PSS^a^	2.36	[2.03, 2.68]	<0.0001
	Household income			
	$30,000–$49,999	−1.25	[−2.46, −0.04]	0.04
	$50,000–$74,999	−1.03	[−2.27, 0.21]	0.10
	$75,000–$99,999	−1.36	[−2.82, 0.11]	0.07
	$100,000–$199,999	−2.18	[−3.56, −0.79]	0.002
	$200,000 or more	−2.14	[−3.75, −0.52]	0.01
	Maternal education			
	High school degree or equivalent	−1.89	[−3.48, −0.29]	0.02
	Some college, no degree	−1.59	[−3.11, −0.08]	0.04
	Bachelor’s degree	−1.99	[−3.61, −0.37]	0.02
	Advanced degree and above	−2.83	[−4.51, −1.14]	0.001
	Maternal age at delivery	−0.02	[−0.08, 0.05]	0.62
	Maternal marital status			
	Single	0.76	[−0.19, 1.70]	0.12
	Child sex			
	Female	−1.88	[−2.50, −1.26]	<0.0001
	Child’s age at outcome	0.26	[0.09, 0.44]	0.004

*Note:* ECHO cohort study site included as random intercept in all models. Unless noted (i.e. maternal ACEs), *b* values are unstandardized beta coefficients. CI = confidence interval; ECHO = Environmental influences on Child Health Outcomes; PSS = Perceived Stress Scale.

a Maternal ACEs are standardized (*β*).

b Minimally adjusted model accounted for 10% of variance in child Total Problems score.

c Fully adjusted model accounted for 11% of variance in child Total Problems score.

**Table 4.** Regression models of maternal adverse childhood experiences, maternal prenatal stress, and child total problems, N = 834

Model	Predictor	*b*	95% CI	*P*
Model 1- Minimally adjusted^b^				
	Standardized maternal ACEs^a^	2.03	[1.28, 2.78]	<0.0001
	Standardized prenatal PSS^a^	2.16	[1.40, 2.92]	<0.0001
				
Model 2a- Fully adjusted^c^				
	Standardized maternal ACEs^a^	1.72	[0.96, 2.48]	<0.0001
	Standardized prenatal PSS	2.05	[1.29, 2.80]	<0.0001
	Household income			
	$30,000–$49,999	−2.57	[−5.17, 0.03]	0.05
	$50,000–$74,999	−2.64	[−5.55, 0.27]	0.08
	$75,000–$99,999	−2.99	[−6.15, 0.17]	0.06
	$100,000–$199,999	−2.69	[−5.48, 0.11]	0.06
	$200,000 or more	−2.91	[−6.23, 0.40]	0.08
	Maternal education			
	High school degree or equivalent	−2.85	[−6.18, 0.48]	0.09
	Some college, no degree	−1.19	[−4.49, 2.10]	0.48
	Bachelor’s degree	−1.75	[−5.22, 1.73]	0.32
	Advanced degree and above	−2.82	[−6.33, 0.70]	0.12
	Maternal age at delivery	−0.10	[−0.25, 0.05]	0.18
	Maternal marital status			
	Single	2.41	[0.32, 4.50]	0.02
	Child sex			
	Female	−2.28	[−3.72, −0.85]	0.002
	Child’s age at outcome	0.34	[−0.06, 0.75]	0.10

*Note:* ECHO cohort study site included as random intercept in all models. Unless noted (i.e. maternal ACEs), *b* values are unstandardized beta coefficients. ACEs = adverse childhood experiences; CI = confidence interval; ECHO = Environmental influences on Child Health Outcomes; PSS=Perceived Stress Scale.

a Maternal ACEs are standardized (*β*).

b Minimally adjusted model accounted for 11% of variance in child Total Problems score.

c Fully adjusted model accounted for 15% of variance in child Total Problems score.

To test whether the association between maternal prenatal stress and child outcomes was moderated by maternal history of ACEs (Aim 2), we ran an additional set of models, extending Model 2 to include an interaction term between ACEs and PSS (‘prenatalstressXACEs’). To test whether the association between maternal exposures and child outcomes differed by child sex (Aim 3), we included either ‘prenatalstressXsex’ or ‘ACEsXsex’ interaction terms in relevant models. Similarly, for Aim 4, given the wide age range of children in this study, we assessed whether associations between maternal stress exposures and child outcomes (as represented in Model 2) were moderated by child age by adding either a ‘prenatalstressXage’ or ‘ACEsXage’ interaction term in the models. Finally, building on Aim 2, given the mixed evidence that the effects of stress in utero might have differential mental health outcomes for offspring based on sex, a three-way interaction between ‘ACEsXprenatalstressXsex’ was also examined.

Secondary analyses were also conducted for the following conditions. First, ACE exposure was examined as a categorical variable to evaluate the dose–response association between maternal ACEs and child outcome to enhance clinical utility. Second, to further aid in interpretability with extant research, we assessed whether both maternal stress exposures were uniquely associated with child Internalizing Problems and Externalizing Problems by examining these domain-specific outcomes in separate models. Finally, given the variation in cohorts that provided data for this study, we performed a leave-one-out analysis. All analyses were completed in R version 4.1.0 (R Foundation for Statistical Computing, Vienna, Austria).

## Results

Demographic characteristics are presented in Table 1. Supplementary Figure S2 illustrates the geographic location of participants. Overall, the demographic characteristics across subsamples were similar, and subsamples were sociodemographically diverse. Approximately 58–78% of participants self-identified as White, 10–19% as Black, 3–9% as Asian, 3–5% as multiple races, 2–8% as another racial category, 1% or fewer as American Indian/Alaska and Native Hawaiian or other Pacific Islander, and 19–39% of participants self-identified as Hispanic. About half of the parents (48–60%) reported having a bachelor’s or advanced degree, and 19–23% reported having a high school degree or less. Combined annual household income was variable: 23–26% of families earned <$30,000, and 11–13% earned ≥$200,000. Consistent with global prevalence estimates (Madigan et al., 2023), ~64% of parents reported experiencing at least one ACE; almost 30% reported experiencing three or more ACEs. Child ages ranged from 1.5 to 18 years (mean age = 4.9–8.6 years across the three subsamples; see Supplementary Figure S3).

Correlations between key variables within each subsample can be found in Supplementary Tables S1A–S1C. In the ACEs + PSS subsample, maternal ACEs and prenatal stress were weakly correlated with each other (*r* = 0.22), thus limiting concerns for multicollinearity.

### Maternal stress exposures and child mental health problems

Maternal ACEs and prenatal stress were independently associated with child mental health problems (Aim 1), with findings consistent across Total Problems (Tables 2–4 and Supplementary Tables S2–S4), as well as in secondary analyses separating Externalizing Problems (Supplementary Tables S5–S7) and Internalizing Problems (Supplementary Tables S8–S10), such that exposure to more ACEs and prenatal stress were each associated with higher levels of child psychopathology. In the primary model (Model 2), maternal ACE exposure was significantly positively associated with child Total Problems (*B* = 2.53 [95% confidence interval [CI]: 2.09, 2.96], *p* < 0.0001; Table 2). Similarly, maternal prenatal stress was significantly positively associated with child Total Problems (*B* = 2.36 [95% CI: 2.03, 2.68], *p* < 0.0001; Table 3). For the subsample with data on maternal ACEs and prenatal stress, maternal ACEs (*B* = 1.72, 95% CI: [0.96, 2.48], *p* < 0.0001) and prenatal stress (*B* = 2.05, 95% CI: [1.29, 2.80], *p* < 0.0001) were independently and positively associated with child Total Problems (Table 4, Model 2). Supplementary Figure S4 provides an illustration of the dose–response relation between maternal ACEs and child Total Problems, adjusting for prenatal stress. Notably, associations between maternal ACEs and prenatal stress with child outcomes remained significant in extended models that adjusted for covariates considered ‘on the mechanistic path’ (e.g. maternal depressive symptoms; Supplementary Tables S2–S4, Model 3).

**Figure 1.**
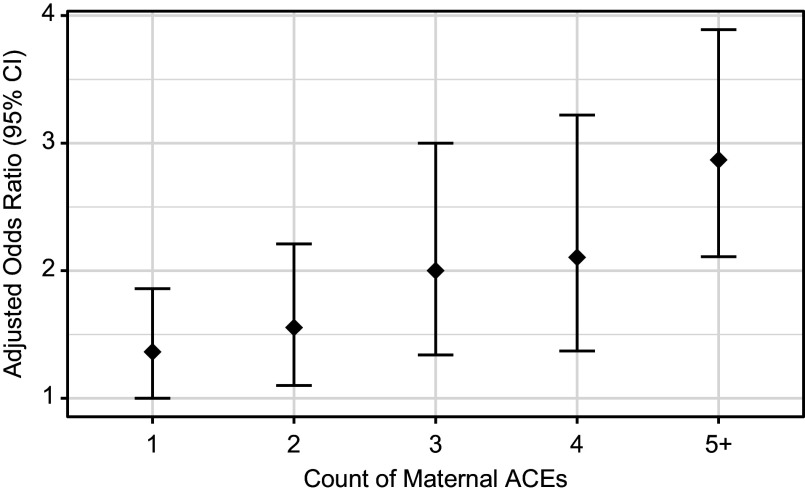
Effects of maternal adverse childhood experiences (ACEs) on odds of child mental health problems above the borderline threshold. Reference group = 0 ACEs. Findings are based on covariates in the fully adjusted model (Model 2) using ACEs as a categorical variable.

In our secondary analyses, we first assessed the relation between maternal stress exposures and the odds of a child scoring in the borderline or clinical problems range for Total Problems. For each additional maternal ACE, there was a 22% higher odds of a child having a Total Problems score above the borderline threshold (adjusted odds ratio [aOR] = 1.22, 95% CI: [1.16, 1.29], *p* < 0.0001). Similarly, for a 1−SD increase (10 points) in the maternal prenatal PSS T-score, there was a 4% higher odds of a child having a Total Problems score above the borderline threshold (aOR = 1.04, 95% CI: [1.03, 1.05], *p* < 0.0001). Moreover, in mutually adjusted models that included maternal prenatal stress, a one-unit increase in maternal ACEs was associated with a 17% higher odds of a child having a Total Problems score above the borderline threshold (Supplementary Table S11). Figure 1 provides an illustration of the dose–response relation between maternal ACEs and the dichotomous child outcome based on a fully adjusted logistic regression model. Next, we assessed associations between both maternal stress exposures and child Externalizing or Internalizing Problems in separate models in order to better understand potential domain specificity. Overall, we observed similar results across those models (Supplementary Tables S1–S13). Finally, in sensitivity analyses for our primary aim, leave-one-out analyses indicated that results were robust to individual cohorts being removed from the sample.

### Tests of moderation

For Aim 2, we found no evidence that maternal history of ACEs moderated the association between prenatal stress and child Total Problems (Model 2d; Supplement 1, Supplementary Table S4), Externalizing Problems (Supplementary Table S7), or Internalizing Problems (Supplementary Table S10). Regarding Aims 3 and 4, we did not find significant interactions with child sex (Model 2b; Supplement 1, Supplementary Table S2–S4) or child age (Model 2c; Supplement 1, Supplementary Tables S2–S4). Finally, our test of a three-way interaction among maternal ACEs, maternal prenatal stress, and child sex in the prediction of child Total Problems was not statistically significant (*B* = 1.05, 95% CI: [−0.52, 2.62], *p* = 0.19).

## Discussion

A substantial body of research has documented the mental health impact of ACEs and stress across the lifespan. A smaller but growing body of work highlights the intergenerational impact of maternal ACEs and pregnancy stress on offspring mental health. Few studies have tested the unique and potentially interactive contribution of maternal adversity experienced during childhood *and* pregnancy on offspring mental health outcomes. This large-scale examination leveraged a sociodemographically and geographically diverse sample of over 30 ECHO sites across 21 US states and territories. Consistent with DOHaD and prenatal programming theoretical frameworks and empirical evidence (see Bush, 2024, for a review), we found that maternal exposure to stress during two key sensitive periods in the mother’s own development – childhood and pregnancy – were independently associated with offspring mental health problems. Importantly, these two maternal stress exposures were only modestly correlated, indicating that they represent unique constructs. Notably, these associations were found in a sample of children across a broad age range, and they remained robust even after accounting for important factors, such as maternal postnatal depression and major social drivers of health (e.g. family socioeconomic position) that may lie on the mechanistic path or serve as confounders.

Our findings replicate, advance, and expand extant evidence for the potential etiological role maternal historical stress exposures play in the development of her children’s psychopathology, suggesting that maternal social experiences during key sensitive periods of their own development can leave a cumulative, lasting impact on offspring. Indeed, one pathway by which maternal ACEs could influence child mental health is through prenatal stress effects (e.g. altered maternal biological functioning and/or mental health), including during pregnancy. By examining both maternal ACEs and prenatal stress and finding independent effects, this analysis suggests that maternal ACEs are associated with offspring mental health above and beyond their potential mediating effects through perceived stress levels in pregnancy. Notably, ACE measures combine exposure types across a variety of childhood traumas (and do not account for timing, chronicity, or severity), so it is useful to remember that specific exposures likely have distinct effects on individual outcomes (e.g. brain development, stress reactivity, and mental health) (Jiang, Li, Jin, & Liang, 2025; Narayan, Lieberman, & Masten, 2021; Negriff, 2020) and intergenerational processes – contributing to multifinality for offspring mental health.

Further, both maternal exposures – independently and in mutually-adjusted models – were associated with a greater odds of children having clinically significant mental health problems. However, contrasting a prior finding that maternal childhood adversity amplified the effects of pregnancy stress on young children’s mental health problems (Bush et al., 2023), we did not find evidence that maternal ACE exposure moderated those associations, although our subsample of participants with data on both stress exposures was notably smaller than our samples with data on either exposure. Although our primary focus was on overall child psychopathology, our secondary analyses indicated that maternal stress exposures were associated with greater child risk in both of the two broad domains of child psychopathology – externalizing *and* internalizing problems. Our findings extend evidence beyond that from previous studies that have found some of the associations tested here – in either outcome domain – but not tested in the same sample (Noroña-Zhou et al., 2023; Tung et al., 2023). Findings align with the two known studies that previously examined both dimensions of child psychopathology in relation to maternal exposure to childhood and pregnancy stress in smaller, less diverse samples, suggesting maternal histories of stress affect children across a range of dimensions of psychopathology – in line with multifinality principles (Cicchetti & Rogosch, 1996). Maternal stress during pregnancy has been documented to affect the mother’s biology and behavior in a manner that changes the gestational milieu and alters fetal neurological development and subsequent psychopathology risk (Entringer, Buss, & Wadhwa, 2015; Monk et al., 2019). Although evidence is more sparse given this area is a newer focus of investigation, maternal adversities during the mother’s own childhood are understood to affect her biology in a manner that is potentially sustained through her life course, affecting her own pubertal development and pregnancy functioning (Aschbacher et al., 2021; Bush, 2024; Mamun et al., 2023; McDonald et al., 2019) and subsequently her offspring’s health. Although histories of adversity can also affect postnatal rearing behaviors and environment, animal models and findings adjusted for postnatal factors suggest that some of the risk for child psychopathology is transmitted in utero.

Our findings advance the evidence in other important ways, as the outcome timepoint in this study encompassed a broad range of child ages (including over 600 adolescents). Importantly, no variation in associations by child age were observed, enhancing our understanding of the potential impact of these maternal exposures across both childhood and adolescence, suggesting that effects extend throughout child development (Tung et al., 2023). This consistency in associations for both maternal exposures across children at differing developmental stages is particularly notable given the increasingly large role that broader environmental factors (e.g. school, peer relations, extracurricular influences, and social media) play in shaping adolescent outcomes (Cooke et al., 2022; Giletta et al., 2021; Messena & Everri, 2023; Pinquart, 2017; Yue & Rich, 2023), suggesting these influences can be longstanding.

Despite the study being well-powered to assess interactions, we found no evidence that associations between maternal ACEs or pregnancy stress and offspring mental health differed by child sex. This lack of moderation aligns with recent evidence in large, diverse samples and in meta-analyses (Bush et al., 2023; Noroña-Zhou et al., 2023; Tung et al., 2023). These results were also strengthened by leave-one-out analyses showing that the findings were not driven by particular cohorts.

Using a large sample broadly representing families across the United States, this study contributes to a growing body of knowledge regarding the intergenerational impact of maternal stress exposures during two key sensitive periods of the mother’s life on offspring mental health. Research on child and adolescent health and development has increasingly focused on the preconception or prenatal environment; however, our study highlights the importance of collectively considering both of these sensitive periods in a biological parent’s life when examining the intergenerational impact on offspring mental health. Such exposures can influence offspring health through maternal biological changes in the preconception and prenatal periods (e.g. hormones and immune function) and through psychosocial factors postnatally (e.g. maternal mental health, which is a strong risk factor for child mental health). These findings, therefore, underscore the importance of detection, prevention, and early intervention efforts (indeed, even before pregnancy) to promote the well-being of pregnant individuals and their children.

This study has several strengths, including the use of a large, sociodemographically and geographically diverse sample and the inclusion of two distinct and important intergenerational maternal risk factors for offspring mental health. We also assessed the association between these maternal stress exposures and internalizing *and* externalizing mental health problems in children and adolescents, whereas much extant research has focused on only one domain of child psychopathology or only in infancy/childhood. Some limitations are noteworthy. First, measurement of maternal exposures and child outcomes, although evaluated using well-established measures, was based primarily on biological mothers’ report. Future studies would benefit from including other sources for both risk exposures (i.e. paternal ACEs) and child behavior (child self-report or teacher report), especially since these latter informants become more important during later childhood and adolescence. Second, although our study included a broad range of child ages, our sample of adolescents was relatively small, and future studies would benefit from assessing these associations in large post-puberty samples to better unpack the consistency of effects across development. Further, future longitudinal studies with repeated outcome assessments are needed to determine whether these intergenerational effects of maternal stress are stronger near pregnancy or become amplified (or attenuated) as offspring mental health problems unfold across adolescence. The present study also did not explore the effects of the timing of prenatal stressors on child outcomes (e.g. first vs. second or third trimester of pregnancy); future studies may benefit from examining such timing effects. Finally, this study focused on the intergenerational impacts of maternal ACEs and prenatal stress. Expansion in future studies to include maternal stress experiences postnatally would allow for the examination of more proximal measures of stress exposure and their associations with offspring mental health.

Although emerging evidence suggests that caregivers *can* help buffer the intergenerational transmission of maternal stress (Ahmad et al., 2022; Jones-Mason et al., 2023; Ma et al., 2022), future research should continue to investigate the buffering potential of parenting (especially very understudied father factors) and other malleable psychosocial factors across both sensitive periods of maternal risk. The assessment of resilience-promoting factors is critical for the prevention and mitigation of mental health problems in children and adolescents.

The findings from this study provide support for the intergenerational impact of maternal ACEs and prenatal stress on child and adolescent mental health. These findings suggest that early screening and prevention efforts, even before childbirth, would have a two-generational benefit. Additionally, given that these intergenerational associations appear through adolescence, early childhood interventions may provide longer-term benefits for offspring. Providing early support to parents and families is key in prevention efforts, and although this compelling body of science has sparked some recent policy shifts – including specific state’s efforts screening for ACEs to identify children and families who may benefit from early intervention in primary care contexts (California Department of Health Care Services, n.d.) – additional policy efforts are sorely needed. Increasing screening for ACE exposure and current social needs in pregnant populations and enhancing access to prenatal psychosocial interventions that reduce maternal distress (e.g. Epel et al., 2019; Roubinov, Epel, et al., 2022) may promote two-generation mental health (Bush, 2024; Noroña-Zhou et al., 2022). In addition to supporting individuals, it is essential to restructure systems that are responsible for adversity exposure (Chater & Loewenstein, 2023). As summarized in the Prenatal-to-3 Policy Clearinghouse (https://pn3policy.org/pn-3-state-policy-clearinghouse/), numerous effective interventions exist – many of which target mechanisms underlying adversity exposure – to support the health of families with young children. For example, expanding paid family and medical leave and increasing access to childcare subsidies may reduce maternal stress and promote two-generation health. Given the global burden of disease associated with mental health problems (Vigo, Thornicroft, & Atun, 2016; Whiteford et al., 2013), such supports and early intervention efforts should be a public health priority.

## Supporting information

10.1017/S0033291725103127.sm001 Ahmad et al. supplementary material Ahmad et al. supplementary material

## Data Availability

Select de-identified data from the ECHO Program are available through NICHD’s Data and Specimen Hub (DASH). Information on study data not available on DASH, such as some Indigenous datasets, can be found on the ECHO study DASH webpage.

## Supplementary material

The supplementary material for this article can be found at http://doi.org/10.1017/S0033291725103127.

## Access to data and data sharing

Marie L. Churchill attests that she had full access to the data and takes responsibility for the analysis.

## Abbreviations

ACE: adverse childhood experience; ADHD: attention-deficit/hyperactivity disorder; aOR: adjusted odds ratio; CI: confidence interval; DASH: Data and Specimen Hub; DCRI: Duke Clinical Research Institute; DOHaD: Development Origins of Health and Disease; ECHO: Environmental influences on Child Health Outcomes; HBCD: HEALthy Brain and Child Development; NIH: National Institutes of Health; OSMB: Observational Study Monitoring Board; OTIS: Organization of Teratology Information Specialists; PROMIS: Patient-Reported Outcomes Measurement Information System; PSS: Perceived Stress Scale; SD: standard deviation; SDQ: Strengths and Difficulties Questionnaire.

## A. Appendix

**Table A1.** ECHO collaborator

First name and middle initial(s)	Last name	Suffix (e.g. Jr, III)	Academic degrees	Department	Institution	Location (city, state/province, country)	Role or contribution	ECHO Cohort Study site or core name and grant number	Email address
P Brian	Smith		MD, MPH, MHS	Division of Neonatology, Department of Pediatrics	Duke Clinical Research Institute, Duke University School of Medicine	Durham, North Carolina, USA	ECHO Coordinating Center Principal Investigator	U2COD023375 (Coordinating Center)	brian.smith@duke.edu
L Kristin	Newby		MD, MHS	Division of Cardiology, Department of Medicine	Duke Clinical Research Institute, Duke University School of Medicine	Durham, North Carolina, USA	ECHO Coordinating Center Principal Investigator	U2COD023375 (Coordinating Center)	kristin.newby@duke.edu
Linda	Adair		PhD	Department of Nutrition	Gillings School of Global Public Health, University of North Carolina at Chapel Hill	Chapel Hill, North Carolina, USA	ECHO Coordinating Center Principal Investigator	U2COD023375 (Coordinating Center)	linda_adair@unc.edu
Lisa P.	Jacobson		ScD	Department of Epidemiology	Johns Hopkins University, Bloomberg School of Public Health	Baltimore, Maryland, USA	ECHO Data Analysis Center Principal Investigator	U24OD023382 (Data Analysis Center)	ljacobs1@jhu.edu
Diane	Catellier		DrPH	N/A	Research Triangle Institute	Research Triangle Park, North Carolina, USA	ECHO Data Analysis Center Principal Investigator	U24OD023382 (Data Analysis Center)	dcatellier@rti.org
Monica	McGrath		ScD	Department of Epidemiology	Johns Hopkins University, Bloomberg School of Public Health	Baltimore, Maryland, USA	ECHO Johns Hopkins University Data Analysis Center Director Co-Investigator	U24OD023382 (Data Analysis Center)	mmcgrat4@jhu.edu
Christian	Douglas		DrPH	N/A	Research Triangle Institute	Research Triangle Park, North Carolina, USA	ECHO RTI Data Analysis Center Director Co-Investigator	U24OD023382 (Data Analysis Center)	christiand@rti.org
Priya	Duggal		PhD	Department of Epidemiology	Johns Hopkins University, Bloomberg School of Public Health	Baltimore, Maryland, USA	ECHO Data Analysis Center Genetics Methods Lead, Co-Investigator	U24OD023382 (Data Analysis Center)	pduggal@jhu.edu
Emily	Knapp		PhD	Department of Epidemiology	Johns Hopkins University, Bloomberg School of Public Health	Baltimore, Maryland, USA	ECHO Data Analysis Center Co-Investigator	U24OD023382 (Data Analysis Center)	eknapp2@jhu.edu
Amii	Kress		PhD	Department of Epidemiology	Johns Hopkins University, Bloomberg School of Public Health	Baltimore, Maryland, USA	ECHO Data Analysis Center General Methods Co-Investigator	U24OD023382 (Data Analysis Center)	akress1@jhu.edu
Courtney K.	Blackwell		PhD	Department of Medical Social Sciences	Feinberg School of Medicine, Northwestern University	Chicago, Illinois, USA	Measurement Core Co-Investigator	U24OD023319 with co-funding from the Office of Behavioral and Social Science Research (Measurement Core)	Ckblackwell@northwestern.edu
Maxwell A.	Mansolf		PhD	Department of Medical Social Sciences	Feinberg School of Medicine, Northwestern University	Chicago, Illinois, USA	Measurement Core Co-Investigator	U24OD023319 with co-funding from the Office of Behavioral and Social Science Research (Measurement Core)	maxwell.mansolf@northwestern.edu
Jin-Shei	Lai		PhD	Department of Medical Social Sciences	Feinberg School of Medicine, Northwestern University	Chicago, Illinois, USA	Measurement Core Co-Investigator	U24OD023319 with co-funding from the Office of Behavioral and Social Science Research (Measurement Core)	js-lai@northwestern.edu
Emily	Ho		PhD	Department of Medical Social Sciences	Feinberg School of Medicine, Northwestern University	Chicago, Illinois, USA	Measurement Core Co-Investigator	U24OD023319 with co-funding from the Office of Behavioral and Social Science Research (Measurement Core)	emily-ho@northwestern.edu
David	Cella		PhD	Department of Medical Social Sciences	Feinberg School of Medicine, Northwestern University	Chicago, Illinois, USA	Measurement Core Principal Investigator	U24OD023319 with co-funding from the Office of Behavioral and Social Science Research (Measurement Core)	d-cella@northwestern.edu
Richard	Gershon		PhD	Department of Medical Social Sciences	Feinberg School of Medicine, Northwestern University	Chicago, Illinois, USA	Measurement Core Principal Investigator	U24OD023319 with co-funding from the Office of Behavioral and Social Science Research (Measurement Core)	gershon@northwestern.edu
Michelle L.	Macy		MD	Department of Pediatrics	Feinberg School of Medicine, Northwestern University and Ann & Robert H. Lurie Children’s Hospital of Chicago	Chicago, Illinois, USA	Measurement Core Co-Investigator	U24OD023319 with co-funding from the Office of Behavioral and Social Science Research (Measurement Core)	mmacy@luriechildrens.org
Suman R.	Das		PhD	Division of Infectious Diseases, Department of Medicine	Vanderbilt University Medical Center	Nashville, Tennessee, USA	ECHO Laboratory Core Principal Investigator	U24OD035523 (Lab Core)	suman.r.das@vumc.org
Jane E.	Freedman		MD	Division of Cardiovascular Medicine, Department of Medicine	Vanderbilt University Medical Center	Nashville, Tennessee, USA	ECHO Laboratory Core Principal Investigator	U24OD035523 (Lab Core)	jane.freedman@vumc.org
Simon A.	Mallal		MBBS	Division of Infectious Diseases, Department of Medicine	Vanderbilt University Medical Center	Nashville, Tennessee, USA	ECHO Laboratory Core Principal Investigator	U24OD035523 (Lab Core)	s.mallal@vumc.org
John A.	McLean		PhD	Department of Chemistry	Vanderbilt University	Nashville, Tennessee, USA	ECHO Laboratory Core Principal Investigator	U24OD035523 (Lab Core)	john.a.mclean@vanderbilt.edu
Ravi V.	Shah		MD	Division of Cardiovascular Medicine, Department of Medicine	Vanderbilt University Medical Center	Nashville, Tennessee, USA	ECHO Laboratory Core Principal Investigator	U24OD035523 (Lab Core)	ravi.shah@vumc.org
Meghan H.	Shilts		MHS	Division of Infectious Diseases, Department of Medicine	Vanderbilt University Medical Center	Nashville, Tennessee, USA	ECHO Laboratory Core Principal Investigator Admin Designee	U24OD035523 (Lab Core)	meghan.h.shilts@vumc.org
Akram N.	Alshawabkeh		PhD	College of Engineering	Northeastern University	Boston, Massachusetts, USA	ECHO Cohort Study Site Principal Investigator	UG3/UH3OD023251 (Akram Alshawabkeh)	a.alshawabkeh@northeastern.edu
Jose F.	Cordero		MD	College of Public Health, Department of Epidemiology & Biostatistics	University of Georgia	Athens, Georgia, USA	ECHO Cohort Study Site Co-Director	UG3/UH3OD023251 (Akram Alshawabkeh)	jcordero@uga.edu
John	Meeker		ScD	Environmental Health Sciences, School of Public Health	University of Michigan	Ann Arbor, Michigan, USA	ECHO Cohort Study Site Co-Director	UG3/UH3OD023251 (Akram Alshawabkeh)	meekerj@umich.edu
Leonardo	Trasande		MD, MPP	Departments of Pediatrics and Population Health	NYU Grossman School of Medicine	New York, New York, USA	ECHO Cohort Study Site Principal Investigator	UG3/UH3OD023305 (Leonardo Trasande)	leonardo.trasande@nyulangone.org
Carlos A.	Camargo	Jr.	MD, DrPH	Department of Emergency Medicine	Massachusetts General Hospital, Harvard Medical School	Boston, Massachusetts, USA	ECHO Cohort Study Site Principal Investigator	UG3/UH3OD023253 (Carlos Camargo)	ccamargo@mgb.org
Kohei	Hasegawa		MD, PhD	Department of Emergency Medicine	Massachusetts General Hospital, Harvard Medical School	Boston, Massachusetts, USA	ECHO Cohort Study Site Co-Investigator	UG3/UH3OD023253 (Carlos Camargo)	khasegawa@mgh.harvard.edu
Zhaozhong	Zhu		ScD	Department of Emergency Medicine	Massachusetts General Hospital, Harvard Medical School	Boston, Massachusetts, USA	ECHO Cohort Study Site Co-Investigator	UG3/UH3OD023253 (Carlos Camargo)	zzhu5@mgh.harvard.edu
Ashley F.	Sullivan		MS, MPH	Department of Emergency Medicine	Massachusetts General Hospital, Harvard Medical School	Boston, Massachusetts, USA	ECHO Cohort Study Site Award Project Director	UG3/UH3OD023253 (Carlos Camargo)	afsullivan@mgb.org
Dana	Dabelea		MD, PhD	Lifecourse Epidemiology of Adiposity and Diabetes (LEAD) Center	University of Colorado Anschutz Medical Campus	Aurora, Colorado, USA	ECHO Cohort Study Site Principal Investigator	UG3/UH3OD023248 and UG3OD035526 (Dana Dabelea)	dana.dabelea@cuanschutz.edu
Wei	Perng		PhD, MPH	Lifecourse Epidemiology of Adiposity and Diabetes (LEAD) Center	University of Colorado Anschutz Medical Campus	Aurora, Colorado, USA	ECHO Cohort Study Site Principal Investigator	UG3/UH3OD023248 (Dana Dabelea)	wei.perng@cuanschutz.edu
Traci A.	Bekelman		PhD, MPH	Lifecourse Epidemiology of Adiposity and Diabetes (LEAD) Center	University of Colorado Anschutz Medical Campus	Aurora, Colorado, USA	ECHO Cohort Study Site Principal Investigator	UG3/UH3OD023248 (Dana Dabelea)	traci.bekelman@cuanschutz.edu
Greta	Wilkening		PhD, MPH	Lifecourse Epidemiology of Adiposity and Diabetes (LEAD) Center	University of Colorado Anschutz Medical Campus	Aurora, Colorado, USA	ECHO Cohort Study Site Co-Investigator	UG3/UH3OD023248 (Dana Dabelea)	Greta.Wilkening@childrenscolorado.org
Sheryl	Magzamen		PhD	Environmental and Radiological Health Sciences	Colorado School of Public Health, Colorado State University	Fort Collins, Colorado, USA	ECHO Cohort Study Site Co-Investigator	UG3OD035526 (Dana Dabelea)	Sheryl.Magzamen@colostate.edu
Brianna F.	Moore		PhD, MS	Lifecourse Epidemiology of Adiposity and Diabetes (LEAD) Center	University of Colorado Anschutz Medical Campus	Aurora, Colorado, USA	ECHO Cohort Study Site Principal Investigator	UG3OD035526 (Dana Dabelea)	brianna.f.moore@cuanschutz.edu
Anne P.	Starling		PhD	Epidemiology	University of North Carolina at Chapel Hill	Chapel Hill, North Carolina, USA	ECHO Cohort Study Site Principal Investigator	UG3OD035526 (Dana Dabelea)	anne.starling@unc.edu
Deborah J.	Rinehart		PhD	Center for Health Systems Research	Denver Health and Hospital Authority	Denver, Colorado, USA	ECHO Cohort Study Site Co-Investigator	UG3OD035526 (Dana Dabelea)	deborah.rinehart@dhha.org
Daphne	Koinis Mitchell		PhD	Department of Pediatrics	Rhode Island Hospital, The Alpert Medical School of Brown University	Providence, Rhode Island, USA	ECHO Cohort Study Site Principal Investigator	UG3/UH3OD023313 (Daphne Koinis Mitchell)	dkoinismitchell@lifespan.org
Viren	D’Sa		MD	Department of Pediatrics	Rhode Island Hospital, The Alpert Medical School of Brown University	Providence, Rhode Island, USA	ECHO Cohort Study Site Principal Investigator	UG3/UH3OD023313 (Daphne Koinis Mitchell)	viren_Dsa@brown.edu
Sean C.L.	Deoni		PhD	Division of Gender Equality, Maternal, Newborn & Child Health Discovery & Tools Team	Bill & Melinda Gates Foundation	Seattle, Washington, USA	ECHO Cohort Study Site Principal Investigator	UG3/UH3OD023313 (Daphne Koinis Mitchell)	Sean.Deoni@gatesfoundation.org
Hans-Georg	Mueller		PhD	Department of Statistics	University of California, Davis	Davis, California, USA	ECHO Cohort Study Site Co-Investigator	UG3/UH3OD023313 (Daphne Koinis Mitchell)	hgmueller@ucdavis.edu
Cristiane S.	Duarte		PhD, MPH	Division of Child and Adolescent Psychiatry	Columbia University - NYSPI	New York, New York, USA	ECHO Cohort Study Site Principal Investigator	UH3OD023328 (Cristiane Duarte)	Cristiane.Duarte@nyspi.columbia.edu
Catherine	Monk		PhD	Department of Obstetrics & Gynecology	Columbia University - NYSPI	New York, New York, USA	ECHO Cohort Study Site Principal Investigator	UH3OD023328 (Cristiane Duarte)	cem31@cumc.columbia.edu
Glorisa	Canino		PhD	Behavioral Sciences Research Institute	University of Puerto Rico, School of Medicine	Rio Piedras, Puerto Rico	ECHO Cohort Study Site Principal Investigator	UH3OD023328 (Cristiane Duarte)	glorisa.canino@upr.edu
Jonathan	Posner		MD	Child & Family Mental Health & Community Psychiatry Division	Duke University School of Medicine, Duke Psychiatry & Behavioral Sciences	Durham, North Carolina, USA	ECHO Cohort Study Site Principal Investigator	UH3OD023328 (Cristiane Duarte)	jonathan.posner@duke.edu
Tenneill	Murray		MPH	Division of Child and Adolescent Psychiatry	Columbia University - NYSPI	New York, New York, USA	ECHO Cohort Study Site Co-Director	UH3OD023328 (Cristiane Duarte)	tenneill.murray@nyspi.columbia.edu
Claudia	Lugo-Candelas		PhD	Division of Child and Adolescent Psychiatry	Columbia University - NYSPI	New York, New York, USA	ECHO Cohort Study Site Principal Investigator	UH3OD023328 (Cristiane Duarte)	claudia.lugo@nyspi.columbia.edu
Anne L.	Dunlop		MD, MPH	Department of Gynecology and Obstetrics	Emory University School of Medicine	Atlanta, Georgia, USA	ECHO Cohort Study Site Principal Investigator	UH3OD023318 (Anne Dunlop)	amlang@emory.edu
Patricia A.	Brennan		PhD	Department of Psychology	Emory University	Atlanta, Georgia, USA	ECHO Cohort Study Site Principal Investigator	UH3OD023318 (Anne Dunlop)	pbren01@emory.edu
Christine	Hockett		PhD	N/A; Department of Pediatrics	Avera Research Institute; University of South Dakota School of Medicine	Rapid City, South Dakota, USA; Sioux Falls, South Dakota, USA	ECHO Cohort Study Site Principal Investigator	UG3/UH3OD023279 (Amy Elliott)	christine.hockett@avera.org
Amy	Elliott		PhD	N/A; Department of Pediatrics	Avera Research Institute; University of South Dakota School of Medicine	Sioux Falls, South Dakota, USA	ECHO Cohort Study Site Principal Investigator	UG3/UH3OD023279 (Amy Elliott)	amy.elliott@avera.org
Assiamira	Ferrara		MD, PhD	Division of Research	Kaiser Permanente Northern California	Oakland, California, USA	ECHO Cohort Study Site Principal Investigator	UG3/UH3OD023289 (Assiamira Ferrara)	assiamira.ferrara@kp.org
Lisa A.	Croen		PhD	Division of Research	Kaiser Permanente Northern California	Oakland, California, USA	ECHO Cohort Study Site Principal Investigator	UG3/UH3OD023342 (Kristen Lyall), UG3/UH3OD023289 (Assiamira Ferrara)	Lisa.A.Croen@kp.org
Monique M.	Hedderson		PhD	Division of Research	Kaiser Permanente Northern California	Oakland, California, USA	ECHO Cohort Study Site Principal Investigator	UG3/UH3OD023289 (Assiamira Ferrara), UG3OD035540 (Monique Marie Hedderson)	Monique.M.Hedderson@kp.org
John	Ainsworth		PhD	Centre for Health Informatics	University of Manchester	Manchester, United Kingdom	ECHO Cohort Study Site Principal Investigator	UG3/UH3OD023282 (James Gern)	John.Ainsworth@manchester.ac.uk
Leonard B.	Bacharier		MD	Department of Pediatrics, Monroe Carell Jr Children’s Hospital at Vanderbilt	Vanderbilt University Medical Center	Nashville, Tennessee, USA	ECHO Cohort Study Site Principal Investigator	UG3/UH3OD023282 (James Gern)	leonard.bacharier@vumc.org
Casper G.	Bendixsen		PhD	National Farm Medicine Center	Marshfield Clinic Research Institute	Marshfield, Wisconsin, USA	ECHO Cohort Study Site Principal Investigator	UG3/UH3OD023282 (James Gern)	Bendixsen.casper@marshfieldresearch.org
James E.	Gern		MD	Department of Pediatrics	University of Wisconsin School of Medicine and Public Health	Madison, Wisconsin, USA	ECHO Cohort Study Site Principal Investigator	UG3/UH3OD023282 (James Gern), UG3OD035509 (Anne Marie Singh)	gern@medicine.wisc.edu
Diane R.	Gold		MD	The Channing Division of Network Medicine; Department of Medicine	Brigham and Women’s Hospital; Harvard Medical School	Boston, Massachusetts, USA	ECHO Cohort Study Site Principal Investigator	UG3/UH3OD023282 (James Gern)	redrg@channing.harvard.edu
Tina V.	Hartert		MD, MPH	Division of Pediatric Allergy, Immunology, and Pulmonary Medicine, Department of Medicine, Department of Pediatrics	Vanderbilt University Medical Center	Nashville, Tennessee, USA	ECHO Cohort Study Site Principal Investigator	UG3/UH3OD023282 (James Gern), UG3OD035516 and UG3OD035517 (Tina Hartert)	tina.hartert@vumc.org
Daniel J.	Jackson		MD	Department of Pediatrics	University of Wisconsin School of Medicine and Public Health	Madison, Wisconsin, USA	ECHO Cohort Study Site Principal Investigator	UG3/UH3OD023282 (James Gern)	djj@medicine.wisc.edu
Christine C.	Johnson		PhD	Department of Public Health Sciences	Henry Ford Health	Detroit, Michigan, USA	ECHO Cohort Study Site Principal Investigator	UG3/UH3OD023282 (James Gern), UG3OD035518 (Jennifer Straughen)	CJOHNSO1@hfhs.org
Christine L.M.	Joseph		PhD	Department of Public Health Sciences	Henry Ford Health	Detroit, Michigan, USA	ECHO Cohort Study Site Principal Investigator	UG3/UH3OD023282 (James Gern)	cjoseph1@hfhs.org
Meyer	Kattan		MD	Department of Pediatrics	Columbia University Medical Center	New York, New York, USA	ECHO Cohort Study Site Principal Investigator	UG3/UH3OD023282 (James Gern)	mk2833@cumc.columbia.edu
Gurjit K.	Khurana Hershey		MD, PhD	Division of Asthma Research	Cincinnati Children’s Hospital Medical Center	Cincinnati, Ohio, USA	ECHO Cohort Study Site Principal Investigator	UG3/UH3OD023282 (James Gern)	gurjit.hershey@cchmc.org
Robert F.	Lemanske, Jr.		MD	Department of Pediatrics	University of Wisconsin School of Medicine and Public Health	Madison, Wisconsin, USA	ECHO Cohort Study Site Principal Investigator	UG3/UH3OD023282 (James Gern)	lemanske@wisc.edu
Susan V.	Lynch		PhD	Department of Medicine	University of California	San Francisco, California, USA	ECHO Cohort Study Site Principal Investigator	UG3/UH3OD023282 (James Gern)	Susan.Lynch@ucsf.edu
Rachel L.	Miller		MD	Department of Medicine; Division of Clinical Immunology	Icahn School of Medicine at Mount Sinai	New York, New York, USA	ECHO Cohort Study Site Principal Investigator	UG3/UH3OD023282 (James Gern), UG3/UH3OD023290 (Julie Herbstman)	Rachel.miller2@mssm.edu
George T.	O’Connor		MD	Department of Pediatrics	Boston University School of Medicine	Boston, Massachusetts, USA	ECHO Cohort Study Site Principal Investigator	UG3/UH3OD023282 (James Gern)	goconnor@bu.edu
Carole	Ober		PhD	Department of Human Genetics	University of Chicago	Chicago, Illinois, USA	ECHO Cohort Study Site Principal Investigator	UG3/UH3OD023282 (James Gern), UG3OD035509 (Anne Marie Singh)	c-ober@genetics.uchicago.edu
Dennis	Ownby		MD	Department of Public Health Sciences	Henry Ford Health	Detroit, Michigan, USA	ECHO Cohort Study Site Principal Investigator	UG3/UH3OD023282 (James Gern)	downby@augusta.edu
Katherine	Rivera-Spoljaric		MD	Department of Pediatrics	Washington University School of Medicine	St Louis, Missouri, USA	ECHO Cohort Study Site Principal Investigator	UG3/UH3OD023282 (James Gern), UG3OD035521 (Katherine Rivera-Spoljaric)	rivera_k@wustl.edu
Patrick H.	Ryan		PhD	Department of Pediatrics and College of Medicine; Division of Biostatistics and Epidemiology	University of Cincinnati	Cincinnati, Ohio, USA	ECHO Cohort Study Site Principal Investigator	UG3/UH3OD023282 (James Gern), UG3OD035509 (Anne Marie Singh)	patrick.ryan@cchmc.org
Christine M.	Seroogy		MD	Department of Pediatrics	University of Wisconsin School of Medicine and Public Health	Madison, Wisconsin, USA	ECHO Cohort Study Site Principal Investigator	UG3/UH3OD023282 (James Gern)	cmseroogy@wisc.edu
Anne Marie	Singh		MD	Department of Pediatrics	University of Wisconsin School of Medicine and Public Health	Madison, Wisconsin, USA	ECHO Cohort Study Site Principal Investigator	UG3/UH3OD023282 (James Gern), UG3OD035509 (Anne Marie Singh)	amsingh@wisc.edu
Robert A.	Wood		MD	Department of Pediatrics	Johns Hopkins University School of Medicine	Baltimore, Maryland, USA	ECHO Cohort Study Site Principal Investigator	UG3/UH3OD023282 (James Gern)	rwood@jhmi.edu
Edward M.	Zoratti		MD	Division of Allergy and Clinical Immunology	Henry Ford Health	Detroit, Michigan, USA	ECHO Cohort Study Site Principal Investigator	UG3/UH3OD023282 (James Gern), UG3OD035518 (Jennifer Straughen)	ezoratt1@hfhs.org
Rima	Habre		ScD, MSc	Department of Population and Public Health Sciences	University of Southern California	Los Angeles, California, USA	ECHO Cohort Study Site Principal Investigator	UH3OD023287 (Carrie Breton)	habre@usc.edu
Shohreh	Farzan		PhD	Department of Population and Public Health Sciences	University of Southern California	Los Angeles, California, USA	ECHO Cohort Study Site Principal Investigator	UH3OD023287 (Carrie Breton)	sffarzan@usc.edu
Frank D.	Gilliland		MD, MPH, PhD	Department of Population and Public Health Sciences	University of Southern California	Los Angeles, California, USA	ECHO Cohort Study Site Principal Investigator	UH3OD023287 (Carrie Breton)	gillilan@usc.edu
Irva	Hertz-Picciotto		PhD	MIND Institute and Department of Public Health Sciences	University of California, Davis	Davis, California, USA	ECHO Cohort Study Site Principal Investigator	UG3/UH3OD023365 (Irva Hertz-Picciotto), UG3OD035550 (Rebecca Schmidt)	iher@ucdavis.edu
Deborah H.	Bennett		PhD	Department of Public Health Sciences	University of California, Davis	Davis, California, USA	ECHO Cohort Study Site Principal Investigator	UG3/UH3OD023365 (Irva Hertz-Picciotto), UG3OD035550 (Rebecca Schmidt)	dhbennett@ucdavis.edu
Julie B.	Schweitzer		PhD	Department of Psychiatry and Behavioral Science and the MIND Institute	University of California, Davis	Davis, California, USA	ECHO Cohort Study Site Principal Investigator	UG3/UH3OD023365 (Irva Hertz-Picciotto)	jschweitzer@ucdavis.edu
Rebecca J.	Schmidt		PhD	MIND Institute and Department of Public Health Sciences	University of California, Davis	Davis, California, USA	ECHO Cohort Study Site Principal Investigator	UG3/UH3OD023365 (Irva Hertz-Picciotto), UG3/UH3OD023342 (Kristen Lyall), UG3OD035550 (Rebecca Schmidt)	rjschmidt@ucdavis.edu
Janine M.	LaSalle		PhD	Medical Microbiology and Immunology; MIND Institute	University of California, Davis	Davis, California, USA	ECHO Cohort Study Site Co-Investigator	UG3/UH3OD023365 (Irva Hertz-Picciotto), UG3OD035550 (Rebecca Schmidt)	jmlasalle@ucdavis.edu
Alison E.	Hipwell		PhD, ClinPsyD	Psychiatry and Psychology	University of Pittsburgh	Pittsburgh, Pennsylvania, USA	ECHO Cohort Study Site Principal Investigator	UG3/UH3OD023244 (Alison Hipwell)	hipwae@upmc.edu
Catherine J.	Karr		MD, MS, PhD	Department of Pediatrics, School of Medicine; Department of Environmental and Occupational Health Sciences; School of Public Health	University of Washington	Seattle, Washington, USA	ECHO Cohort Study Site Principal Investigator	UH3OD023271 and UG3OD035528 (Catherine Karr)	ckarr@uw.edu
Nicole R.	Bush		PhD	Department of Psychiatry and Behavioral Sciences and Department of Pediatrics, School of Medicine	University of California, San Francisco	San Francisco, California, USA	ECHO Cohort Study Site Principal Investigator	UH3OD023271 (Catherine Karr), UG3OD035519 (Qi Zhao)	nicole.bush@ucsf.edu
Kaja Z.	LeWinn		ScD	Department of Psychiatry and Behavioral Sciences, School of Medicine	University of California, San Francisco	San Francisco, California, USA	ECHO Cohort Study Site Principal Investigator	UH3OD023271 (Catherine Karr), UG3OD035519 (Qi Zhao)	kaja.lewinn@ucsf.edu
Sheela	Sathyanarayana		MD, MPH	Department of Pediatrics, School of Medicine; Department of Environmental and Occupational Health Sciences, School of Public Health	University of Washington and Seattle Children’s Research Institute	Seattle, Washington, USA	ECHO Cohort Study Site Principal Investigator	UH3OD023271 (Catherine Karr), UG3OD035508 (Sheela Sathyanarayana)	sheela.sathyanarayana@seattlechildrens.org
Qi	Zhao		MD, PhD	Department of Preventive Medicine	University of Tennessee Health Science Center	Memphis, Tennessee, USA	ECHO Cohort Study Site Principal Investigator	UH3OD023271 (Catherine Karr), UG3OD035519 (Qi Zhao)	qzhao11@uthsc.edu
Frances	Tylavsky		DrPH, MS	Department of Preventive Medicine	University of Tennessee Health Science Center	Memphis, Tennessee, USA	ECHO Cohort Study Site Principal Investigator	UH3OD023271 (Catherine Karr)	ftylavsk@uthsc.edu
Kecia N.	Carroll		MD, MPH	Department of Pediatrics, Department of Environmental Medicine & Public Health	Icahn School of Medicine at Mount Sinai	New York, New York, USA	ECHO Cohort Study Site Principal Investigator	UH3OD023271 (Catherine Karr), UG3/UH3OD023337 (Rosalind Wright)	kecia.carroll@mssm.edu
Christine T.	Loftus		MS MPH PhD	Department of Environmental and Occupational Health Sciences; School of Public Health	University of Washington	Seattle, Washington, USA	ECHO Cohort Study Site Principal Investigator	UH3OD023271 (Catherine Karr)	cloftus@uw.edu
Leslie D.	Leve		PhD	Department of Counseling Psychology and Human Services & Prevention Science Institute	University of Oregon	Eugene, Oregon, USA	ECHO Cohort Study Site Principal Investigator	UG3/UH3OD023389 (Leslie Leve)	leve@uoregon.edu
Jody M.	Ganiban		PhD	Department of Psychological and Behavioral Sciences	George Washington University	Washington, DC, USA	ECHO Cohort Study Site Principal Investigator	UG3/UH3OD023389 (Leslie Leve)	ganiban@gwu.edu
Jenae M.	Neiderhiser		PhD	Department of Psychology	Penn State University	University Park, Pennsylvania, USA	ECHO Cohort Study Site Principal Investigator	UG3/UH3OD023389 (Leslie Leve)	jenaemn@psu.edu
Scott T.	Weiss		MD	Channing Division of Network Medicine, Department of Medicine	Brigham and Women’s Hospital and Harvard Medical School	Boston, Massachusetts, USA	ECHO Cohort Study Site Principal Investigator	UH3OD023268 (Scott Weiss)	scott.weiss@channing.harvard.edu
Augusto A.	Litonjua		MD	Pediatric Pulmonary Division, Department of Pediatrics	Golisano Children’s Hospital, University of Rochester	Rochester, New York, USA	ECHO Cohort Study Site Principal Investigator	UH3OD023268 (Scott Weiss)	augusto_litonjua@urmc.rochester.edu
Cindy T.	McEvoy		MD, MCR	Division of Neonatology, Department of Pediatrics	Oregon Health & Science University	Portland, Oregon, USA	ECHO Cohort Study Site Principal Investigator	UG3/UH3OD023288 (Cynthia McEvoy)	mcevoyc@ohsu.edu
Eliot R.	Spindel		MD, PhD	Division of Neuroscience	Oregon National Primate Research Center	Beaverton, Oregon, USA	ECHO Cohort Study Site Principal Investigator	UG3/UH3OD023288 (Cynthia McEvoy)	spindele@ohsu.edu
Robert S.	Tepper		MD, PhD	Division of Pediatric Pulmonology, Department of Pediatrics	Indiana School of Medicine	Indianapolis, Indiana, USA	ECHO Cohort Study Site Co-Investigator	UG3/UH3OD023288 (Cynthia McEvoy)	rtepper@iu.edu
Craig J.	Newschaffer		PhD	College of Health and Human Development	Penn State	State College, Pennsylvania, USA	ECHO Cohort Study Site Principal Investigator	UG3/UH3OD023342 (Kristen Lyall)	newschaffer@psu.edu
Kristen	Lyall		ScD	AJ Drexel Autism Institute	Drexel University	Philadelphia, Pennsylvania, USA	ECHO Cohort Study Site Principal Investigator	UG3/UH3OD023342 (Kristen Lyall)	kld98@drexel.edu
Heather E.	Volk		PhD	Mental Health	Johns Hopkins University	Baltimore, Maryland, USA	ECHO Cohort Study Site Principal Investigator	UG3/UH3OD023342 (Kristen Lyall)	hvolk1@jhu.edu
Rebecca	Landa		PhD	Department of Psychiatry and Behavioral Sciences	Center for Autism and Related Disorders, Kennedy Krieger Institute, Johns Hopkins University	Baltimore, Maryland, USA	ECHO Cohort Study Site Co-Investigator	UG3/UH3OD023342 (Kristen Lyall)	landa@kennedykrieger.org
Sally	Ozonoff		PhD	MIND Institute, Department of Psychiatry	University of California Davis	Sacramento, California, USA	ECHO Cohort Study Site Co-Investigator	UG3/UH3OD023342 (Kristen Lyall)	sozonoff@ucdavis.edu
Joseph	Piven		MD	Department of Psychiatry	University of North Carolina	Chapel Hill, North Carolina, USA	ECHO Cohort Study Site Co-Investigator	UG3/UH3OD023342 (Kristen Lyall)	jpiven@med.unc.edu
Heather	Hazlett		PhD	Department of Psychiatry	University of North Carolina	Chapel Hill, North Carolina, USA	ECHO Cohort Study Site Co-Investigator	UG3/UH3OD023342 (Kristen Lyall)	heather_cody@med.unc.edu
Juhi	Pandey		PhD	Center for Autism Research	Children’s Hospital of Philadelphia	Philadelphia, Pennsylvania, USA	ECHO Cohort Study Site Co-Investigator	UG3/UH3OD023342 (Kristen Lyall)	pandeyj@chop.edu
Robert	Schultz		PhD	Center for Autism Research	Children’s Hospital of Philadelphia	Philadelphia, Pennsylvania, USA	ECHO Cohort Study Site Co-Investigator	UG3/UH3OD023342 (Kristen Lyall)	schultzrt@chop.edu
Steven	Dager		PhD	Department of Radiology	University of Washington	Seattle, Washington, USA	ECHO Cohort Study Site Co-Investigator	UG3/UH3OD023342 (Kristen Lyall)	srd@uw.edu
Kelly	Botteron		PhD	Department of Psychiatry	Washington University	St Louis, Missouri, USA	ECHO Cohort Study Site Co-Investigator	UG3/UH3OD023342 (Kristen Lyall)	botteronk@wustl.edu
Daniel	Messinger		PhD	Department of Psychology	University of Miami	Miami, Florida, USA	ECHO Cohort Study Site Co-Investigator	UG3/UH3OD023342 (Kristen Lyall)	dmessinger@miami.edu
Wendy	Stone		PhD	Department of Psychology	University of Washington	Seattle, Washington, USA	ECHO Cohort Study Site Co-Investigator	UG3/UH3OD023342 (Kristen Lyall)	stonew@uw.edu
Jennifer	Ames		PhD	Kaiser Permanente Division of Research	Kaiser Permanente	Oakland, California, USA	ECHO Cohort Study Site Co-Investigator	UG3/UH3OD023342 (Kristen Lyall)	Jennifer.L.Ames@kp.org
Thomas G.	O’Connor		PhD	Departments of Psychiatry, Neuroscience, Obstetrics and Gynecology	University of Rochester	Rochester, New York, USA	ECHO Cohort Study Site Principal Investigator	UG3/UH3OD023349 (Thomas O’Connor)	tom_oconnor@urmc.Rochester.edu
Richard K.	Miller		PhD	Departments of Obstetrics and Gynecology	University of Rochester	Rochester, New York, USA	ECHO Cohort Study Site Principal Investigator	UG3/UH3OD023349 (Thomas O’Connor)	richardk_miller@urmc.rochester.edu
Emily	Oken		MD, MPH	Division of Chronic Disease Research Across the Lifecourse, Department of Population Medicine	Harvard Pilgrim Health Care Institute and Harvard Medical School	Boston, Massachusetts, USA	ECHO Cohort Study Site Principal Investigator	UH3OD023286 and UG3OD035533 (Emily Oken)	emily_oken@hms.harvard.edu
Michele R.	Hacker		ScD	Department of Obstetrics and Gynecology	Beth Israel Deaconess Medical Center	Boston, Massachusetts, USA	ECHO Cohort Study Site Principal Investigator	UG3OD035533 (Emily Oken)	mhacker@bidmc.harvard.edu
Tamarra	James-Todd		PhD	Department of Environmental Health	Harvard Chan School of Public Health	Boston, Massachusetts, USA	ECHO Cohort Study Site Principal Investigator	UG3OD035533 (Emily Oken)	tjtodd@hsph.harvard.edu
T. Michael	O’Shea	Jr	MD, MPH	Division of Neonatology, Department of Pediatrics	University of North Carolina School of Medicine	Chapel Hill, North Carolina, USA	ECHO Cohort Study Site Principal Investigator	UG3/UH3OD023348 (Mike O’Shea), UH3OD023347 (Barry Lester)	moshea52@email.unc.edu
Rebecca C.	Fry		PhD	Department of Environmental Sciences and Engineering	University of North Carolina Gillings School of Global Public Health	Chapel Hill, North Carolina, USA	ECHO Cohort Study Site Principal Investigator	UG3/UH3OD023348 (Mike O’Shea)	rfry@unc.edu
Jean A.	Frazier		MD	EK Shriver Center and Psychiatry	UMASS Chan Medical School	Worcester, Massachusetts, USA	ECHO Cohort Study Site Co-Investigator	UG3/UH3OD023348 (Mike O’Shea)	jean.frazier@umassmed.edu
Rachana	Singh		MD, MS	Department of Pediatrics	Tufts University School of Medicine	Boston, Massachusetts, USA	ECHO Cohort Study Site Co-Investigator	UG3/UH3OD023348 (Mike O’Shea)	jean.frazier@umassmed.edu
Caitlin	Rollins		MD, SM	Department of Neurology	Harvard Medical School	Boston, Massachusetts, USA	ECHO Cohort Study Site Co-Investigator	UG3/UH3OD023348 (Mike O’Shea)	Rachana.Singh1@tuftsmedicine.org
Angela	Montgomery		MD	Division of Neonatology, Department of Pediatrics	Yale School of Medicine	New Haven, Connecticut, USA	ECHO Cohort Study Site Co-Investigator	UG3/UH3OD023348 (Mike O’Shea)	angela.montgomery@yale.edu
Ruben	Vaidya		MD	Department of Pediatrics	University of Massachusetts Chan Medical School-Baystate	Springfield, Massachusetts, USA	ECHO Cohort Study Site Co-Investigator	UG3/UH3OD023348 (Mike O’Shea)	Ruben.VaidyaMD@baystatehealth.org
Robert M.	Joseph		PhD	Department of Anatomy & Neurobiology	Boston University Chobanian & Avedisian School of Medicine	Boston, Massachusetts, USA	ECHO Cohort Study Site Co-Investigator	UG3/UH3OD023348 (Mike O’Shea)	rmjoseph@bu.edu
Lisa K.	Washburn		MD	Pediatrics	Wake Forest School of Medicine	Winston-Salem, North Carolina, USA	ECHO Cohort Study Site Co-Investigator	UG3/UH3OD023348 (Mike O’Shea)	lwcadmus@gmail.com
Semsa	Gogcu		MD, MPH	Section of Neonatology, Department of Pediatrics; Department of Pediatrics	Wake Forest School of Medicine; Wake Forest University School of Medicine/Atrium Health Wake Forest	Winston-Salem, North Carolina, USA	ECHO Cohort Study Site Co-Investigator	UG3/UH3OD023348 (Mike O’Shea), UG3OD035513 (Annemarie Stroustrup), UH3OD023320 (Judy Aschner)	sgogcu@wakehealth.edu
Kelly	Bear		DO	Section of Neonatology, Department of Pediatrics	ECU Health	Greenville, North Carolina, USA	ECHO Cohort Study Site Co-Investigator	UG3/UH3OD023348 (Mike O’Shea)	beark17@ecu.edu
Julie V.	Rollins		MA	Division of Neonatology, Department of Pediatrics	University of North Carolina School of Medicine	Chapel Hill, North Carolina, USA	ECHO Cohort Study Site Award Project Director	UG3/UH3OD023348 (Mike O’Shea)	julie.rollins@unc.edu
Stephen R.	Hooper		PhD	Department of Health Sciences	School of Medicine, University of North Carolina at Chapel Hill	Chapel Hill, North Carolina, USA	ECHO Cohort Study Site Co-Investigator	UG3/UH3OD023348 (Mike O’Shea)	stephen_hooper@med.unc.edu
Genevieve	Taylor		MD	Pediatrics	School of Medicine, University of North Carolina at Chapel Hill	Chapel Hill, North Carolina, USA	ECHO Cohort Study Site Co-Investigator	UG3/UH3OD023348 (Mike O’Shea)	gtaylor@med.unc.edu
Wesley	Jackson		MD, MPH	Division of Neonatology, Department of Pediatrics	University of North Carolina School of Medicine	Chapel Hill, North Carolina, USA	ECHO Cohort Study Site Co-Investigator	UG3/UH3OD023348 (Mike O’Shea)	wesley.jackson@unc.edu
Amanda	Thompson		PhD	Department of Anthropology, Department of Nutrition	University of North Carolina at Chapel Hill; Gillings School of Global Public Health, University of North Carolina at Chapel Hill	Chapel Hill, North Carolina, USA	ECHO Cohort Study Site Co-Investigator	UG3/UH3OD023348 (Mike O’Shea)	althomps@email.unc.edu
Julie	Daniels		PhD	Epidemiology and Maternal and Child Health	University of North Carolina at Chapel Hill; Gillings School of Global Public Health, University of North Carolina at Chapel Hill	Chapel Hill, North Carolina, USA	ECHO Cohort Study Site Co-Investigator	UG3/UH3OD023348 (Mike O’Shea)	julie_daniels@unc.edu
Michelle	Hernandez		MD	Pediatrics	School of Medicine, University of North Carolina at Chapel Hill	Chapel Hill, North Carolina, USA	ECHO Cohort Study Site Co-Investigator	UG3/UH3OD023348 (Mike O’Shea)	michelle_hernandez@med.unc.edu
Kun	Lu		PhD	Environmental Sciences and Engineering	Gillings School of Global Public Health, University of North Carolina at Chapel Hill	Chapel Hill, North Carolina, USA	ECHO Cohort Study Site Co-Investigator	UG3/UH3OD023348 (Mike O’Shea)	kunlu@unc.edu
Michael	Msall		MD	Kennedy Research Center on Intellectual and Neurodevelopmental Disabilities	University of Chicago Medicine: Comer Children’s Hospital	Chicago, Illinois, USA	ECHO Cohort Study Site Co-Investigator	UG3/UH3OD023348 (Mike O’Shea)	mmsall@peds.bsd.uchicago.edu
Madeleine	Lenski		MSPH	Department of Epidemiology and Biostatistics	Michigan State University	East Lansing, Michigan, USA	ECHO Cohort Study Site Co-Investigator	UG3/UH3OD023348 (Mike O’Shea)	lenskim@msu.edu
Rawad	Obeid		MD	Pediatrics	Beaumont Hospital	Royal Oak, Michigan, USA	ECHO Cohort Study Site Co-Investigator	UG3/UH3OD023348 (Mike O’Shea)	Rawad.Obeid@beaumont.org
Steven L.	Pastyrnak		PhD	Pediatrics	Corewell Health, Helen DeVos Children’s Hospital	Grand Rapids, Michigan, USA	ECHO Cohort Study Site Co-Investigator	UG3/UH3OD023348 (Mike O’Shea), UH3OD023347 (Barry Lester)	Steve.Pastyrnak@helendevoschildrens.org
Elizabeth	Jensen		PhD	Epidemiology and Prevention	Wake Forest University School of Medicine	Winston-Salem, North Carolina, USA	ECHO Cohort Study Site Co-Investigator	UG3/UH3OD023348 (Mike O’Shea)	ejensen@wakehealth.edu
Christina	Sakai		MD	Pediatrics	Mass General Hospital for Children	Boston, Massachusetts, USA	ECHO Cohort Study Site Co-Investigator	UG3/UH3OD023348 (Mike O’Shea)	Christina.sakai@gmail.com
Hudson	Santos		RN, PhD	Dean’s Office, Graduate School, School of Nursing and Health Studies	University of Miami	Coral Gables, Florida, USA	ECHO Cohort Study Site Principal Investigator	UG3/UH3OD023348 (Mike O’Shea), UG3OD035542 (Hudson Santos)	hsantos@miami.edu
Jean M.	Kerver		PhD, MSc, RD	Departments of Epidemiology & Biostatistics, and Pediatrics & Human Development	Michigan State University, College of Human Medicine	East Lansing, Michigan, USA	ECHO Cohort Study Site Principal Investigator	UG3/UH3OD023285 (Jean Kerver)	kerverje@msu.edu
Nigel	Paneth		MD, MPH	Departments of Epidemiology & Biostatistics, and Pediatrics & Human Development	Michigan State University, College of Human Medicine	East Lansing, Michigan, USA	ECHO Cohort Study Site Principal Investigator	UG3/UH3OD023285 (Jean Kerver)	paneth@msu.edu
Charles J.	Barone	II	MD, FAAP	Department of Pediatrics	Henry Ford Health	Detroit, Michigan, USA	ECHO Cohort Study Site Principal Investigator	UG3/UH3OD023285 (Jean Kerver), UG3/UH3OD023282 (James Gern)	cbarone1@hfhs.org
Michael R.	Elliott		PhD	Department of Biostatistics	University of Michigan	Ann Arbor, Michigan, USA	ECHO Cohort Study Site Principal Investigator	UG3/UH3OD023285 (Jean Kerver)	mrelliot@umich.edu
Douglas M.	Ruden		PhD	Department of Obstetrics and Gynecology, Institute of Environmental Health Sciences (IEHS), C.S. Mott Center for Human Health and Development	Wayne State University	Detroit, Michigan, USA	ECHO Cohort Study Site Principal Investigator	UG3/UH3OD023285 (Jean Kerver)	douglasr@wayne.edu
Chris	Fussman		MS	Lifecourse Epidemiology and Genomics Division	Michigan Department of Health and Human Services (MDHHS)	Lansing, Michigan, USA	ECHO Cohort Study Site Principal Investigator	UG3/UH3OD023285 (Jean Kerver)	fussmanc@michigan.gov
Julie B.	Herbstman		PhD	Department of Environmental Health Sciences	Columbia University Mailman School of Public Health	New York, New York, USA	ECHO Cohort Study Site Principal Investigator	UG3/UH3OD023290 (Julie Herbstman)	jh2678@cumc.columbia.edu
Amy	Margolis		PhD	Department of Psychiatry	Columbia University Irving Medical Center	New York, New York, USA	ECHO Cohort Study Site Principal Investigator	UG3/UH3OD023290 (Julie Herbstman)	amy.margolis@nyspi.columbia.edu
Susan L.	Schantz		PhD	Beckman Institute for Advanced Science and Technology; Department of Comparative Biosciences	University of Illinois Urbana-Champaign	Urbana, Illinois, USA	ECHO Cohort Study Site Principal Investigator	UG3/UH3OD023272 (Susan Schantz)	schantz@illinois.edu
Sarah Dee	Geiger		PhD	Beckman Institute for Advanced Science and Technology; Department of Kinesiology and Community Health	University of Illinois Urbana-Champaign	Urbana, Illinois, USA	ECHO Cohort Study Site Co-Investigator	UG3/UH3OD023272 (Susan Schantz)	smurphy7@illinois.edu
Andrea	Aguiar		PhD	Beckman Institute for Advanced Science and Technology; Department of Comparative Biosciences	University of Illinois Urbana-Champaign	Urbana, Illinois, USA	ECHO Cohort Study Site Co-Investigator	UG3/UH3OD023272 (Susan Schantz)	aaguiar@illinois.edu
Karen	Tabb		PhD, MSW	Beckman Institute for Advanced Science and Technology; Department of Social Work	University of Illinois Urbana-Champaign	Urbana, Illinois, USA	ECHO Cohort Study Site Co-Investigator	UG3/UH3OD023272 (Susan Schantz)	ktabb@illinois.edu
Rita	Strakovsky		PhD	Department of Food Science and Human Nutrition	Michigan State University	East Lansing, Michigan, USA	ECHO Cohort Study Site Co-Investigator	UG3/UH3OD023272 (Susan Schantz)	strakovs@msu.edu
Tracey	Woodruff		PhD, MPH	Program on Reproductive Health and the Environment	University of California, San Francisco	San Francisco, California, USA	ECHO Cohort Study Site Principal Investigator	UG3/UH3OD023272 (Susan Schantz)	tracey.woodruff@ucsf.edu
Rachel	Morello-Frosch		PhD, MPH	Department of Environmental Science, Policy and Management and School of Public Health	University of California, Berkeley	Berkeley, California, USA	ECHO Cohort Study Site Principal Investigator	UG3/UH3OD023272 (Susan Schantz)	rmf@berkeley.edu
Amy	Padula		PhD	Program on Reproductive Health and the Environment	University of California, San Francisco	San Francisco, California, USA	ECHO Cohort Study Site Co-Investigator	UG3/UH3OD023272 (Susan Schantz)	amy.padula@ucsf.edu
Joseph B.	Stanford		MD, MSPH	Department of Family and Preventive Medicine	Spencer Fox Eccles School of Medicine, University of Utah	Salt Lake City, Utah, USA	ECHO Cohort Study Site Principal Investigator	UG3/UH3OD023249 (Joseph Stanford)	joseph.stanford@utah.edu
Christina A.	Porucznik		PhD, MSPH	Department of Family and Preventive Medicine	Spencer Fox Eccles School of Medicine, University of Utah	Salt Lake City, Utah, USA	ECHO Cohort Study Site Principal Investigator	UG3/UH3OD023249 (Joseph Stanford)	christy.porucznik@utah.edu
Angelo P.	Giardino		MD, PhD	Department of Pediatrics	Spencer Fox Eccles School of Medicine, University of Utah	Salt Lake City, Utah, USA	ECHO Cohort Study Site Principal Investigator	UG3/UH3OD023249 (Joseph Stanford)	giardino@hsc.utah.edu
Rosalind J.	Wright		MD, MPH	Department of Environmental Medicine & Public Health	Icahn School of Medicine at Mount Sinai	New York, New York, USA	ECHO Cohort Study Site Principal Investigator	UG3/UH3OD023337 (Rosalind Wright)	rosalind.wright@mssm.edu
Robert O.	Wright		MD, MPH	Department of Environmental Medicine & Public Health	Icahn School of Medicine at Mount Sinai	New York, New York, USA	ECHO Cohort Study Site Principal Investigator	UG3/UH3OD023337 (Rosalind Wright)	robert.wright@mssm.edu
Brent	Collett		PhD	Department of Psychiatry and Behavioral Medicine	University of Washington, Seattle Children’s Research Institute	Seattle, Washington, USA	ECHO Cohort Study Site Principal Investigator	UG3OD035508 (Sheela Sathyanarayana)	brent.collett@seattlechildrens.org
Nicole	Baumann-Blackmore		MD	Department of Pediatrics	University of Wisconsin School of Medicine and Public Health	Madison, Wisconsin, USA	ECHO Cohort Study Site Co-Investigator	UG3OD035509 (Anne Marie Singh)	nlbaumann@wisc.edu
Ronald	Gangnon		PhD	Department of Population Health Sciences	University of Wisconsin	Madison, Wisconsin, USA	ECHO Cohort Study Site Co-Investigator	UG3OD035509 (Anne Marie Singh)	ronald@biostat.wisc.edu
Daniel J.	Jackson		MD	Department of Pediatrics	University of Wisconsin School of Medicine and Public Health	Madison, Wisconsin, USA	ECHO Cohort Study Site Co-Investigator	UG3OD035509 (Anne Marie Singh)	djj@medicine.wisc.edu
Chris G.	McKennan		PhD	Department of Statistics	University of Pittsburgh	Pittsburgh, Pennsylvania, USA	ECHO Cohort Study Site Co-Investigator	UG3OD035509 (Anne Marie Singh)	CHM195@pitt.edu
Jo	Wilson		MD	Department of Pediatrics	University of Wisconsin School of Medicine and Public Health	Madison, Wisconsin, USA	ECHO Cohort Study Site Co-Investigator	UG3OD035509 (Anne Marie Singh)	wilson54@wisc.edu
Matt	Altman		MD	Department of Medicine	University of Washington	Seattle, Washington, USA	ECHO Cohort Study Site Co-Investigator	UG3OD035509 (Anne Marie Singh)	maltman@benaroyaresearch.org
Judy L.	Aschner		MD	Department of Pediatrics	Albert Einstein College of Medicine; Hackensack Meridian School of Medicine; Center for Discovery and Innovation	Bronx, New York, USA; Nutley, New Jersey, USA	ECHO Cohort Study Site Principal Investigator	UH3OD023320 and UG3OD035546 (Judy Aschner), UG3OD035513 (Annemarie Stroustrup)	judy.aschner@einsteinmed.edu; judy.aschner@hmhn.org
Annemarie	Stroustrup		MD, MPH	Department of Pediatrics	Northwell Health, Cohen Children’s Medical Center, and the Zucker School of Medicine at Hofstra/Northwell	New Hyde Park, New York, USA	ECHO Cohort Study Site Principal Investigator	UH3OD023320 (Judy Aschner), UG3OD035513 (Annemarie Stroustrup)	astroustrup@northwell.edu
Stephanie L.	Merhar		MD, MS	Department of Pediatrics	Cincinnati Children’s	Cincinnati, Ohio, USA	ECHO Cohort Study Site Co-Investigator	UH3OD023320 (Judy Aschner), UG3OD035513 (Annemarie Stroustrup)	stephanie.merhar@cchmc.org
Paul E.	Moore		MD	Department of Pediatrics	Vanderbilt University Medical Center	Nashville, Tennessee, USA	ECHO Cohort Study Site Co-Investigator	UH3OD023320 (Judy Aschner), UG3OD035513 (Annemarie Stroustrup)	paul.moore@vumc.org
Gloria S.	Pryhuber		MD	Department of Pediatrics	University of Rochester Medical Center	Rochester, New York, USA	ECHO Cohort Study Site Co-Investigator	UH3OD023320 (Judy Aschner)	gloria_pryhuber@urmc.rochester.edu
Mark	Hudak		MD	Department of Pediatrics	University of Florida College of Medicine	Jacksonville, Florida, USA	ECHO Cohort Study Site Co-Investigator	UH3OD023320 (Judy Aschner)	mark.hudak@jax.ufl.edu
Ann Marie	Reynolds Lyndaker		MD, MPH	Department of Pediatrics	University of Buffalo Jacobs School of Medicine and Biomedical Sciences	Buffalo, New York, USA	ECHO Cohort Study Site Co-Investigator	UH3OD023320 (Judy Aschner)	amr1@buffalo.edu
Andrea L.	Lampland		MD	Department of Pediatrics	Children’s Minnesota	Minneapolis, Minnesota, USA	ECHO Cohort Study Site Co-Investigator	UH3OD023320 (Judy Aschner)	andrea.lampland@childrensms.org
Burton	Rochelson		MD	Department of Obstetrics and Gynecology	Northwell Health and the Zucker School of Medicine at Hofstra/Northwell	New Hyde Park, New York, USA	ECHO Cohort Study Site Principal Investigator	UG3OD035532 (Annemarie Stroustrup)	brochels@northwell.edu
Sophia	Jan		MD, MSHP	Department of Pediatrics	Northwell Health, Cohen Children’s Medical Center, and the Zucker School of Medicine at Hofstra/Northwell	New Hyde Park, New York, USA	ECHO Cohort Study Site Co-Investigator	UG3OD035532 (Annemarie Stroustrup)	sjan1@northwell.edu
Matthew J.	Blitz		MD, MBA	Department of Obstetrics and Gynecology	Northwell Health and the Zucker School of Medicine at Hofstra/Northwell	New Hyde Park, New York, USA	ECHO Cohort Study Site Co-Investigator	UG3OD035532 (Annemarie Stroustrup)	mblitz@northwell.edu
Michelle W.	Katzow		MD, MS	Department of Pediatrics	Northwell Health, Cohen Children’s Medical Center, and the Zucker School of Medicine at Hofstra/Northwell	New Hyde Park, New York, USA	ECHO Cohort Study Site Co-Investigator	UG3OD035532 (Annemarie Stroustrup)	mkatzow@northwell.edu
Zenobia	Brown		MD, MPH	Department of Science Education	Northwell Health and the Zucker School of Medicine at Hofstra/Northwell	New Hyde Park, New York, USA	ECHO Cohort Study Site Co-Investigator	UG3OD035532 (Annemarie Stroustrup)	zbrown2@northwell.edu
Codruta	Chiuzan		PhD	Institute of Health System Science	Northwell Health, Feinstein Institutes for Medical Research	Manhasset, New York, USA	ECHO Cohort Study Site Co-Investigator	UG3OD035532 (Annemarie Stroustrup)	cchiuzan@northwell.edu
Timothy	Rafael		MD	Department of Obstetrics and Gynecology	Northwell Health and the Zucker School of Medicine at Hofstra/Northwell	New Hyde Park, New York, USA	ECHO Cohort Study Site Co-Investigator	UG3OD035532 (Annemarie Stroustrup)	trafael@northwell.edu
Dawnette	Lewis		MD, MPH	Department of Obstetrics and Gynecology	Northwell Health and the Zucker School of Medicine at Hofstra/Northwell	New Hyde Park, New York, USA	ECHO Cohort Study Site Co-Investigator	UG3OD035532 (Annemarie Stroustrup)	dlewis@northwell.edu
Natalie	Meirowitz		MD	Department of Obstetrics and Gynecology	Northwell Health and the Zucker School of Medicine at Hofstra/Northwell	New Hyde Park, New York, USA	ECHO Cohort Study Site Co-Investigator	UG3OD035532 (Annemarie Stroustrup)	nmeirowi@northwell.edu
Brenda	Poindexter		MD	Department of Pediatrics	Children’s Healthcare of Atlanta Emory University	Atlanta, Georgia, USA	ECHO Cohort Study Site Co-Investigator	UH3OD023320 (Judy Aschner)	breda.pointdexter@emory.edu
Tebeb	Gebretsadik		MPH	Department of Biostatistics	Vanderbilt University Medical Center	Nashville, Tennessee, USA	ECHO Cohort Study Site Principal Investigator	UG3OD035516 and UG3OD035517 (Tina Hartert)	tebeb.gebretsadik@vumc.org
Sarah	Osmundson		MD, MSC	Department of Obstetrics and Gynecology	Vanderbilt University Medical Center	Nashville, Tennessee, USA	ECHO Cohort Study Site Principal Investigator	UG3OD035517 (Tina Hartert)	sarah.osmundson@vumc.org
Jennifer K.	Straughen		PhD	Department of Public Health Sciences	Henry Ford Health	Detroit, Michigan, USA	ECHO Cohort Study Site Principal Investigator	UG3OD035518 (Jennifer Straughen)	jstraug1@hfhs.org
Amy	Eapen		MD	Division of Allergy and Clinical Immunology	Henry Ford Health	Detroit, Michigan, USA	ECHO Cohort Study Site Principal Investigator	UG3OD035518 (Jennifer Straughen)	aeapen1@hfhs.org
Andrea	Cassidy-Bushrow		PhD	Department of Public Health Sciences	Henry Ford Health	Detroit, Michigan, USA	ECHO Cohort Study Site Co-Investigator	UG3/UH3OD023282 (James Gern)	acassid1@hfhs.org
Ganesa	Wegienka		PhD	Department of Public Health Sciences	Henry Ford Health	Detroit, Michigan, USA	ECHO Cohort Study Site Co-Investigator	UG3/UH3OD023282 (James Gern)	gwegien1@hfhs.org
Alex	Sitarik		MPH	Department of Public Health Sciences	Henry Ford Health	Detroit, Michigan, USA	ECHO Cohort Study Site Biostatistician	UG3/UH3OD023282 (James Gern)	asitari1@hfhs.org
Kim	Woodcroft		PhD	Department of Public Health Sciences	Henry Ford Health	Detroit, Michigan, USA	ECHO Cohort Study Site Co-Investigator	UG3OD035518 (Jennifer Straughen), UG3/UH3OD023282 (James Gern)	kwoodcr1@hfhs.org
Audrey	Urquhart		MPH	Department of Public Health Sciences	Henry Ford Health	Detroit, Michigan, USA	ECHO Cohort Study Site Epidemiologist	UG3OD035518 (Jennifer Straughen), UG3/UH3OD023282 (James Gern)	aurquha1@hfhs.org
Albert	Levin		PhD	Department of Public Health Sciences	Henry Ford Health	Detroit, Michigan, USA	ECHO Cohort Study Site Co-Investigator	UG3OD035518 (Jennifer Straughen)	alevin1@hfhs.org
Tisa	Johnson-Hooper		MD	Department of Pediatrics	Henry Ford Health	Detroit, Michigan, USA	ECHO Cohort Study Site Co-Investigator	UG3OD035518 (Jennifer Straughen)	tjohnso2@hfhs.org
Brent	Davidson		MD	Department of Women’s Health	Henry Ford Health	Detroit, Michigan, USA	ECHO Cohort Study Site Co-Investigator	UG3/UH3OD023282 (James Gern)	bdavids1@hfhs.org
Tengfei	Ma		PhD	Department of Public Health Sciences	Henry Ford Health	Detroit, Michigan, USA	ECHO Cohort Study Site Co-Investigator	UG3OD035518 (Jennifer Straughen)	tengfei.ma@hfhs.org
Emily S.	Barrett		PhD	Department of Biostatistics and Epidemiology	Environmental and Occupational Health Sciences Institute, Rutgers University	Piscataway, New Jersey, USA	ECHO Cohort Study Site Principal Investigator	UG3OD035527 (Emily S Barrett)	emily.barrett@eoshi.rutgers.edu
Martin J.	Blaser		MD	Center for Advanced Biotechnology & Medicine	Rutgers University	Piscataway, New Jersey, USA	ECHO Cohort Study Site Principal Investigator	UG3OD035527 (Emily S Barrett)	blaser@cabm.rutgers.edu
Maria Gloria	Dominguez-Bello		PhD	Departments of Biochemistry and Microbiology & Anthropology	Rutgers University	New Brunswick, New Jersey, USA	ECHO Cohort Study Site Principal Investigator	UG3OD035527 (Emily S Barrett)	mg.dominguez-bello@rutgers.edu
Daniel B.	Horton		MD	Department of Pediatrics	Robert Wood Johnson Medical School, Rutgers University	New Brunswick, New Jersey, USA	ECHO Cohort Study Site Principal Investigator	UG3OD035527 (Emily S Barrett)	daniel.horton@rutgers.edu
Manuel	Jimenez		MD	Departments of Pediatrics, Family Medicine, and Community Health	Robert Wood Johnson Medical School, Rutgers University	New Brunswick, New Jersey, USA	ECHO Cohort Study Site Principal Investigator	UG3OD035527 (Emily S Barrett)	jimenema@rwjms.rutgers.edu
Todd	Rosen		MD	Department of Obstetrics, Gynecology, and Reproductive Sciences	Robert Wood Johnson Medical School, Rutgers University	New Brunswick, New Jersey, USA	ECHO Cohort Study Site Co-Investigator	UG3OD035527 (Emily S Barrett)	rosentj@rwjms.rutgers.edu
Kristy	Palomares		MD, PhD	Department of Obstetrics and Gynecology	Saint Peter’s University Hospital	New Brunswick, New Jersey, USA	ECHO Cohort Study Site Co-Investigator	UG3OD035527 (Emily S Barrett)	kpalomares@saintpetersuh.com
Lyndsay A.	Avalos		PhD, MPH	Division of Research	Kaiser Permanente Northern California	Oakland, California, USA	ECHO Cohort Study Site Principal Investigator	UG3OD035540 (Monique Marie Hedderson)	Lyndsay.A.Avalos@kp.org
Yeyi	Zhu		PhD, MS	Division of Research	Kaiser Permanente Northern California	Oakland, California, USA	ECHO Cohort Study Site Principal Investigator	UG3OD035540 (Monique Marie Hedderson)	Yeyi.Zhu@kp.org
Kelly J .	Hunt		PhD	Department of Public Health Sciences	Medical University of South Carolina	Charleston, South Carolina, USA	ECHO Cohort Study Site Principal Investigator	UG3OD035543 (Kelly J Hunt)	huntke@musc.edu
Roger B.	Newman		MD	Department of Obstetrics and Gynecology	Medical University of South Carolina	Charleston, South Carolina, USA	ECHO Cohort Study Site Principal Investigator	UG3OD035543 (Kelly J Hunt)	newmanr@musc.edu
Michael S.	Bloom		PhD	Department of Global and Community Health	George Mason University	Fairfax, Virginia, USA	ECHO Cohort Study Site Principal Investigator	UG3OD035543 (Kelly J Hunt)	mbloom22@gmu.edu
Mallory H.	Alkis		MD	Department of Obstetrics and Gynecology	Medical University of South Carolina	Charleston, South Carolina, USA	ECHO Cohort Study Site Co-Investigator	UG3OD035543 (Kelly J Hunt)	hudsonm@musc.edu
James R.	Roberts		MD, MPH	Department of Pediatrics	Medical University of South Carolina	Charleston, South Carolina, USA	ECHO Cohort Study Site Co-Investigator	UG3OD035543 (Kelly J Hunt)	robertsj@musc.edu
Sunni L.	Mumford		PhD	Department of Biostatistics, Epidemiology and Informatics; Department of Obstetrics and Gynecology	University of Pennsylvania Perelman School of Medicine	Philadelphia, Pennsylvania, USA	ECHO Cohort Study Site Principal Investigator	UG3OD035537 (Sunni L Mumford)	sunni.mumford@pennmedicine.upenn.edu
Heather H.	Burris		MD, MPH	Division of Neonatology, Department of Pediatrics	Children’s Hospital of Philadelphia; University of Pennsylvania Perelman School of Medicine	Philadelphia, Pennsylvania, USA	ECHO Cohort Study Site Principal Investigator	UG3OD035537 (Sunni L Mumford)	BURRISH@chop.edu
Sara B.	DeMauro		MD, MSCE	Division of Neonatology, Department of Pediatrics	Children’s Hospital of Philadelphia; University of Pennsylvania Perelman School of Medicine	Philadelphia, Pennsylvania, USA	ECHO Cohort Study Site Principal Investigator	UG3OD035537 (Sunni L Mumford)	DEMAURO@chop.edu
Lynn M.	Yee		MD, MPH	Division of Maternal-Fetal Medicine, Department of Obstetrics & Gynecology	Feinberg School of Medicine, Northwestern University	Chicago, Illinois, USA	ECHO Cohort Study Site Principal Investigator	UG3OD035546 (Judy Aschner)	lynn.yee@northwestern.edu
Aaron	Hamvas		MD	Division of Neonatology, Department of Pediatrics	Ann & Robert H. Lurie Children’s Hospital, Feinberg School of Medicine, Northwestern University	Chicago, Illinois, USA	ECHO Cohort Study Site Principal Investigator	UG3OD035546 (Judy Aschner)	ahamvas@luriechildrens.org
Antonia F.	Olidipo		MD, MSCI	Division of Maternal-Fetal Medicine, Department of Obstetrics & Gynecology	Hackensack University Medical Center, Hackensack Meridian School of Medicine	Nutley, New Jersey, USA	ECHO Cohort Study Site Co-Investigator	UG3OD035546 (Judy Aschner)	antonia.olidipo@hmhn.org
Andrew S.	Haddad		MD	Division of Maternal-Fetal Medicine, Department of Obstetrics & Gynecology	Hackensack University Medical Center, Hackensack Meridian School of Medicine	Nutley, New Jersey, USA	ECHO Cohort Study Site Co-Investigator	UG3OD035546 (Judy Aschner)	andrews.haddad@hmhn.org
Lisa R.	Eiland		MD	Division of Neonatology, Department of Pediatrics	Hackensack University Medical Center, Hackensack Meridian School of Medicine	Nutley, New Jersey, USA	ECHO Cohort Study Site Co-Investigator	UG3OD035546 (Judy Aschner)	lisa.eiland@hmhn.org
Nicole T.	Spillane		MD	Division of Neonatology, Department of Pediatrics	Hackensack University Medical Center, Hackensack Meridian School of Medicine	Nutley, New Jersey, USA	ECHO Cohort Study Site Co-Investigator	UG3OD035546 (Judy Aschner)	nicole.spillane@hmhn.org
Kirin N.	Suri		MD	Division of Developmental and Behavioral Pediatrics, Department of Pediatrics	Hackensack University Medical Center, Hackensack Meridian School of Medicine	Nutley, New Jersey, USA	ECHO Cohort Study Site Co-Investigator	UG3OD035546 (Judy Aschner)	kirin.suri@hmhn.org
Stephanie A.	Fisher		MD, MPH	Division of Maternal-Fetal Medicine, Department of Obstetrics & Gynecology	Feinberg School of Medicine, Northwestern University	Chicago, Illinois, USA	ECHO Cohort Study Site Co-Investigator	UG3OD035546 (Judy Aschner)	stephanie.fisher@northwestern.edu
Jeffrey A.	Goldstein		MD, PhD	Department of Pathology	Feinberg School of Medicine, Northwestern University	Chicago, Illinois, USA	ECHO Cohort Study Site Co-Investigator	UG3OD035546 (Judy Aschner)	ja.goldstein@northwestern.edu
Leena B.	Mithal		MD	Division of Infectious Diseases, Department of Pediatrics	Ann & Robert H. Lurie Children’s Hospital, Feinberg School of Medicine, Northwestern University	Chicago, Illinois, USA	ECHO Cohort Study Site Co-Investigator	UG3OD035546 (Judy Aschner)	lmithal@luriechildrens.org
Raye-Ann O.	DeRegnier		MD	Division of Neonatology, Department of Pediatrics	Ann & Robert H. Lurie Children’s Hospital, Feinberg School of Medicine, Northwestern University	Chicago, Illinois, USA	ECHO Cohort Study Site Co-Investigator	UG3OD035546 (Judy Aschner)	r-deregnier@northwestern.edu
Nathalie L.	Maitre		MD, PhD	Division of Neonatology, Department of Pediatrics	Emory University School of Medicine and Cerebral Palsy Foundation	Atlanta, Georgia, USA; New York, New York, USA	ECHO Cohort Study Site Co-Investigator	UG3OD035546 (Judy Aschner)	nathalie.linda.maitre@emory.edu
Ruby H.N.	Nguyen		PhD, MHS	Division of Epidemiology & Community Health	School of Public Health, University of Minnesota	Minneapolis, Minnesota, USA	ECHO award Principal Investigator	UG3OD035529 (Hong-Ngoc Nguyen)	Nguyen@umn.edu
Meghan M.	JaKa		PhD, MS	Division of Research & Evaluation	HealthPartners Institute	Minneapolis, Minnesota, USA	ECHO site Principal Investigator	UG3OD035529 (Hong-Ngoc Nguyen)	meghan.m.jaka@healthpartners.com
Abbey C.	Sidebottom		PhD, MPH	Care Delivery Research	Allina Health	Minneapolis, Minnesota, USA	ECHO site Principal Investigator	UG3OD035529 (Hong-Ngoc Nguyen)	abbey.sidebottom@allina.com
Michael J.	Paidas		MD	Department of Obstetrics and Gynecology	University of Miami Miller School of Medicine	Miami, Florida, USA	ECHO site Principal Investigator	UG3OD035542 (Hudson Santos)	mxp1440@med.miami.edu
JoNell E.	Potter		APRN, PhD	Department of Obstetrics, Gynecology and Reproductive Sciences	University of Miami Miller School of Medicine	Miami, Florida, USA	ECHO Cohort Study Site Co-Investigator	UG3OD035542 (Hudson Santos)	jpotter2@med.miami.edu
Natale	Ruby		PhD, PsyD	Mailman Center for Child Development	University of Miami Miller School of Medicine	Miami, Florida, USA	ECHO Cohort Study Site Co-Investigator	UG3OD035542 (Hudson Santos)	rnatale@med.miami.edu
Lunthita	Duthely		EdD	Department of Obstetrics, Gynecology and Reproductive Sciences and Department of Public Health Sciences	University of Miami School of Medicine	Miami, Florida, USA	ECHO Cohort Study Site Co-Investigator	UG3OD035542 (Hudson Santos)	LDuthely@med.miami.edu
Arumugam	Jayakumar		PhD	Department of Obstetrics, Gynecology and Reproductive Sciences	University of Miami Miller School of Medicine	Miami, Florida, USA	ECHO Cohort Study Site Co-Investigator	UG3OD035542 (Hudson Santos)	ajayakumar@med.miami.edu
Karen	Young		MD	Department of Pediatrics	University of Miami Miller School of Medicine	Miami, Florida, USA	ECHO Cohort Study Site Co-Investigator	UG3OD035542 (Hudson Santos)	kyoung3@miami.edu
Isabel	Maldonado		MPH, BS	School of Nursing and Health Studies	University of Miami	Miami, Florida, USA	ECHO Cohort Study Site Program Director	UG3OD035542 (Hudson Santos)	icm16@miami.edu
Meghan	Miller		PhD	Psychiatry and Behavioral Sciences; MIND Institute	University of California Davis	Sacramento, California, USA	ECHO Cohort Study Site Co-Investigator	UG3OD035550 (Rebecca Schmidt)	mrhmiller@ucdavis.edu
Jonathan L.	Slaughter		MD, MPH	Center for Perinatal Research, Abigail Wexner Research Institute and Division of Neonatology, Nationwide Children’s Hospital and Department of Pediatrics, College of Medicine and Division of Epidemiology, College of Public Health, The Ohio State University	Nationwide Children’s Hospital and The Ohio State University	Columbus, Ohio, USA	ECHO Cohort Study Site Principal Investigator	UG3OD035536 (Jonathan Slaughter)	jonathan.slaughter@nationwidechildrens.org
Sarah A.	Keim		PhD, MS, MA	Center for Biobehavioral Health, Abigail Wexner Research Institute, Nationwide Children’s Hospital and Department of Pediatrics, College of Medicine and Division of Epidemiology, College of Public Health, The Ohio State University	Nationwide Children’s Hospital and The Ohio State University	Columbus, Ohio, USA	ECHO Cohort Study Site Principal Investigator	UG3OD035536 (Jonathan Slaughter)	Sarah.Keim@nationwidechildrens.org
Courtney D.	Lynch		PhD, MPH	Division of Maternal-Fetal Medicine, Department of Obstetrics and Gynecology, College of Medicine, and Division of Epidemiology, College of Public Health, The Ohio State University	The Ohio State University	Columbus, Ohio, USA	ECHO Cohort Study Site Principal Investigator	UG3OD035536 (Jonathan Slaughter)	Courtney.Lynch@osumc.edu
Kartik K.	Venkatesh		MD, PhD	Division of Maternal-Fetal Medicine, Department of Obstetrics and Gynecology, College of Medicine, and Division of Epidemiology, College of Public Health, The Ohio State University	The Ohio State University	Columbus, Ohio, USA	ECHO Cohort Study Site Principal Investigator	UG3OD035536 (Jonathan Slaughter)	kartik.venkatesh@osumc.edu
Kristina W.	Whitworth		PhD	Center for Precision Environmental Health and Department of Medicine	Baylor College of Medicine	Houston, Texas, USA	ECHO Cohort Study Site Principal Investigator	UG3OD035544 (Kristina Whitworth)	kristina.whitworth@bcm.edu
Elaine	Symanski		PhD	Center for Precision Environmental Health and Department of Medicine	Baylor College of Medicine	Houston, Texas, USA	ECHO Cohort Study Site Principal Investigator	UG3OD035544 (Kristina Whitworth)	elaine.symanski@bcm.edu
Thomas F.	Northrup		PhD	Department of Family and Community Medicine	University of Texas Health Science Center at Houston (UTHealth Houston) McGovern Medical School	Houston, Texas, USA	ECHO Cohort Study Site Principal Investigator	UG3OD035544 (Kristina Whitworth)	thomas.f.northrup@uth.tmc.edu
Hector	Mendez-Figueroa		MD	Department of Obstetrics, Gynecology and Reproductive Sciences	University of Texas Health Science Center at Houston (UTHealth Houston) McGovern Medical School	Houston, Texas, USA	ECHO Cohort Study Site Co-Investigator	UG3OD035544 (Kristina Whitworth)	hector.mendezfigueroa@uth.tmc.edu
Ricardo A.	Mosquera		MD	Department of Pediatrics	University of Texas Health Science Center at Houston (UTHealth Houston) McGovern Medical School	Houston, Texas, USA	ECHO Cohort Study Site Co-Investigator	UG3OD035544 (Kristina Whitworth)	ricardo.a.mosquera@uth.tmc.edu
Margaret R.	Karagas		PhD	Department of Epidemiology	Geisel School of Medicine at Dartmouth	Hanover, New Hampshire, USA	ECHO Cohort Study Site Principal Investigator	UG3/UH3OD023275 (Margaret Karagas)	margaret.karagas@dartmouth.edu
Juliette C.	Madan		MD, MS	Departments of Psychiatry, Pediatrics & Epidemiology	Geisel School of Medicine at Dartmouth, Dartmouth Hitchcock Medical Center	Hanover, New Hampshire, USA	ECHO Cohort Study Site Principal Investigator	UG3/UH3OD023275 (Margaret Karagas)	juliette.madan@dartmouth.edu
Debra M.	MacKenzie		PhD	Community Environmental Health Program, Department of Pharmaceutical Sciences	College of Pharmacy, University of New Mexico Health Sciences Center	Albuquerque, New Mexico, USA	ECHO Cohort Study Site Principal Investigator	UG3/UH3OD023344 (Debra MacKenzie)	dmackenzie@salud.unm.edu
Johnnye L.	Lewis		PhD	Community Environmental Health Program, Department of Pharmaceutical Sciences	College of Pharmacy, University of New Mexico Health Sciences Center	Albuquerque, New Mexico, USA	ECHO Cohort Study Site Principal Investigator	UG3/UH3OD023344 (Debra MacKenzie)	jlewis@cybermesa.com; jlewis@salud.unm.edu
Brandon J.	Rennie		PhD	Center for Development and Disability	University of New Mexico	Albuquerque, New Mexico, USA	ECHO Cohort Study Site Co-Investigator	UG3/UH3OD023344 (Debra MacKenzie)	Brennie@salud.unm.edu
Bennett L.	Leventhal		MD	Community Environmental Health Program, Department of Pharmaceutical Sciences, UNM	College of Pharmacy, University of New Mexico Health Sciences Center; University of Chicago	Albuquerque, New Mexico, USA; Chicago, Illinois, USA	ECHO Cohort Study Site Co-Investigator	UG3/UH3OD023344 (Debra MacKenzie)	Bennett.leventhal@outlook.com
Young Shin	Kim		MD, MS, MPH, PhD	Department of Psychiatry and Behavioral Sciences	University of California, San Francisco	San Francisco, California, USA	ECHO Cohort Study Site Co-Investigator	UG3/UH3OD023344 (Debra MacKenzie)	Youngshin.Kim@ucsf.edu
Somer	Bishop		PhD	Department of Psychiatry and Behavioral Sciences	University of California, San Francisco	San Francisco, California, USA	ECHO Cohort Study Site Co-Investigator	UG3/UH3OD023344 (Debra MacKenzie)	Somer.Bishop@ucsf.com
Sara S.	Nozadi		PhD	Community Environmental Health Program, Department of Pharmaceutical Sciences	College of Pharmacy, University of New Mexico Health Sciences Center	Albuquerque, New Mexico, USA	ECHO Cohort Study Site Co-Investigator	UG3/UH3OD023344 (Debra MacKenzie)	snozadi@unm.edu
Li	Luo		PhD	Department of Internal Medicine	Comprehensive Cancer Center, University of New Mexico Health Sciences Center	Albuquerque, New Mexico, USA	ECHO Cohort Study Site Co-Investigator	UG3/UH3OD023344 (Debra MacKenzie)	lluo@salud.unm.edu
Barry M.	Lester		PhD	Department of Pediatrics, Department of Psychiatry and Human Behavior	Warren Alpert Medical School of Brown University	Providence, Rhode Island, USA	ECHO Cohort Study Site Principal Investigator	UH3OD023347 (Barry Lester)	barry_lester@brown.edu
Carmen J.	Marsit		PhD	Department of Environmental Health	Rollins School of Public Health, Emory University	Atlanta, Georgia, USA	ECHO Cohort Study Site Principal Investigator	UH3OD023347 (Barry Lester)	carmen.j.marsit@emory.edu
Todd	Everson		PhD	Department of Environmental Health	Rollins School of Public Health, Emory University	Atlanta, Georgia, USA	ECHO Cohort Study Site Principal Investigator	UH3OD023347 (Barry Lester)	todd.m.everson@emory.edu
Cynthia M.	Loncar		PhD	Department of Psychiatry and Human Behavior	Warren Alpert Medical School of Brown University	Providence, Rhode Island, USA	ECHO Cohort Study Site Principal Investigator	UH3OD023347 (Barry Lester)	cloncar@kentri.org
Elisabeth C.	McGowan		MD	Department of Pediatrics	Warren Alpert Medical School of Brown University	Providence, Rhode Island, USA	ECHO Cohort Study Site Principal Investigator	UH3OD023347 (Barry Lester)	emcgowan@wihri.org
Stephen J.	Sheinkopf		PhD	Department of Pediatrics	Thompson Center for Autism & Neurodevelopment, University of Missouri	Columbia, Missouri, USA	ECHO Cohort Study Site Principal Investigator	UH3OD023347 (Barry Lester)	ssheinkopf@health.missouri.edu
Brian S.	Carter		MD	Department of Pediatrics	Children’s Mercy-Kansas City	Kansas City, Missouri, USA	ECHO Cohort Study Site Principal Investigator	UH3OD023347 (Barry Lester)	bscarter@cmh.edu
Jennifer	Check		MD	Department of Pediatrics	Wake Forest School of Medicine	Winston, Salem, North Carolina, USA	ECHO Cohort Study Site Principal Investigator	UH3OD023347 (Barry Lester)	jcheck@wakehealth.edu
Jennifer B.	Helderman		MD	Department of Pediatrics	Wake Forest School of Medicine	Winston, Salem, North Carolina, USA	ECHO Cohort Study Site Principal Investigator	UH3OD023347 (Barry Lester)	jhelderm@wakehealth.edu
Charles R.	Neal		MD	Department of Pediatrics	University of Hawaii John A Burns School of Medicine	Honolulu, Hawaii, USA	ECHO Cohort Study Site Principal Investigator	UH3OD023347 (Barry Lester)	cneal@hphmg.org
Lynne M.	Smith		MD	Department of Pediatrics	UCLA Clinical and Translational Science Institute at The Lundquist Institute, Harbor-UCLA Medical Center	Los Angeles, California, USA	ECHO Cohort Study Site Principal Investigator	UH3OD023347 (Barry Lester)	smith@lundquist.org
