# Supplementary material for: Maternal adverse childhood experiences and prenatal stress: Intergenerational transmission and offspring mental health in the ECHO Cohort
Source: Psychol Med. 2026 Mar 11;56:e60. doi: 10.1017/S0033291725103127 (PMC13040406; doi:10.1017/S0033291725103127)

**Supplemental Material**

| Supplemental Methods | 3 |
| --- | --- |
| Tables S1A–C. Correlation Tables | 5 |
| Table S2. Regression Models of Maternal Adverse Childhood Experiences (ACEs) and Child Total Problems Score (Including Age and Sex Moderation and On-The-Path Adjustment), N=2,906 | 8 |
| Table S3. Regression Models of Maternal Prenatal Stress and Child Total Problems Score (Including Age and Sex Moderation and On-The-Path Adjustment), N=4,437 | 10 |
| Table S4. Regression Models of Maternal Adverse Childhood Experiences (ACEs), Maternal Prenatal Stress, and Child Total Problems Score (Including Age and Sex Moderation and On-The-Path Adjustment), N=834 | 12 |
| Table S5. Regression Models of Maternal Adverse Childhood Experiences (ACEs) and Child Externalizing Problems Score, N=2,729 | 14 |
| Table S6. Regression Models of Maternal Prenatal Stress and Child Externalizing Problems Score, N=4,440 | 16 |
| Table S7. Regression Models of Maternal Adverse Childhood Experiences (ACEs), Maternal Prenatal Stress, and Child Externalizing Problems Score, N=834 | 18 |
| Table S8. Regression Models of Maternal Adverse Childhood Experiences (ACEs) and Child Internalizing Problems Score, N=2,665 | 20 |
| Table S9. Regression Models of Maternal Prenatal Stress and Child Internalizing Problems Score, N=4,438 | 22 |
| Table S10. Regression Models of Maternal Adverse Childhood Experiences (ACEs), Maternal Prenatal Stress, and Child Internalizing Problems Score, N=834 | 24 |
| Table S11. Separate Fully Adjusted Logistic Regression Models of Maternal Adverse Childhood Experiences (ACEs) and/or Maternal Prenatal Stress and Binary Child Total Problems Score (T-Score ≥60) | 26 |
| Table S12. Separate Fully Adjusted Logistic Regression Models of Maternal Adverse Childhood Experiences (ACEs) and/or Maternal Prenatal Stress and Binary Child Externalizing Problems Score (T-Score ≥60) | 27 |
| Table S13. Separate Fully Adjusted Logistic Regression Models of Maternal Adverse Childhood Experiences (ACEs) and/or Maternal Prenatal Stress and Binary Child Internalizing Problems Score (T-Score ≥60) | 28 |
| Figure S1. Flowchart of Sample Selection | 29 |
| Figure S2. Map of Participating Sites in the Environmental influences on Child Health Outcomes (ECHO) Cohort | 30 |
| Figure S3. Histogram of child ages at outcome assessment in ACEs and PSS subsamples | 31 |
| Figure S4. Dose-Response Relation Between Maternal Adverse Childhood Experiences (ACEs) and Child Total Problems Score | 32 |

**Supplemental Methods

*Measures***

**Maternal Adverse Childhood Experiences.** Maternal adverse childhood experiences (ACEs) exposure was assessed using two forms, both of which inquired about the same 10 items of ACEs. Using the primary version, mothers responded (1) ‘Yes’ or (0) ‘No’ to each of the 10 items. Using the alternate version, mothers responded (1) ‘Yes’ or (0) ‘No’ to items regarding physical neglect, emotional neglect, and abuse of their mother. Additionally, respondents were provided a list of statements conveying the remaining 7 types of adversity captured by the questionnaires: physical abuse, emotional abuse, sexual abuse, separation or divorce of parents, substance abuse by a household member, mental illness of a household member, and incarceration of a household member. Mothers then reported on the total number of these items they experienced during childhood, with a maximum of 5 or more. For both forms, all items were summed into a total count. When multiple questionnaires were administered, item-level maximums were calculated. Responses from the two versions were harmonized based on their overlapping responses. A pseudo-continuous total count of ACEs (0–5+) was calculated, mean-centered, and standardized for analyses.

**ECHO Cohorts**

The following ECHO Cohorts contributed data to the analyses. Additional information on each cohort is available on the ECHO website (<https://echochildren.org/>).

| ECHO in Puerto Rico (PROTECT) | Safe Passage Study (PASS) |
| --- | --- |
| Prematurity and Respiratory Outcomes Program (DINE-PROP) | Pregnancy Environment and Lifestyle Study (PETALS) |
| Trial of Late Surfactant (DINE-TOLSURF) | Microbes, Allergy, Asthma & Pets Study (MAAP) |
| NICU Hospital Exposures and Long-Term Health (DINE-NICUHEALTH) | Maternal and Development Risks from Environmental and Social Stressors (MADRES) |
| Preterm Erythropoietin Neuroprotection Trial (DINE-PENUT) | Pittsburgh Girls Study (PGS) |
| Family Life Project (FLP) | Early Growth and Development Study (EGDS-CI) |
| Healthy Start | Early Growth and Development Study (EGDS-CII) |
| BAMBAM | Early Parenting of Children (EPoCh) |
| MINNIE | Vitamin D Antenatal Asthma Reduction Trial (VDAART) |
| Boricua Youth Study (BYS) | University of California Davis- Baby Siblings Research Consortium (UCDavis-BSRC) |
| Atlanta ECHO Cohort of Emory University | O'Connor - Rochester |
| University of California- Markers of Autism Risk in Babies (MARBLES) | O'Connor - Magee |
| Early Autism Risk Longitudinal Investigation (EARLI) | Nulliparous Pregnancy Outcomes Study Monitoring Mothers-to-be (NuMoM2b-Utah) |
| Project Viva | The NYU Children's Health and Environment Study (NYU CHES) |
| Michigan Archive for Research in Child Health (MARCH) | PRogramming of Intergenerational Stress Mechanisms (PRISM) |
| Illinois Kids Development Study (IKIDS) | Inova Childhood Longitudinal Study (ICLS) |
| Chemicals in our Bodies (CIOB) | National Children's Study - IVS Salt Lake County (NCS-IVS) |

**Supplemental Statistical Analyses**

The following are the equations used for the main models in our analyses:

Model 1 (minimally adjusted): CBCLij ~ β0 + β1*Maternal Childhood Predictor + uj + eij

Model 2 (fully adjusted): CBCLij ~ β0 + β1*Maternal Childhood Predictor + β2*Household income + β3*Maternal Education + β4*Maternal age at delivery + β5*Maternal Marital Status + β6*Child Sex + β7*Child age at CBCL + uj + eij

• CBCLij is the response for the ith participant of the jth cohort site

• β0 is the fixed intercept for the regression model

• β1 - β7 are the fixed slopes for the regression model

• uj is the random intercept for the jth cohort site

• eij is the error term

**Table S1A**

*Correlations Between Study Variables, PSS Subsample (N=4,441)*

|  | 1 | 2 | 3 | 4 | 5 | 6 | 7 | 8 | 9 | 10 | 11 | 12 | 13 | 14 |
| --- | --- | --- | --- | --- | --- | --- | --- | --- | --- | --- | --- | --- | --- | --- |
| 1. Child sex | 1 |  |  |  |  |  |  |  |  |  |  |  |  |  |
| 2. Child age at outcome | 0.01 | 1 |  |  |  |  |  |  |  |  |  |  |  |  |
| 3. Maternal depressive symptoms | -0.01 | -0.18  *** | 1 |  |  |  |  |  |  |  |  |  |  |  |
| 4. Maternal age at delivery | -0.03 | 0.02 | 0.01 | 1 |  |  |  |  |  |  |  |  |  |  |
| 5. Child gestational age at birth | 0.02 | 0.05  *** | -0.04  *** | 0.02 | 1 |  |  |  |  |  |  |  |  |  |
| 6. Maternal prenatal alcohol use | 0.03 | 0.06  *** | -0.02 | 0.13  *** | 0.04  *** | 1 |  |  |  |  |  |  |  |  |
| 7. Maternal prenatal smoking | 0.02 | 0.01 | 0.03 | -0.1  *** | -0.05  *** | 0.06  *** | 1 |  |  |  |  |  |  |  |
| 8. Maternal marital status | -0.01 | 0.02 | 0.03  *** | -0.26  *** | -0.07  *** | -0.04  *** | 0.15  *** | 1 |  |  |  |  |  |  |
| 9. Maternal education | -0.03 | 0.10  *** | -0.03  *** | 0.40  *** | 0.09  *** | 0.17  *** | -0.16  *** | -0.31  *** | 1 |  |  |  |  |  |
| 10. Household income | -0.03 | 0.24  *** | -0.15  *** | 0.42  *** | 0.15  *** | 0.15  *** | -0.17  *** | -0.42  *** | 0.60  *** | 1 |  |  |  |  |
| 11. Maternal prenatal PSS | 0.01 | -0.03 | 0.31  *** | -0.06  *** | -0.04  *** | 0.01 | 0.05  *** | 0.13  *** | -0.07  *** | -0.18  *** | 1 |  |  |  |
| 12. Total problems | -0.07  *** | 0.06  *** | 0.22  *** | -0.07  *** | -0.04  *** | 0.01 | 0.06  *** | 0.09  *** | -0.12  *** | -0.13  *** | 0.22  *** | 1 |  |  |
| 13. Internalizing problems | -0.05  *** | 0.16  *** | 0.18  *** | -0.07  *** | -0.02 | 0.00 | 0.05  *** | 0.10  *** | -0.09  *** | -0.09  *** | 0.19  *** | 0.84  *** | 1 |  |
| 14. Externalizing problems | -0.09  *** | 0.06  *** | 0.18  *** | -0.05  *** | -0.05  *** | 0.02 | 0.06  *** | 0.07  *** | -0.11  *** | -0.11  *** | 0.20  *** | 0.89  *** | 0.64  *** | 1 |
| Sample size per variable | 4441 | 4441 | 4069 | 4440 | 4441 | 3629 | 3881 | 4267 | 4398 | 3890 | 4441 | 4437 | 4438 | 4440 |

*Note*. PSS=Perceived Stress Scale.

*p<0.05

**p<0.01

***p<0.001

**Table S1B**

*Correlations Between Study Variables, ACEs Population (N=2,906)*

|  | 1 | 2 | 3 | 4 | 5 | 6 | 7 | 8 | 9 | 10 | 11 | 12 | 13 | 14 |
| --- | --- | --- | --- | --- | --- | --- | --- | --- | --- | --- | --- | --- | --- | --- |
| 1. Child sex | 1 |  |  |  |  |  |  |  |  |  |  |  |  |  |
| 2. Child age at outcome | -0.01 | 1 |  |  |  |  |  |  |  |  |  |  |  |  |
| 3. Maternal depressive symptoms | 0.01 | 0.22  *** | 1 |  |  |  |  |  |  |  |  |  |  |  |
| 4. Maternal age at delivery | -0.01 | -0.17  *** | -0.15  *** | 1 |  |  |  |  |  |  |  |  |  |  |
| 5. Child gestational age at birth | 0.03 | -0.04  *** | -0.02 | 0.00 | 1 |  |  |  |  |  |  |  |  |  |
| 6. Maternal prenatal alcohol use | 0.00 | 0.07  *** | -0.01 | 0.13  *** | 0.11  *** | 1 |  |  |  |  |  |  |  |  |
| 7. Maternal prenatal smoking | 0.00 | 0.07  *** | 0.08  *** | -0.13  *** | -0.01 | 0.02 | 1 |  |  |  |  |  |  |  |
| 8. Maternal marital status | 0.01 | 0.14  *** | 0.14  *** | -0.28  *** | -0.08  *** | -0.02 | 0.2  *** | 1 |  |  |  |  |  |  |
| 9. Maternal education | -0.03 | -0.04  *** | -0.15  *** | 0.38  *** | 0.06  *** | 0.07  *** | -0.19  *** | -0.26  *** | 1 |  |  |  |  |  |
| 10. Household income | -0.01 | 0.05  *** | -0.21  *** | 0.44  *** | 0.12  *** | 0.14  *** | -0.2  *** | -0.45  *** | 0.55  *** | 1 |  |  |  |  |
| 11. Maternal ACEs | 0.00 | 0.07  *** | 0.24  *** | -0.11  *** | -0.02 | 0.04 | 0.13  *** | 0.16  *** | -0.12  *** | -0.15  *** | 1 |  |  |  |
| 12. Total problems | -0.07  *** | 0.06  *** | 0.23  *** | -0.10  *** | -0.11  *** | 0.01 | 0.04 | 0.12  *** | -0.1  *** | -0.16  *** | 0.21  *** | 1 |  |  |
| 13. Internalizing problems | -0.03 | 0.14  *** | 0.24  *** | -0.08  *** | -0.10  *** | 0.01 | 0.03 | 0.10  *** | -0.07  *** | -0.11  *** | 0.19  *** | 0.80  4*** | 1 |  |
| 14. Externalizing problems | -0.10  *** | 0.06  *** | 0.14  *** | -0.07  *** | -0.09  *** | 0.02 | 0.03 | 0.07  *** | -0.09  *** | -0.10  *** | 0.17  *** | 0.87  *** | 0.60  *** | 1 |
| Sample size per variable | 2906 | 2906 | 2796 | 2893 | 2829 | 1868 | 1754 | 2845 | 2891 | 2691 | 2906 | 2906 | 2665 | 2739 |

*Note*. ACEs=adverse childhood experiences.
*p<0.05
**p<0.01
***p<0.001

**Table S1C**

*Correlations Between Study Variables, ACEs+PSS Population (N=834)*

|  | 1 | 2 | 3 | 4 | 5 | 6 | 7 | 8 | 9 | 10 | 11 | 12 | 13 | 14 | 15 |
| --- | --- | --- | --- | --- | --- | --- | --- | --- | --- | --- | --- | --- | --- | --- | --- |
| 1. Child sex | 1 |  |  |  |  |  |  |  |  |  |  |  |  |  |  |
| 2. Child age at outcome | -0.02 | 1 |  |  |  |  |  |  |  |  |  |  |  |  |  |
| 3. Maternal depressive symptoms | 0.03 | -0.24  *** | 1 |  |  |  |  |  |  |  |  |  |  |  |  |
| 4. Maternal age at delivery | -0.05 | 0.13  *** | -0.03 | 1 |  |  |  |  |  |  |  |  |  |  |  |
| 5. Child gestational age at birth | 0.02 | -0.05 | -0.03 | 0.00 | 1 |  |  |  |  |  |  |  |  |  |  |
| 6. Maternal prenatal alcohol use | 0.04 | 0.18  *** | -0.05 | 0.16  *** | 0.06 | 1 |  |  |  |  |  |  |  |  |  |
| 7. Maternal prenatal smoking | 0.03 | 0.01 | -0.01 | -0.01 | -0.01 | 0.06 | 1 |  |  |  |  |  |  |  |  |
| 8. Maternal marital status | 0.04 | -0.04 | 0.07  *** | -0.14  *** | -0.05 | -0.04 | 0.11  *** | 1 |  |  |  |  |  |  |  |
| 9. Maternal education | -0.05 | 0.36  *** | -0.16  *** | 0.23  *** | 0.13  *** | 0.19  *** | 0.02 | -0.11  *** | 1 |  |  |  |  |  |  |
| 10. Household income | -0.07  *** | 0.39  *** | -0.22  *** | 0.36  *** | 0.14  *** | 0.26  *** | -0.09  *** | -0.32  *** | 0.59  *** | 1 |  |  |  |  |  |
| 11. Maternal ACEs | -0.06 | 0.01 | 0.23  *** | -0.03 | -0.03 | 0.02 | 0.09  *** | 0.18  *** | -0.07  *** | -0.11  *** | 1 |  |  |  |  |
| 12. Maternal prenatal PSS | 0.05 | 0.19  *** | 0.19  *** | 0.03 | -0.06 | 0.11  *** | 0.09  *** | 0.12  *** | 0.05 | 0.04 | 0.22  *** | 1 |  |  |  |
| 13. Total problems | -0.09  *** | 0.03 | 0.31  *** | -0.07  *** | -0.10  *** | 0.01 | 0.05 | 0.13  *** | -0.12  *** | -0.12  *** | 0.24  *** | 0.20  *** | 1 |  |  |
| 14. Internalizing problems | -0.02 | 0.12  *** | 0.27  *** | -0.08  *** | -0.07  *** | 0.01 | 0.00 | 0.13  *** | -0.06 | -0.10  *** | 0.19  *** | 0.22  *** | 0.81  *** | 1 |  |
| 15. Externalizing problems | -0.09  *** | 0.09  *** | 0.21  *** | -0.07  *** | -0.13  *** | 0.01 | 0.05 | 0.04 | -0.11  *** | -0.08  *** | 0.18  *** | 0.16  *** | 0.85  *** | 0.58  *** | 1 |
| Sample size per variable | 834 | 834 | 825 | 833 | 834 | 582 | 581 | 818 | 833 | 771 | 834 | 834 | 834 | 834 | 834 |

*Note*. ACEs=adverse childhood experiences; PSS=Perceived Stress Scale.
*p<0.05
**p<0.01
***p<0.001

**Table S2**

*Regression Models of Maternal Adverse Childhood Experiences (ACEs) and Child Total Problems Score (Including Age and Sex Moderation and On-The-Path Adjustment), N=2,906*

| Model | Predictor | *B* | 95% CI | *P* |
| --- | --- | --- | --- | --- |
| Model 1 | Childhood predictor |  |  |  |
| Minimally adjusted^a^ | Standardized maternal ACEs | 2.78 | [2.34, 3.21] | <0.0001 |
| Model 2a | Childhood predictor |  |  |  |
| Fully adjusted^b^ | Standardized maternal ACEs | 2.53 | [2.09, 2.96] | <0.0001 |
|  | Covariate |  |  |  |
|  | Household income |  |  |  |
|  | $30,000–$49,999 | -0.77 | [-2.28, 0.73] | 0.31 |
|  | $50,000–$74,999 | -2.61 | [-4.23, -0.98] | 0.002 |
|  | $75,000–$99,999 | -3.04 | [-4.92, -1.17] | 0.001 |
|  | $100,000–$199,999 | -3.63 | [-5.32, -1.94] | <0.0001 |
|  | $200,000 or more | -3.98 | [-6.08, -1.89] | <0.001 |
|  | Maternal education |  |  |  |
|  | High school degree or equivalent | -2.78 | [-4.73, -0.84] | 0.005 |
|  | Some college, no degree | -2.09 | [-3.98, -0.20] | 0.03 |
|  | Bachelor’s degree | -1.84 | [-3.88, 0.20] | 0.08 |
|  | Advanced degree and above | -2.52 | [-4.6, -0.43] | 0.02 |
|  | Maternal age at delivery | -0.07 | [-0.16, 0.01] | 0.09 |
|  | Maternal marital status (single) | 1.15 | [-0.03, 2.32] | 0.06 |
|  | Child sex (female) | -1.80 | [-2.65, -0.96] | <0.0001 |
|  | Child age at outcome | -0.33 | [-0.51, -0.15] | <0.001 |
| Model 2b | Moderator |  |  |  |
| Sex moderation | Standardized maternal ACEs x child sex (female) | -0.29 | [-1.14, 0.55] | 0.49 |
| Model 2c | Moderator |  |  |  |
| Age moderation | Standardized maternal ACEs x child age at outcome | 0.06 | [-0.02, 0.15] | 0.16 |
| Model 3 | Childhood predictor |  |  |  |
| On the path^c^ | Standardized maternal ACEs | 1.90 | [1.46, 2.35] | <0.0001 |
|  | Covariate |  |  |  |
|  | Household income |  |  |  |
|  | $30,000–$49,999 | -0.55 | [-2.03, 0.93] | 0.47 |
|  | $50,000–$74,999 | -2.17 | [-3.77, -0.57] | 0.008 |
|  | $75,000–$99,999 | -2.89 | [-4.74, -1.05] | 0.002 |
|  | $100,000–$199,999 | -3.14 | [-4.81, -1.47] | <0.001 |
|  | $200,000 or more | -3.24 | [-5.31, -1.17] | 0.002 |
|  | Maternal education |  |  |  |
|  | High school degree or equivalent | -2.90 | [-4.81, -0.99] | 0.003 |
|  | Some college, no degree | -2.42 | [-4.30, -0.55] | 0.01 |
|  | Bachelor’s degree | -2.23 | [-4.28, -0.17] | 0.03 |
|  | Advanced degree and above | -2.81 | [-4.89, -0.73] | 0.008 |
|  | Maternal age at delivery | -0.10 | [-0.18, -0.02] | 0.02 |
|  | Maternal marital status (single) | 0.90 | [-0.26, 2.06] | 0.13 |
|  | Child sex (female) | -1.87 | [-2.69, -1.04] | <0.0001 |
|  | Child age at outcome | -0.49 | [-0.68, -0.31] | <0.0001 |
|  | Prenatal smoking | -0.02 | [-2.59, 2.56] | 0.99 |
|  | Any prenatal alcohol use | 0.68 | [-1.00, 2.36] | 0.43 |
|  | Child gestational age at birth | -0.39 | [-0.59, -0.19] | <0.001 |
|  | Maternal depressive symptoms | 0.31 | [0.25, 0.37] | <0.0001 |

*Note*. ECHO cohort included as random intercept in all models. *B* values are unstandardized beta coefficients. ACEs=adverse childhood experiences.
^a^Minimally adjusted model accounted for 16% of variance in child Total Problems score.
^b^Fully adjusted model accounted for 21% of variance in child Total Problems score.
^c^On-the-path model accounted for 24% of variance in child Total Problems score.

**Table S3**

*Regression Models of Maternal Prenatal Stress and Child Total Problems Score (Including Age and Sex Moderation and On-The-Path Adjustment), N=4,437*

| Model | Predictor | *B* | 95% CI | *P* |
| --- | --- | --- | --- | --- |
| Model 1 | Prenatal predictor |  |  |  |
| Minimally adjusted^a^ | Standardized prenatal PSS | 2.51 | [2.18, 2.83] | <0.0001 |
| Model 2a | Prenatal predictor |  |  |  |
| Fully adjusted^b^ | Standardized prenatal PSS | 2.36 | [2.03, 2.68] | <0.0001 |
|  | Covariate |  |  |  |
|  | Household income |  |  |  |
|  | $30,000–$49,999 | -1.25 | [-2.46, -0.04] | 0.04 |
|  | $50,000–$74,999 | -1.03 | [-2.27, 0.21] | 0.10 |
|  | $75,000–$99,999 | -1.36 | [-2.82, 0.11] | 0.07 |
|  | $100,000–$199,999 | -2.18 | [-3.56, -0.79] | 0.002 |
|  | $200,000 or more | -2.14 | [-3.75, -0.52] | 0.01 |
|  | Maternal education |  |  |  |
|  | High school degree or equivalent | -1.89 | [-3.48, -0.29] | 0.02 |
|  | Some college, no degree | -1.59 | [-3.11, -0.08] | 0.04 |
|  | Bachelor’s degree | -1.99 | [-3.61, -0.37] | 0.02 |
|  | Advanced degree and above | -2.83 | [-4.51, -1.14] | 0.001 |
|  | Maternal age at delivery | -0.02 | [-0.08, 0.05] | 0.62 |
|  | Maternal marital status |  |  |  |
|  | Single | 0.76 | [-0.19, 1.70] | 0.12 |
|  | Child sex |  |  |  |
|  | Female | -1.88 | [-2.50, -1.26] | <0.0001 |
|  | Child age at outcome | 0.26 | [0.09, 0.44] | 0.004 |
| Model 2b | Moderator |  |  |  |
| Sex moderation | Standardized prenatal PSS x child sex (female) | -0.42 | [-1.04, 0.19] | 0.18 |
| Model 2c | Moderator |  |  |  |
| Age moderation | Standardized prenatal PSS x child age at outcome | -0.03 | [-0.15, 0.09] | 0.61 |
| Model 3 | Prenatal predictor |  |  |  |
| On the path^c^ | Standardized prenatal PSS | 1.55 | [1.21, 1.89] | <0.0001 |
|  | Covariate |  |  |  |
|  | Household income |  |  |  |
|  | $30,000–$49,999 | -1.07 | [-2.25, 0.12] | 0.08 |
|  | $50,000–$74,999 | -0.66 | [-1.87, 0.56] | 0.29 |
|  | $75,000–$99,999 | -1.08 | [-2.50, 0.34] | 0.14 |
|  | $100,000–$199,999 | -1.71 | [-3.08, -0.34] | 0.01 |
|  | $200,000 or more | -1.74 | [-3.35, -0.13] | 0.03 |
|  | Maternal education |  |  |  |
|  | High school degree or equivalent | -1.72 | [-3.28, -0.17] | 0.03 |
|  | Some college, no degree | -1.70 | [-3.18, -0.22] | 0.02 |
|  | Bachelor’s degree | -2.15 | [-3.74, -0.56] | 0.008 |
|  | Advanced degree and above | -3.01 | [-4.66, -1.36] | <0.001 |
|  | Maternal age at delivery | -0.04 | [-0.10, 0.03] | 0.27 |
|  | Maternal marital status |  |  |  |
|  | Single | 0.46 | [-0.46, 1.39] | 0.33 |
|  | Child sex |  |  |  |
|  | Female | -1.79 | [-2.4, -1.18] | <0.0001 |
|  | Child age at outcome | 0.21 | [0.03, 0.39] | 0.02 |
|  | Prenatal smoking | 0.13 | [-1.57, 1.84] | 0.88 |
|  | Any prenatal alcohol use | 0.87 | [0.00, 1.74] | 0.05 |
|  | Child gestational age at birth | -0.20 | [-0.37, -0.04] | 0.02 |
|  | Maternal depressive symptoms | 0.27 | [0.23, 0.31] | <0.0001 |

*Note*. ECHO cohort included as random intercept in all models. *B* values are unstandardized beta coefficients. PSS=Perceived Stress Scale.
^a^Minimally adjusted model accounted for 10% of variance in child Total Problems score.
^b^Fully adjusted model accounted for 11% of variance in child Total Problems score.
^c^On-the-path model accounted for 16% of variance in child Total Problems score.

**Table S4**

*Regression Models of Maternal Adverse Childhood Experiences (ACEs), Maternal Prenatal Stress, and Child Total Problems Score (Including Age and Sex Moderation and On-The-Path Adjustment), N=834*

| Model | Predictor | *B* | 95% CI | *P* |
| --- | --- | --- | --- | --- |
| Model 1 | Childhood predictor |  |  |  |
| Minimally adjusted^a^ | Standardized maternal ACEs | 2.03 | [1.28, 2.78] | <0.0001 |
|  | Prenatal predictor |  |  |  |
|  | Standardized prenatal PSS | 2.16 | [1.40, 2.92] | <0.0001 |
| Model 2a | Childhood predictor |  |  |  |
| Fully adjusted^b^ | Standardized maternal ACEs | 1.72 | [0.96, 2.48] | <0.0001 |
|  | Prenatal predictor |  |  |  |
|  | Standardized prenatal PSS | 2.05 | [1.29, 2.80] | <0.0001 |
|  | Covariate |  |  |  |
|  | Household income |  |  |  |
|  | $30,000–$49,999 | -2.57 | [-5.17, 0.03] | 0.05 |
|  | $50,000–$74,999 | -2.64 | [-5.55, 0.27] | 0.08 |
|  | $75,000–$99,999 | -2.99 | [-6.15, 0.17] | 0.06 |
|  | $100,000–$199,999 | -2.69 | [-5.48, 0.11] | 0.06 |
|  | $200,000 or more | -2.91 | [-6.23, 0.40] | 0.08 |
|  | Maternal education |  |  |  |
|  | High school degree or equivalent | -2.85 | [-6.18, 0.48] | 0.09 |
|  | Some college, no degree | -1.19 | [-4.49, 2.10] | 0.48 |
|  | Bachelor’s degree | -1.75 | [-5.22, 1.73] | 0.32 |
|  | Advanced degree and above | -2.82 | [-6.33, 0.70] | 0.12 |
|  | Maternal age at delivery | -0.10 | [-0.25, 0.05] | 0.18 |
|  | Maternal marital status |  |  |  |
|  | Single | 2.41 | [0.32, 4.50] | 0.02 |
|  | Child sex |  |  |  |
|  | Female | -2.28 | [-3.72, -0.85] | 0.002 |
|  | Child age at outcome | 0.34 | [-0.06, 0.75] | 0.10 |
| Model 2bi | Moderator |  |  |  |
| Sex moderation | Standardized maternal ACEs x child sex (female) | -0.55 | [-1.98, 0.89] | 0.46 |
| Model 2bii | Moderator |  |  |  |
| Sex moderation | Standardized prenatal PSS x child sex (female) | 0.37 | [-1.07, 1.80] | 0.61 |
| Model 2ci | Moderator |  |  |  |
| Age moderation | Standardized maternal ACEs x child age at outcome | 0.08 | [-0.19, 0.34] | 0.58 |
| Model 2cii | Moderator |  |  |  |
| Age moderation | Standardized prenatal PSS x child age at outcome | -0.05 | [-0.31, 0.21] | 0.70 |
| Model 2d | Moderator |  |  |  |
| ACEs moderation | Standardized maternal ACEs x standardized prenatal PSS | 0.07 | [-0.69, 0.84] | 0.85 |
| Model 3 | Childhood predictor |  |  |  |
| On the path^c^ | Standardized maternal ACEs | 1.16 | [0.41, 1.90] | 0.002 |
|  | Prenatal predictor |  |  |  |
|  | Standardized prenatal PSS | 1.18 | [0.42, 1.93] | 0.002 |
|  | Covariate |  |  |  |
|  | Household income |  |  |  |
|  | $30,000–$49,999 | -2.11 | [-4.59, 0.37] | 0.10 |
|  | $50,000–$74,999 | -1.90 | [-4.68, 0.88] | 0.18 |
|  | $75,000–$99,999 | -2.92 | [-5.95, 0.11] | 0.06 |
|  | $100,000–$199,999 | -2.05 | [-4.76, 0.65] | 0.14 |
|  | $200,000 or more | -1.81 | [-5.04, 1.43] | 0.27 |
|  | Maternal education |  |  |  |
|  | High school degree or equivalent | -3.58 | [-6.79, -0.37] | 0.03 |
|  | Some college, no degree | -1.74 | [-4.93, 1.45] | 0.28 |
|  | Bachelor’s degree | -2.60 | [-5.93, 0.74] | 0.13 |
|  | Advanced degree and above | -3.73 | [-7.12, -0.33] | 0.03 |
|  | Maternal age at delivery | -0.13 | [-0.27, 0.02] | 0.09 |
|  | Maternal marital status (single) | 2.11 | [0.09, 4.13] | 0.04 |
|  | Child sex (female) | -2.26 | [-3.65, -0.87] | 0.001 |
|  | Child age at outcome | 0.38 | [0.01, 0.75] | 0.05 |
|  | Prenatal smoking | 1.77 | [-3.38, 6.93] | 0.50 |
|  | Any prenatal alcohol use | 0.59 | [-1.72, 2.90] | 0.62 |
|  | Child gestational age at birth | -0.55 | [-0.97, -0.14] | 0.009 |
|  | Maternal depressive symptoms | 0.37 | [0.28, 0.46] | <0.0001 |
| *Note*. ECHO cohort included as random intercept in all models. *B* values are unstandardized beta coefficients. ACEs=adverse childhood experiences; ECHO=Environmental influences on Child Health Outcomes; PSS=Perceived Stress Scale. | | | | |
| ^a^Minimally adjusted model accounted for 11% of variance in child Total Problems score. | | | | |
| ^b^Fully adjusted model accounted for 15% of variance in child Total Problems score. | | | | |
| ^c^On-the-path model accounted for 22% of variance in child Total Problems score. | | | | |
|  | | | | |

**Table S5**

*Regression Models of Maternal Adverse Childhood Experiences (ACEs) and Child Externalizing Problems Score, N=2,729*

| Model | Predictor | *B* | 95% CI | *p* |
| --- | --- | --- | --- | --- |
| Model 1 | Childhood predictor |  |  |  |
| Minimally adjusted^a^ | Standardized maternal ACEs | 2.00 | [1.57, 2.42] | <0.0001 |
| Model 2a | Childhood predictor |  |  |  |
| Fully adjusted^b^ | Standardized maternal ACEs | 1.84 | [1.42, 2.27] | <0.0001 |
|  | Covariate |  |  |  |
|  | Household income |  |  |  |
|  | $30,000–$49,999 | -0.46 | [-1.90, 0.98] | 0.53 |
|  | $50,000–$74,999 | -2.07 | [-3.64, -0.50] | 0.01 |
|  | $75,000–$99,999 | -1.65 | [-3.47, 0.18] | 0.08 |
|  | $100,000–$199,999 | -1.65 | [-3.32, 0.02] | 0.05 |
|  | $200,000 or more | -2.09 | [-4.12, -0.05] | 0.04 |
|  | Maternal education |  |  |  |
|  | High school degree or equivalent | -2.59 | [-4.45, -0.74] | 0.006 |
|  | Some college, no degree | -1.95 | [-3.76, -0.14] | 0.04 |
|  | Bachelor’s degree | -1.86 | [-3.82, 0.09] | 0.06 |
|  | Advanced degree and above | -2.69 | [-4.71, -0.68] | 0.009 |
|  | Maternal age at delivery | -0.10 | [-0.18, -0.02] | 0.02 |
|  | Maternal marital status (single) | 1.18 | [0.05, 2.31] | 0.04 |
|  | Child sex (female) | -2.65 | [-3.47, -1.82] | <0.0001 |
|  | Child age at CBCL | -0.44 | [-0.61, -0.26] | <0.0001 |
| Model 2b | Moderator |  |  |  |
| Sex moderation | Standardized maternal ACEs x child sex (female) | -0.74 | [-1.56, 0.08] | 0.08 |
| Model 2c | Moderator |  |  |  |
| Age moderation | Standardized maternal ACEs x child age at CBCL | 0.01 | [-0.07, 0.09] | 0.80 |
| Model 3 | Childhood predictor |  |  |  |
| On the path^c^ | Standardized maternal ACEs | 1.44 | [1.00, 1.88] | <0.0001 |
|  | Covariate |  |  |  |
|  | Household income |  |  |  |
|  | $30,000–$49,999 | -0.31 | [-1.74, 1.11] | 0.67 |
|  | $50,000–$74,999 | -1.78 | [-3.34, -0.22] | 0.03 |
|  | $75,000–$99,999 | -1.56 | [-3.37, 0.25] | 0.09 |
|  | $100,000–$199,999 | -1.35 | [-3.03, 0.33] | 0.12 |
|  | $200,000 or more | -1.57 | [-3.61, 0.47] | 0.13 |
|  | Maternal education |  |  |  |
|  | High school degree or equivalent | -2.64 | [-4.49, -0.80] | 0.005 |
|  | Some college, no degree | -2.13 | [-3.94, -0.32] | 0.02 |
|  | Bachelor’s degree | -2.04 | [-4.02, -0.06] | 0.04 |
|  | Advanced degree and above | -2.82 | [-4.85, -0.79] | 0.007 |
|  | Maternal age at delivery | -0.12 | [-0.20, -0.04] | 0.004 |
|  | Maternal marital status (single) | 0.98 | [-0.15, 2.10] | 0.09 |
|  | Child sex (female) | -2.69 | [-3.51, -1.87] | <0.0001 |
|  | Child age at CBCL | -0.55 | [-0.73, -0.37] | <0.0001 |
|  | Prenatal smoking | 0.43 | [-1.91, 2.77] | 0.72 |
|  | Any prenatal alcohol use | 0.81 | [-0.82, 2.44] | 0.33 |
|  | Child gestational age at birth | -0.29 | [-0.49, -0.09] | 0.005 |
|  | Maternal depressive symptoms | 0.20 | [0.14, 0.25] | <0.0001 |

*Note*. ECHO cohort included as random intercept in all models. *B* values are unstandardized beta coefficients. ACEs=adverse childhood experiences; CBCL=Child Behavior Checklist; ECHO=Environmental influences on Child Health Outcomes.

^a^Minimally adjusted model accounted for 15% of variance in child Externalizing Problems score.

^b^Fully adjusted model accounted for 23% of variance in child Externalizing Problems score.

^c^On-the-path model accounted for 24% of variance in child Externalizing Problems score.

**Table S6**

*Regression Models of Maternal Prenatal Stress and Child Externalizing Problems Score, N=4,440*

| Model | Predictor | *B* | 95% CI | *P* |
| --- | --- | --- | --- | --- |
| Model 1 | Prenatal predictor |  |  |  |
| Minimally adjusted^a^ | Standardized prenatal PSS | 2.16 | [1.85, 2.47] | <0.0001 |
| Model 2a | Prenatal predictor |  |  |  |
| Fully adjusted^b^ | Standardized prenatal PSS | 2.05 | [1.74, 2.36] | <0.0001 |
|  | Covariate |  |  |  |
|  | Household income |  |  |  |
|  | $30,000–$49,999 | -0.23 | [-1.37, 0.91] | 0.70 |
|  | $50,000–$74,999 | -0.42 | [-1.61, 0.77] | 0.49 |
|  | $75,000–$99,999 | -0.61 | [-2.00, 0.79] | 0.39 |
|  | $100,000–$199,999 | -1.11 | [-2.44, 0.21] | 0.10 |
|  | $200,000 or more | -0.83 | [-2.37, 0.71] | 0.29 |
|  | Maternal education |  |  |  |
|  | High school degree or equivalent | -1.70 | [-3.22, -0.18] | 0.03 |
|  | Some college, no degree | -0.94 | [-2.39, 0.51] | 0.20 |
|  | Bachelor’s degree | -1.69 | [-3.24, -0.13] | 0.03 |
|  | Advanced degree and above | -2.64 | [-4.25, -1.02] | 0.001 |
|  | Maternal age at delivery | -0.01 | [-0.07, 0.06] | 0.81 |
|  | Maternal marital status (single) | 0.54 | [-0.37, 1.44] | 0.25 |
|  | Child sex (female) | -2.01 | [-2.6, -1.41] | <0.0001 |
|  | Child age at CBCL | 0.22 | [0.05, 0.4] | 0.01 |
| Model 2b | Moderator |  |  |  |
| Sex moderation | Standardized prenatal PSS x child sex (female) | -0.49 | [-1.09, 0.10] | 0.10 |
| Model 2c | Moderator |  |  |  |
| Age moderation | Standardized prenatal PSS x child age at CBCL | -0.06 | [-0.18, 0.06] | 0.31 |
| Model 3 | Prenatal predictor |  |  |  |
| On the path^c^ | Standardized prenatal PSS | 1.43 | [1.10, 1.75] | <0.0001 |
|  | Covariate |  |  |  |
|  | Household income |  |  |  |
|  | $30,000–$49,999 | -0.07 | [-1.19, 1.06] | 0.91 |
|  | $50,000–$74,999 | -0.10 | [-1.28, 1.08] | 0.87 |
|  | $75,000–$99,999 | -0.37 | [-1.74, 0.99] | 0.59 |
|  | $100,000–$199,999 | -0.72 | [-2.03, 0.60] | 0.28 |
|  | $200,000 or more | -0.50 | [-2.04, 1.04] | 0.53 |
|  | Maternal education |  |  |  |
|  | High school degree or equivalent | -1.56 | [-3.05, -0.06] | 0.04 |
|  | Some college, no degree | -1.01 | [-2.44, 0.42] | 0.16 |
|  | Bachelor’s degree | -1.80 | [-3.33, -0.26] | 0.02 |
|  | Advanced degree and above | -2.78 | [-4.37, -1.18] | <0.001 |
|  | Maternal age at delivery | -0.02 | [-0.09, 0.04] | 0.45 |
|  | Maternal marital status (single) | 0.30 | [-0.59, 1.19] | 0.51 |
|  | Child sex (female) | -1.94 | [-2.53, -1.35] | <0.0001 |
|  | Child age at CBCL | 0.19 | [0.02, 0.36] | 0.03 |
|  | Prenatal smoking | 0.46 | [-1.22, 2.13] | 0.59 |
|  | Any prenatal alcohol use | 0.79 | [-0.04, 1.62] | 0.06 |
|  | Child gestational age at birth | -0.22 | [-0.38, -0.05] | 0.01 |
|  | Maternal depressive symptoms | 0.21 | [0.17, 0.25] | <0.0001 |

*Note*. ECHO cohort included as random intercept in all models. *B* values are unstandardized beta coefficients. CBCL=Child Behavior Checklist; ECHO=Environmental influences on Child Health Outcomes; PSS=Perceived Stress Scale.

^a^Minimally adjusted model accounted for 10% of variance in child Externalizing Problems score.

^b^Fully adjusted model accounted for 11% of variance in child Externalizing Problems score.

^c^On-the-path model accounted for 14% of variance in child Externalizing Problems score.

**Table S7**

*Regression Models of Maternal Adverse Childhood Experiences (ACEs), Maternal Prenatal Stress, and Child Externalizing Problems Score, N=834*

| Model | Predictor | *B* | 95% CI | *P* |
| --- | --- | --- | --- | --- |
| Model 1 | Childhood predictor |  |  |  |
| Minimally adjusted^a^ | Standardized maternal ACEs | 1.38 | [0.71, 2.05] | <0.0001 |
|  | Prenatal predictor |  |  |  |
|  | Standardized prenatal PSS | 1.39 | [0.71, 2.08] | <0.0001 |
| Model 2a | Childhood predictor |  |  |  |
| Fully adjusted^b^ | Standardized maternal ACEs | 1.22 | [0.53, 1.91] | <0.001 |
|  | Prenatal predictor |  |  |  |
|  | Standardized prenatal PSS | 1.38 | [0.69, 2.06] | <0.0001 |
|  | Covariate |  |  |  |
|  | Household income |  |  |  |
|  | $30,000–$49,999 | -0.56 | [-2.95, 1.83] | 0.65 |
|  | $50,000–$74,999 | -1.19 | [-3.91, 1.53] | 0.39 |
|  | $75,000–$99,999 | -1.28 | [-4.18, 1.62] | 0.39 |
|  | $100,000–$199,999 | -0.39 | [-2.97, 2.20] | 0.77 |
|  | $200,000 or more | -0.51 | [-3.56, 2.54] | 0.74 |
|  | Maternal education |  |  |  |
|  | High school degree or equivalent | -1.98 | [-5.00, 1.04] | 0.20 |
|  | Some college, no degree | -0.56 | [-3.56, 2.43] | 0.71 |
|  | Bachelor’s degree | -1.56 | [-4.72, 1.61] | 0.34 |
|  | Advanced degree and above | -2.15 | [-5.36, 1.06] | 0.19 |
|  | Maternal age at delivery | -0.11 | [-0.25, 0.03] | 0.11 |
|  | Maternal marital status (single) | 1.26 | [-0.66, 3.17] | 0.20 |
|  | Child sex (female) | -2.03 | [-3.33, -0.73] | 0.002 |
|  | Child age at outcome | 0.23 | [-0.20, 0.67] | 0.30 |
| Model 2bi | Moderator |  |  |  |
| Sex moderation | Standardized maternal ACEs x child sex (female) | -0.76 | [-2.06, 0.54] | 0.25 |
| Model 2bii | Moderator |  |  |  |
| Sex moderation | Standardized prenatal PSS x child sex (female) | 0.59 | [-0.70, 1.89] | 0.37 |
| Model 2ci | Moderator |  |  |  |
| Age moderation | Standardized maternal ACEs x child age at outcome | -0.04 | [-0.28, 0.19] | 0.72 |
| Model 2cii | Moderator |  |  |  |
| Age moderation | Standardized prenatal PSS x child age at outcome | -0.05 | [-0.31, 0.21] | 0.70 |
| Model 2d | Moderator |  |  |  |
| ACEs moderation | Standardized maternal ACEs x standardized prenatal PSS | -0.37 | [-1.07, 0.32] | 0.29 |
| Model 3 | Childhood predictor |  |  |  |
| On the path^c^ | Standardized maternal ACEs | 0.80 | [0.12, 1.49] | 0.02 |
|  | Prenatal predictor |  |  |  |
|  | Standardized prenatal PSS | 0.71 | [0.01, 1.41] | 0.05 |
|  | Covariate |  |  |  |
|  | Household income |  |  |  |
|  | $30,000–$49,999 | -0.21 | [-2.53, 2.11] | 0.86 |
|  | $50,000–$74,999 | -0.71 | [-3.35, 1.92] | 0.60 |
|  | $75,000–$99,999 | -1.32 | [-4.15, 1.50] | 0.36 |
|  | $100,000–$199,999 | 0.05 | [-2.49, 2.59] | 0.97 |
|  | $200,000 or more | 0.22 | [-2.79, 3.24] | 0.89 |
|  | Maternal education |  |  |  |
|  | High school degree or equivalent | -2.61 | [-5.56, 0.34] | 0.08 |
|  | Some college, no degree | -1.11 | [-4.06, 1.84] | 0.46 |
|  | Bachelor’s degree | -2.33 | [-5.43, 0.78] | 0.14 |
|  | Advanced degree and above | -2.94 | [-6.11, 0.22] | 0.07 |
|  | Maternal age at delivery | -0.13 | [-0.26, 0.01] | 0.06 |
|  | Maternal marital status (single) | 0.91 | [-0.97, 2.78] | 0.34 |
|  | Child sex (female) | -1.97 | [-3.24, -0.7] | 0.002 |
|  | Child age at outcome | 0.15 | [-0.28, 0.58] | 0.50 |
|  | Prenatal smoking | 2.06 | [-1.99, 6.11] | 0.32 |
|  | Any prenatal alcohol use | 0.21 | [-1.83, 2.25] | 0.84 |
|  | Child gestational age at birth | -0.54 | [-0.92, -0.17] | 0.005 |
|  | Maternal depressive symptoms | 0.27 | [0.18, 0.35] | <0.0001 |

*Note*. ECHO cohort included as random intercept in all models. *B* values are unstandardized beta coefficients. ACEs=adverse childhood experiences; CBCL=Child Behavior Checklist; ECHO=Environmental influences on Child Health Outcomes; PSS=Perceived Stress Scale.

^a^Minimally adjusted model accounted for 13% of variance in child Externalizing Problems score.

^b^Fully adjusted model accounted for 14% of variance in child Externalizing Problems score.

^c^On-the-path model accounted for 19% of variance in child Externalizing Problems score.

**Table S8**

*Regression Models of Maternal Adverse Childhood Experiences (ACEs) and Child Internalizing Problems Score, N=2,665*

| Model | Predictor | *B* | 95% CI | *P* |
| --- | --- | --- | --- | --- |
| Model 1 | Childhood predictor |  |  |  |
| Minimally adjusted^a^ | Standardized maternal ACEs | 2.28 | [1.86, 2.71] | <0.0001 |
| Model 2a | Childhood predictor |  |  |  |
| Fully adjusted^b^ | Standardized maternal ACEs | 2.06 | [1.63, 2.49] | <0.0001 |
|  | Covariate |  |  |  |
|  | Household income |  |  |  |
|  | $30,000–$49,999 | -0.86 | [-2.30, 0.57] | 0.24 |
|  | $50,000–$74,999 | -2.98 | [-4.55, -1.41] | <0.001 |
|  | $75,000–$99,999 | -3.35 | [-5.15, -1.55] | <0.001 |
|  | $100,000–$199,999 | -3.49 | [-5.15, -1.83] | <0.0001 |
|  | $200,000 or more | -4.67 | [-6.75, -2.59] | <0.0001 |
|  | Maternal education |  |  |  |
|  | High school degree or equivalent | -2.62 | [-4.48, -0.76] | 0.006 |
|  | Some college, no degree | -1.84 | [-3.65, -0.03] | 0.05 |
|  | Bachelor’s degree | -1.26 | [-3.21, 0.69] | 0.20 |
|  | Advanced degree and above | -1.54 | [-3.56, 0.49] | 0.14 |
|  | Maternal age at delivery | -0.02 | [-0.11, 0.06] | 0.59 |
|  | Maternal marital status (single) | 0.73 | [-0.40, 1.87] | 0.20 |
|  | Child sex (female) | -0.76 | [-1.60, 0.08] | 0.07 |
|  | Child age at outcome | 0.12 | [-0.06, 0.29] | 0.18 |
| Model 2b | Moderator |  |  |  |
| Sex moderation | Standardized maternal ACEs x child sex (female) | 0.08 | [-0.75, 0.91] | 0.85 |
| Model 2c | Moderator |  |  |  |
| Age moderation | Standardized maternal ACEs x child age at outcome | 0.07 | [-0.01, 0.15] | 0.09 |
| Model 3 | Childhood predictor |  |  |  |
| On the path^c^ | Standardized maternal ACEs | 1.42 | [0.99, 1.86] | <0.0001 |
|  | Covariate |  |  |  |
|  | Household income |  |  |  |
|  | $30,000–$49,999 | -0.59 | [-2.00, 0.81] | 0.41 |
|  | $50,000–$74,999 | -2.53 | [-4.08, -0.99] | 0.001 |
|  | $75,000–$99,999 | -3.19 | [-4.96, -1.43] | <0.001 |
|  | $100,000–$199,999 | -2.95 | [-4.60, -1.31] | <0.001 |
|  | $200,000 or more | -3.73 | [-5.79, -1.66] | <0.001 |
|  | Maternal education |  |  |  |
|  | High school degree or equivalent | -2.84 | [-4.66, -1.01] | 0.002 |
|  | Some college, no degree | -2.22 | [-4.01, -0.43] | 0.01 |
|  | Bachelor’s degree | -1.76 | [-3.71, 0.19] | 0.08 |
|  | Advanced degree and above | -1.99 | [-4.01, 0.02] | 0.05 |
|  | Maternal age at delivery | -0.05 | [-0.13, 0.03] | 0.21 |
|  | Maternal marital status (single) | 0.55 | [-0.56, 1.66] | 0.33 |
|  | Child sex (female) | -0.80 | [-1.62, 0.02] | 0.05 |
|  | Child age at outcome | -0.06 | [-0.24, 0.11] | 0.47 |
|  | Prenatal smoking | -0.22 | [-2.65, 2.21] | 0.86 |
|  | Any prenatal alcohol use | 0.39 | [-1.48, 2.26] | 0.68 |
|  | Child gestational age at birth | -0.31 | [-0.51, -0.10] | 0.003 |
|  | Maternal depressive symptoms | 0.32 | [0.26, 0.38] | <0.0001 |

*Note*. ECHO cohort included as random intercept in all models. *B* values are unstandardized beta coefficients. ACEs=adverse childhood experiences; CBCL=Child Behavior Checklist; ECHO=Environmental influences on Child Health Outcomes.

^a^Minimally adjusted model accounted for 18% of variance in child Internalizing Problems score.

^b^Fully Adjusted model accounted for 22% of variance in child Internalizing Problems score.

^c^On-the-path model accounted for 25% of variance in child Internalizing Problems score.

**Table S9**

*Regression Models of Maternal Prenatal Stress and Child Internalizing Problems Score, N=4,438*

| Model | Predictor | *B* | 95% CI | *P* |
| --- | --- | --- | --- | --- |
| Model 1 | Prenatal predictor |  |  |  |
| Minimally adjusted^a^ | Standardized prenatal PSS | 2.08 | [1.76, 2.4] | <0.0001 |
| Model 2a | Prenatal predictor |  |  |  |
| Fully adjusted^b^ | Standardized prenatal PSS | 1.94 | [1.62, 2.26] | <0.0001 |
|  | Covariate |  |  |  |
|  | Household income |  |  |  |
|  | $30,000–$49,999 | -1.44 | [-2.64, -0.24] | 0.02 |
|  | $50,000–$74,999 | -1.85 | [-3.07, -0.63] | 0.003 |
|  | $75,000–$99,999 | -1.95 | [-3.38, -0.52] | 0.008 |
|  | $100,000–$199,999 | -2.72 | [-4.07, -1.37] | <0.0001 |
|  | $200,000 or more | -2.95 | [-4.54, -1.36] | <0.001 |
|  | Maternal education |  |  |  |
|  | High school degree or equivalent | -2.15 | [-3.73, -0.57] | 0.008 |
|  | Some college, no degree | -2.39 | [-3.89, -0.89] | 0.002 |
|  | Bachelor’s degree | -1.90 | [-3.50, -0.30] | 0.02 |
|  | Advanced degree and above | -2.36 | [-4.03, -0.69] | 0.006 |
|  | Maternal age at delivery | -0.04 | [-0.11, 0.03] | 0.24 |
|  | Maternal marital status (single) | 0.83 | [-0.10, 1.75] | 0.08 |
|  | Child sex (female) | -1.40 | [-2.01, -0.79] | <0.0001 |
|  | Child age at outcome | 0.66 | [0.48, 0.83] | <0.0001 |
| Model 2b | Moderator |  |  |  |
| Sex moderation | Standardized prenatal PSS x child sex (female) | -0.31 | [-0.92, 0.31] | 0.33 |
| Model 2c | Moderator |  |  |  |
| Age moderation | Standardized prenatal PSS x child age at outcome | -0.05 | [-0.17, 0.07] | 0.43 |
| Model 3 | Prenatal predictor |  |  |  |
| On the path^c^ | Standardized prenatal PSS | 1.17 | [0.84, 1.51] | <0.0001 |
|  | Covariate |  |  |  |
|  | Household income |  |  |  |
|  | $30,000–$49,999 | -1.30 | [-2.48, -0.12] | 0.03 |
|  | $50,000–$74,999 | -1.54 | [-2.74, -0.34] | 0.01 |
|  | $75,000–$99,999 | -1.71 | [-3.10, -0.31] | 0.02 |
|  | $100,000–$199,999 | -2.31 | [-3.65, -0.98] | <0.001 |
|  | $200,000 or more | -2.56 | [-4.14, -0.98] | 0.002 |
|  | Maternal education |  |  |  |
|  | High school degree or equivalent | -2.01 | [-3.56, -0.46] | 0.01 |
|  | Some college, no degree | -2.48 | [-3.95, -1.01] | <0.001 |
|  | Bachelor’s degree | -2.08 | [-3.66, -0.51] | 0.01 |
|  | Advanced degree and above | -2.55 | [-4.19, -0.91] | 0.002 |
|  | Maternal age at delivery | -0.05 | [-0.12, 0.01] | 0.10 |
|  | Maternal marital status (single) | 0.56 | [-0.35, 1.47] | 0.23 |
|  | Child sex (female) | -1.30 | [-1.90, -0.70] | <0.0001 |
|  | Child age at outcome | 0.62 | [0.45, 0.80] | <0.0001 |
|  | Prenatal smoking | -0.62 | [-2.35, 1.12] | 0.49 |
|  | Any prenatal alcohol use | 0.32 | [-0.52, 1.17] | 0.45 |
|  | Child gestational age at birth | -0.12 | [-0.28, 0.05] | 0.16 |
|  | Maternal depressive symptoms | 0.27 | [0.23, 0.3] | <0.0001 |

*Note*. ECHO Cohort included as random intercept in all models. *B* values are unstandardized beta coefficients. CBCL=Child Behavior Checklist; ECHO=Environmental influences on Child Health Outcomes; PSS=Perceived Stress Scale.

^a^Minimally adjusted model accounted for 11% of variance in child Internalizing Problems.

^b^Fully adjusted model accounted for 11% of variance in child Internalizing Problems.

^c^On-the-path model accounted for 16% of variance in child Internalizing Problems.

**Table S10**

*Regression Models of Maternal Adverse Childhood Experiences (ACEs), Maternal Prenatal Stress, and Child Internalizing Problems Score, N=834*

| Model | Predictor | *B* | 95% CI | *P* |
| --- | --- | --- | --- | --- |
| Model 1 | Childhood predictor |  |  |  |
| Minimally adjusted^a^ | Standardized maternal ACEs | 1.59 | [0.85, 2.33] | <0.0001 |
|  | Prenatal predictor |  |  |  |
|  | Standardized prenatal PSS | 2.11 | [1.36, 2.86] | <0.0001 |
| Model 2a | Childhood predictor |  |  |  |
| Fully adjusted^b^ | Standardized maternal ACEs | 1.38 | [0.64, 2.13] | <0.001 |
|  | Prenatal predictor |  |  |  |
|  | Standardized prenatal PSS | 1.83 | [1.09, 2.57] | <0.0001 |
|  | Covariate |  |  |  |
|  | Household income |  |  |  |
|  | $30,000–$49,999 | -2.72 | [-5.26, -0.19] | 0.04 |
|  | $50,000–$74,999 | -3.73 | [-6.6, -0.87] | 0.01 |
|  | $75,000–$99,999 | -4.44 | [-7.53, -1.34] | 0.005 |
|  | $100,000–$199,999 | -4.20 | [-6.94, -1.46] | 0.003 |
|  | $200,000 or more | -4.19 | [-7.46, -0.93] | 0.01 |
|  | Maternal education |  |  |  |
|  | High school degree or equivalent | -4.54 | [-7.81, -1.27] | 0.007 |
|  | Some college, no degree | -4.08 | [-7.31, -0.86] | 0.01 |
|  | Bachelor’s degree | -2.49 | [-5.89, 0.90] | 0.15 |
|  | Advanced degree and above | -3.60 | [-7.03, -0.18] | 0.04 |
|  | Maternal age at delivery | -0.09 | [-0.23, 0.06] | 0.24 |
|  | Maternal marital status (single) | 2.04 | [-0.01, 4.08] | 0.05 |
|  | Child sex (female) | -0.86 | [-2.27, 0.56] | 0.24 |
|  | Child age at outcome | 0.68 | [0.34, 1.02] | <0.0001 |
| Model 2bi | Moderator |  |  |  |
| Sex moderation | Standardized maternal ACEs x child sex (female) | -0.12 | [-1.54, 1.30] | 0.87 |
| Model 2bii | Moderator |  |  |  |
| Sex moderation | Standardized prenatal PSS x child sex (female) | -0.30 | [-1.71, 1.11] | 0.68 |
| Model 2ci | Moderator |  |  |  |
| Age moderation | Standardized maternal ACEs x child age at outcome | 0.19 | [-0.07, 0.44] | 0.15 |
| Model 2cii | Moderator |  |  |  |
| Age moderation | Standardized prenatal PSS x child age at outcome | 0.07 | [-0.18, 0.33] | 0.58 |
| Model 2d | Moderator |  |  |  |
| ACEs moderation | Standardized maternal ACEs x standardized prenatal PSS | 0.18 | [-0.57, 0.93] | 0.63 |
| Model 3 | Childhood predictor |  |  |  |
| On the path^c^ | Standardized maternal ACEs | 0.83 | [0.09, 1.56] | 0.03 |
|  | Prenatal predictor |  |  |  |
|  | Standardized prenatal PSS | 1.06 | [0.32, 1.81] | 0.005 |
|  | Covariate |  |  |  |
|  | Household income |  |  |  |
|  | $30,000–$49,999 | -2.40 | [-4.82, 0.02] | 0.05 |
|  | $50,000–$74,999 | -3.15 | [-5.92, -0.37] | 0.03 |
|  | $75,000–$99,999 | -4.52 | [-7.52, -1.53] | 0.003 |
|  | $100,000–$199,999 | -3.80 | [-6.50, -1.10] | 0.006 |
|  | $200,000 or more | -3.32 | [-6.56, -0.08] | 0.05 |
|  | Maternal education |  |  |  |
|  | High school degree or equivalent | -5.21 | [-8.36, -2.06] | 0.001 |
|  | Some college, no degree | -4.58 | [-7.71, -1.45] | 0.004 |
|  | Bachelor’s degree | -3.23 | [-6.51, 0.05] | 0.05 |
|  | Advanced degree and above | -4.30 | [-7.63, -0.97] | 0.01 |
|  | Maternal age at delivery | -0.11 | [-0.26, 0.03] | 0.11 |
|  | Maternal marital status (single) | 1.92 | [-0.08, 3.92] | 0.06 |
|  | Child sex (female) | -0.86 | [-2.23, 0.50] | 0.22 |
|  | Child age at Outcome | 0.79 | [0.43, 1.15] | <0.0001 |
|  | Prenatal smoking | -0.78 | [-6.47, 4.91] | 0.79 |
|  | Any prenatal alcohol use | 0.33 | [-2.10, 2.76] | 0.79 |
|  | Child gestational age at birth | -0.24 | [-0.64, 0.17] | 0.25 |
|  | Maternal depressive symptoms | 0.37 | [0.28, 0.46] | <0.0001 |

*Note*. ECHO cohort included as random intercept in all models. *B* values are unstandardized beta coefficients. ACEs=adverse childhood experiences; CBCL=Child Behavior Checklist; ECHO=Environmental influences on Child Health Outcomes; PSS=Perceived Stress Scale.

^a^Minimally adjusted model accounted for 8% of variance in child Internalizing Problems score.

^b^Fully adjusted model accounted for 14% of variance in child Internalizing Problems score.

^c^On-the-path model accounted for 21% of variance in child Internalizing Problems score.

**Table S11**

*Separate Fully Adjusted Logistic Regression Models of Maternal Adverse Childhood Experiences (ACEs) and/or Maternal Prenatal Stress and Binary Child Total Problems Score (T-score ≥60)*

| Model | Predictor | aOR | 95% CI | *P* |
| --- | --- | --- | --- | --- |
| Model 4 | Prenatal predictor |  |  |  |
| N=4,437 | Prenatal PSS | 1.04 | [1.03, 1.05] | <0.001 |
| Model 5 | Childhood predictor |  |  |  |
| N=2,906 | Maternal ACEs | 1.22 | [1.16, 1.29] | <0.001 |
| Model 6 | Childhood predictor |  |  |  |
| N=834 | Maternal ACEs | 1.17 | [1.04, 1.31] | 0.008 |
|  | Prenatal predictor |  |  |  |
|  | Prenatal PSS | 1.05 | [1.02, 1.07] | <0.001 |

*Note*. ECHO cohort included as random intercept in all models. All models are adjusted for household income, maternal education, maternal age at delivery, maternal marital status, child sex, and child age at outcome.

ACEs=adverse childhood experiences; aOR=adjusted odds ratio; ECHO=Environmental influences on Child Health Outcomes; PSS=Perceived Stress Scale.

**Table S12**

*Separate Fully Adjusted Logistic Regression Models of Maternal Adverse Childhood Experiences (ACEs) and/or Maternal Prenatal Stress and Binary Child Externalizing Problems Score (T-score ≥60)*

| Model | Predictor | aOR | 95% CI | *P* |
| --- | --- | --- | --- | --- |
| Model 4 | Prenatal predictor |  |  |  |
| N=4,440 | Prenatal PSS | 1.04 | [1.03, 1.05] | <0.001 |
| Model 5 | Childhood predictor |  |  |  |
| N=2,729 | Maternal ACEs | 1.17 | [1.10, 1.25] | <0.001 |
| Model 6 | Childhood predictor |  |  |  |
| N=834 | Maternal ACEs | 1.14 | [1.00, 1.31] | 0.05 |
|  | Prenatal predictor |  |  |  |
|  | Prenatal PSS | 1.03 | [1.01, 1.06] | 0.02 |

*Note*. ECHO cohort included as random intercept in all models. All models are adjusted for household income, maternal education, maternal age at delivery, maternal marital status, child sex, and child age at outcome.

ACEs=adverse childhood experiences; aOR=adjusted odds ratio; CBCL=Child Behavior Checklist; ECHO=Environmental influences on Child Health Outcomes; PSS=Perceived Stress Scale.

p<0.05, p<0.01, p<0.001

**Table S13**

*Separate Fully Adjusted Logistic Regression Models of Maternal Adverse Childhood Experiences (ACEs) and/or Maternal Prenatal Stress and Binary Child Internalizing Problems (T-score ≥60)*

| Model | Predictor | aOR | 95% CI | *P* |
| --- | --- | --- | --- | --- |
| Model 4 | Prenatal predictor |  |  |  |
| N=4,438 | Prenatal PSS | 1.03 | [1.02, 1.04] | <0.001 |
| Model 5 | Childhood predictor |  |  |  |
| N=2,665 | Maternal ACEs | 1.16 | [1.10, 1.22] | <0.001 |
| Model 6 | Childhood predictor |  |  |  |
| N=834 | Maternal ACEs | 1.13 | [1.02, 1.26] | 0.022 |
|  | Prenatal predictor |  |  |  |
|  | Prenatal PSS | 1.04 | [1.02, 1.06] | 0.001 |

*Note*. ECHO cohort included as random intercept in all models. All models are adjusted for household income, maternal education, maternal age at delivery, maternal marital status, child sex, and child age at outcome.

ACEs=adverse childhood experiences; aOR=adjusted odds ratio; ECHO=Environmental influences on Child Health Outcomes; PSS=Perceived Stress Scale.

**Figure S1**

*Flowchart of Sample Selection*


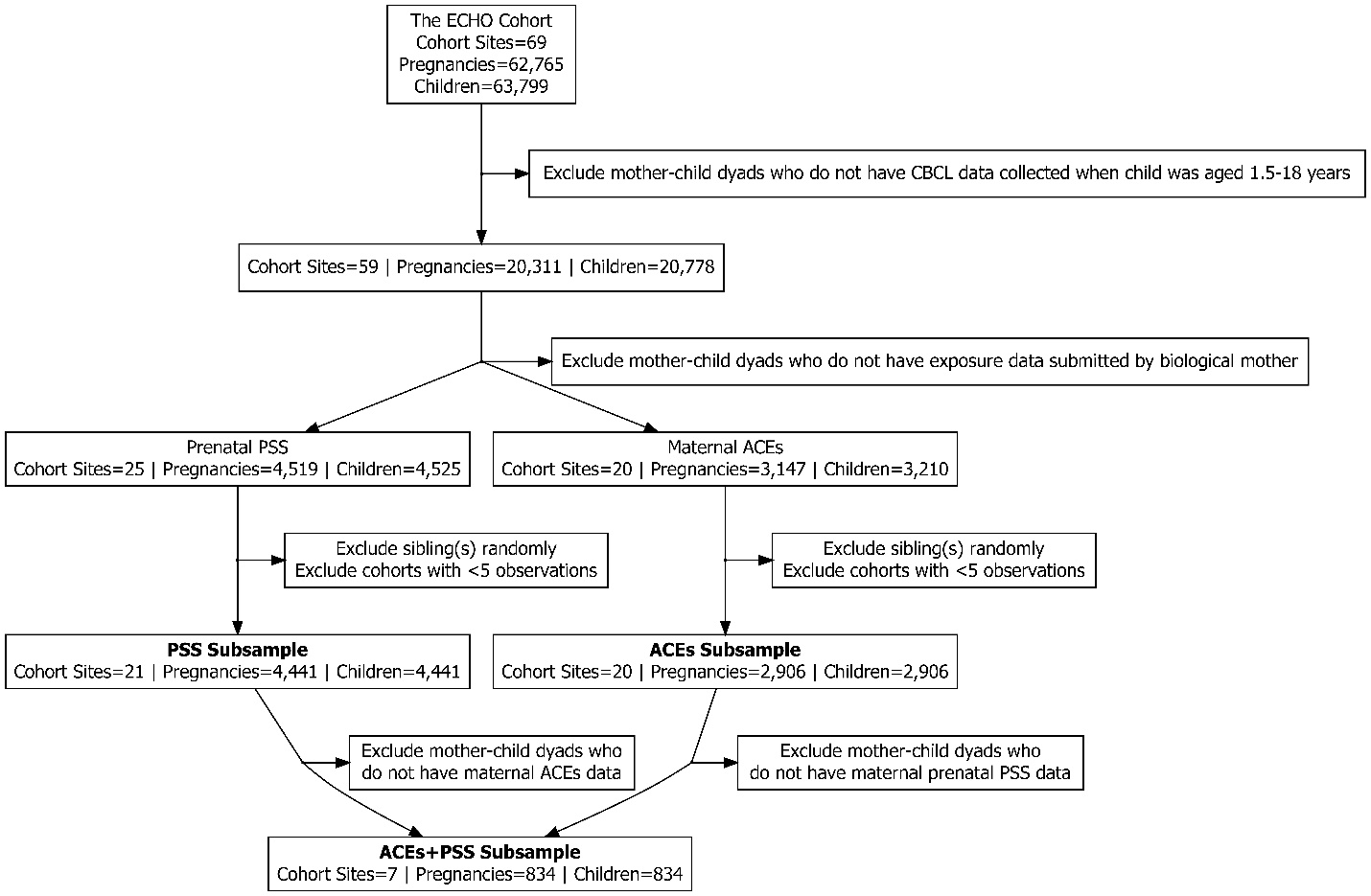


*Note*. ACEs=adverse childhood experiences; CBCL=Child Behavior Checklist; ECHO=Environmental influences on Child Health Outcomes; PSS=Perceived Stress Scale.

**Figure S2**

*Map of Participating Sites in the Environmental influences on Child Health Outcomes (ECHO) Cohort*

**
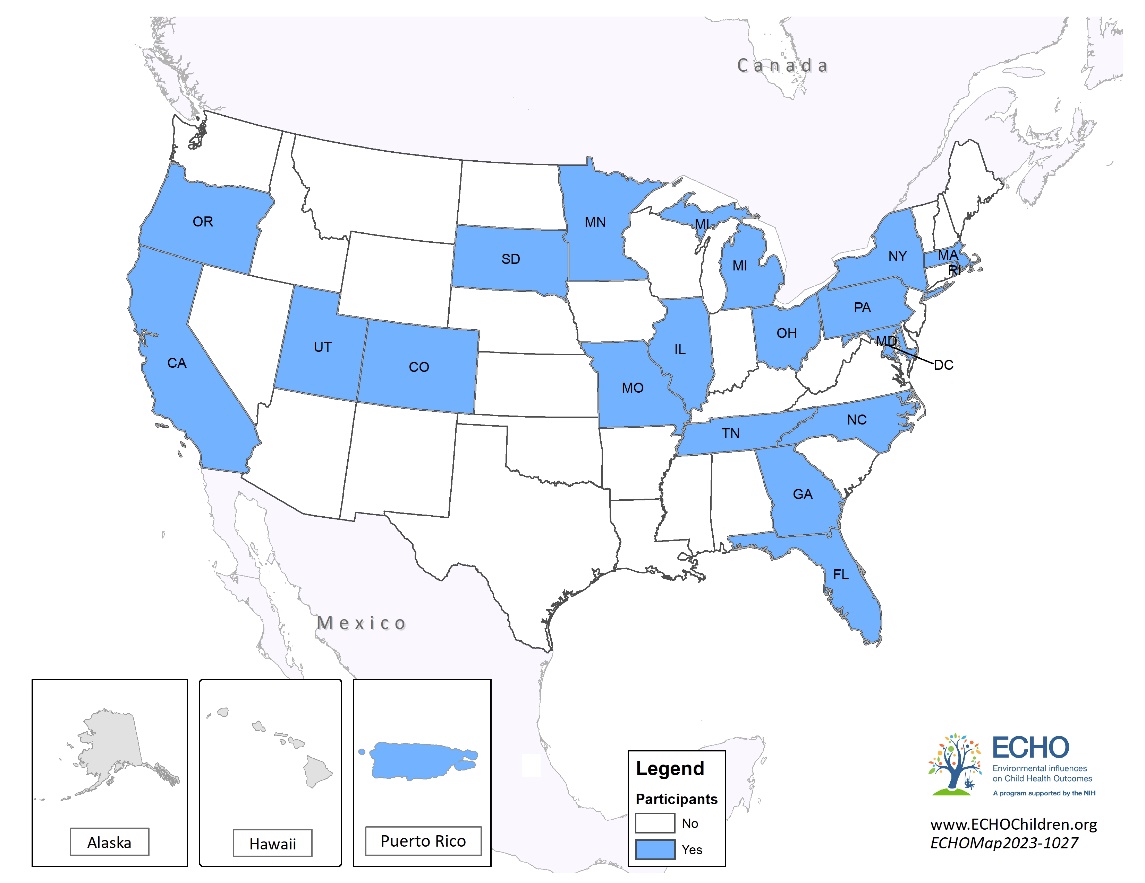
**

**Figure S3**

*Histogram of child ages at outcome assessment in ACEs and PSS subsamples*

**
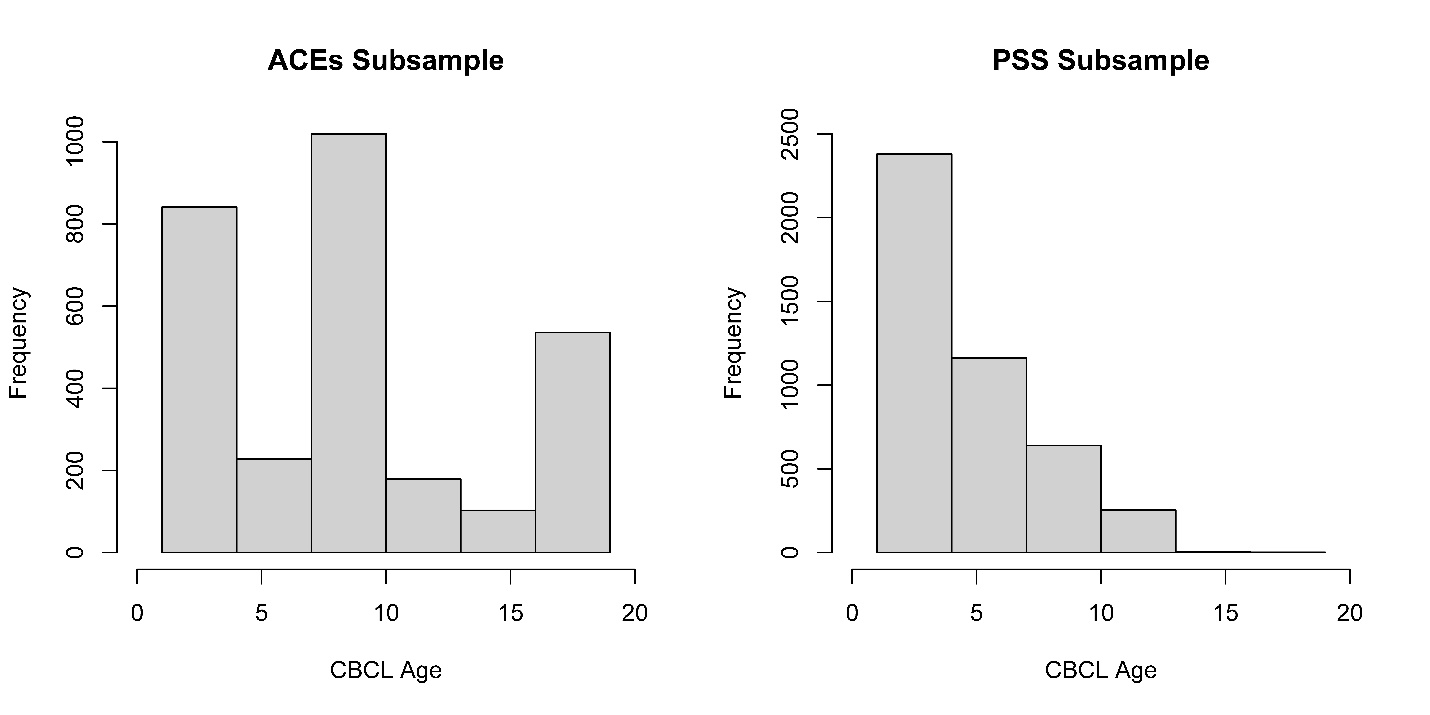
**

**Figure S4**

*Dose-Response Relation Between Maternal Adverse Childhood Experiences (ACEs) and Child Total Problems Score*


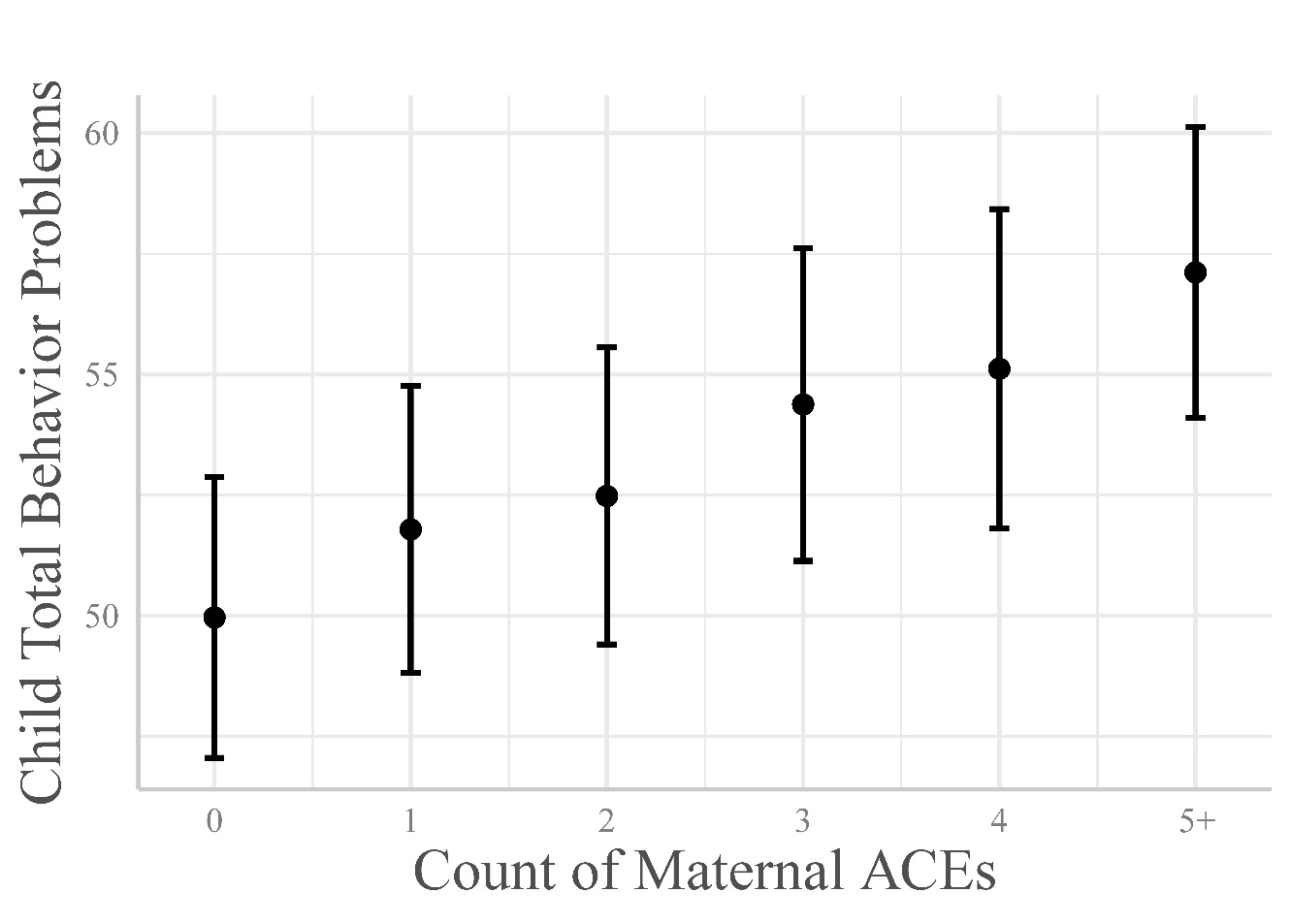

Supplement: Ahmad et al. supplementary material [file S0033291725103127sup001.zip › EC0639_Supplement 1_Psychol Med_R1_08_12_25.docx]
