# Supplementary material for: Maternal adverse childhood experiences and prenatal stress: Intergenerational transmission and offspring mental health in the ECHO Cohort
Source: Psychol Med. 2026 Mar 11;56:e60. doi: 10.1017/S0033291725103127 (PMC13040406; doi:10.1017/S0033291725103127)
Supplement: Ahmad et al. supplementary material [file S0033291725103127sup001.zip › EC0639 Supplement 2.pdf]

ECHO Environmental influences on Child Health Outcomes A program supported by the NIH	**Adverse Childhood Experiences - Adult Primary Version**<br>ECHO-wide Cohort Version 01.21 / January 17, 2019	**Form ACE-aPV**<br>Page 1 of 2

COHORT ID	SITE ID	PARTICIPANT ID	PIN	COHORT VISIT ID	FORM COMPLETED
__ __ __ __ __	__ __ __ __	______________	__ __ __	__________	__ __/__ __/__ __ __ __<br>mm dd yyyy

ECHO LIFE STAGE		RESPONDENT	
☐01 Prenatal	☐02 Perinatal	☐01 Participant	☐02 Biological Mother
☐03 Infancy	☐04 Early Childhood	☐03 Biological Father	☐04 Other Respondent → Code: __ __
☐05 Middle Childhood	☐06 Adolescence		

STUDY STAFF INSTRUCTION: This form, or the alternate version, should be completed once, by either the pregnant woman during the prenatal life stage using the woman's pregnancy ID or by the child's primary caregiver during the infancy, early childhood, middle childhood, or adolescence life stages using the child's participant ID.

INSTRUCTIONS: *Please answer each question. Your answers will be kept confidential and used for research purposes only.*

While you were growing up, during your first 18 years of life …

1. Did a parent or other adult in the household **often** swear at you, insult you, put you down, or humiliate you?
   - ☐01 Yes → ***SKIP TO 2***
   - ☐02 No

   a. Did a parent or other adult in the household **often** act in a way that made you afraid that you might be physically hurt?
   - ☐01 Yes
   - ☐02 No

2. Did a parent or other adult in the household **often** push, grab, slap, or throw something at you?
   - ☐01 Yes → ***SKIP TO 3***
   - ☐02 No

   a. Did a parent or other adult in the household **ever** hit you so hard that you had marks or were injured?
   - ☐01 Yes
   - ☐02 No

3. Did an adult or person at least 5 years older than you **ever** touch or fondle you or have you touch their body in a sexual way?
   - ☐01 Yes → ***SKIP TO 4***
   - ☐02 No

   a. Did an adult or person at least 5 years older than you **ever** attempt or actually have oral, anal, or vaginal intercourse with you?
   - ☐01 Yes
   - ☐02 No

4. Did you **often** feel that no one in your family loved you or thought you were important or special?
   - ☐01 Yes → ***SKIP TO 5***
   - ☐02 No

   a. Did you **often** feel that your family didn't look out for each other, feel close to each other, or support each other?
   - ☐01 Yes
   - ☐02 No

While you were growing up, during your first 18 years of life …

5. Did you **often** feel that you didn't have enough to eat, had to wear dirty clothes, and had no one to protect you?

☐01 Yes → ***SKIP TO 6***
☐02 No

a. Did you **often** feel that your parents were too drunk or high to take care of you or take you to the doctor if you needed it?

☐01 Yes
☐02 No

6. Were your parents **ever** separated or divorced?

☐01 Yes
☐02 No

7. Was your mother or stepmother **often** pushed, grabbed, slapped, or had something thrown at her?

☐01 Yes → ***SKIP TO 8***
☐02 No

a. Was your mother or stepmother **sometimes or often** kicked, bitten, hit with a fist, or hit with something hard?

☐01 Yes → ***SKIP TO 8***
☐02 No

b. Was your mother or stepmother **ever** repeatedly hit at least a few minutes or threatened with a gun or knife?

☐01 Yes
☐02 No

8. Did you live with anyone who was a problem drinker or alcoholic or who used street drugs?

☐01 Yes
☐02 No

9. Was a household member depressed or mentally ill, or did a household member attempt suicide?

☐01 Yes
☐02 No

10. Did a household member go to prison?

☐01 Yes
☐02 No

Setting			Mode	
☐01 Clinic or site	☐02 Phone	☐03 Other location	☐01 Self-administered	☐02 Staff-administered
