# Supplementary material for: Maternal adverse childhood experiences and prenatal stress: Intergenerational transmission and offspring mental health in the ECHO Cohort
Source: Psychol Med. 2026 Mar 11;56:e60. doi: 10.1017/S0033291725103127 (PMC13040406; doi:10.1017/S0033291725103127)
Supplement: Ahmad et al. supplementary material [file S0033291725103127sup001.zip › EC0639 Supplement 3.pdf]

Adverse Childhood Experiences – Adult Alternate Version

ECHO-wide Cohort Version 01.20 / November 30, 2018

Form ACE-aAV

COHORT ID	SITE ID	PARTICIPANT ID	PIN	COHORT VISIT ID	FORM COMPLETED
__ __ __ __ __	__ __ __ __	____________	__ __ __	________	__ __/__ __/__ __ __ __ mm dd yyyy

ECHO LIFE STAGE	RESPONDENT
☐01 Prenatal ☐02 Perinatal ☐03 Infancy ☐04 Early Childhood ☐05 Middle Childhood ☐06 Adolescence	☐01 Participant ☐02 Biological Mother ☐03 Biological Father ☐04 Other Respondent → Code: __ __

STUDY STAFF INSTRUCTION: This form, or the primary version, should be completed once, by either the pregnant woman during the prenatal life stage using the woman's pregnancy ID or by the child's primary caregiver during the infancy, early childhood, middle childhood, or adolescence life stages using the child's participant ID.

INSTRUCTIONS: *Please answer each question. Your answers will be kept confidential and used for research purposes only.*

1. Please read the **seven** statements below. **Count** the number of statements that apply to you. Please **DO NOT** mark or indicate which specific statements apply to you.

While you were growing up, during your first 18 years of life:

- A parent or other adult in the household **often** swore at you, insulted you, put you down, humiliated you, or acted in a way that made you afraid that you might be physically hurt.
- A parent or other adult in the household **often** pushed, grabbed, slapped, or threw something at you or **ever** hit you so hard that you had marks or were injured.
- An adult or person at least 5 years older than you **ever** touched or fondled you, had you touch their body in a sexual way, or attempted or actually had oral, anal, or vaginal intercourse with you.
- Your parents were **ever** separated or divorced.
- You lived with someone who was a problem drinker or alcoholic or who used street drugs.
- A household member was depressed, mentally ill, or attempted suicide.
- A household member went to prison.

Of the statements above, how many apply to you?

☐00 0
☐01 1
☐02 2
☐03 3
☐04 4
☐05 5 or more

While you were growing up, during your first 18 years of life …

2. Did you **often** feel that no one in your family loved you or thought you were important or special?

☐01 Yes → ***SKIP TO 3***
☐02 No

a. Did you **often** feel that your family didn't look out for each other, feel close to each other, or support each other?

☐01 Yes
☐02 No

3. Did you **often** feel that you didn't have enough to eat, had to wear dirty clothes, and had no one to protect you?

☐01 Yes → ***SKIP TO 4***
☐02 No

a. Did you **often** feel that your parents were too drunk or high to take care of you or take you to the doctor if you needed it?

☐01 Yes
☐02 No

4. Was your mother or stepmother **often** pushed, grabbed, slapped, or had something thrown at her?

☐01 Yes → ***SKIP TO END***
☐02 No

a. Was your mother or stepmother **sometimes or often** kicked, bitten, hit with a fist, or hit with something hard?

☐01 Yes → ***SKIP TO END***
☐02 No

b. Was your mother or stepmother **ever** repeatedly hit for at least a few minutes or threatened with a gun or knife?

☐01 Yes
☐02 No

Setting			Mode	
☐01 Clinic or site	☐02 Phone	☐03 Other location	☐01 Self-administered	☐02 Staff-administered

Modified from the original CDC-Kaiser ACEs Study. See https://www.cdc.gov/violenceprevention/acestudy/index.html. Methods/development article: Felitti VJ, Anda RF, Nordenberg D, Williamson DF, Spitz AM, Edwards V, Marks JS. Relationship of childhood abuse and household dysfunction to many of the leading causes of death in adults: The Adverse Childhood Experiences (ACE) Study. *American Journal of Preventive Medicine*. 1998 May 1;14(4):245-58.
